# The role of selection in the evolution of marine turtles mitogenomes

**DOI:** 10.1038/s41598-020-73874-8

**Published:** 2020-10-12

**Authors:** Elisa Karen da Silva Ramos, Lucas Freitas, Mariana F. Nery

**Affiliations:** Laboratório de Genômica Evolutiva, Departamento de Genética, Evolução, Microbiologia e Imunologia, Universidade Estadual de Campinas, Cidade Universitária, Campinas, SP 13083970 Brazil

**Keywords:** Evolution, Molecular evolution

## Abstract

Sea turtles are the only extant chelonian representatives that inhabit the marine environment. One key to successful colonization of this habitat is the adaptation to different energetic demands. Such energetic requirement is intrinsically related to the mitochondrial ability to generate energy through oxidative phosphorylation (OXPHOS) process. Here, we estimated Testudines phylogenetic relationships from 90 complete chelonian mitochondrial genomes and tested the adaptive evolution of 13 mitochondrial protein-coding genes of sea turtles to determine how natural selection shaped mitochondrial genes of the Chelonioidea clade. Complete mitogenomes showed strong support and resolution, differing at the position of the Chelonioidea clade in comparison to the turtle phylogeny based on nuclear genomic data. Codon models retrieved a relatively increased *dN/dS* (ω) on three OXPHOS genes for sea turtle lineages. Also, we found evidence of positive selection on at least three codon positions, encoded by NADH dehydrogenase genes (*ND4* and *ND5*). The accelerated evolutionary rates found for sea turtles on *COX2*, *ND1* and *CYTB* and the molecular footprints of positive selection found on *ND4* and *ND5* genes may be related to mitochondrial molecular adaptation to stress likely resulted from a more active lifestyle in sea turtles. Our study provides insight into the adaptive evolution of the mtDNA genome in sea turtles and its implications for the molecular mechanism of oxidative phosphorylation.

## Introduction

How different environmental conditions shape mitochondrial DNA evolution has become a common question in the field of evolutionary biology, revealing more about adaptive patterns in organisms facing ecological changes than expected. Mitochondria is a cellular component directly involved in oxygen use, metabolism, and energy production, and hence plays an important role in aerobic respiration through oxidative phosphorylation (OXPHOS)^1^. The mitogenome is composed of 13 genes encoding OXPHOS proteins, 2 rRNAs (12S rRNA and 16S rRNA), and 22 tRNAs^2,3^, and is thought to evolve under continuous purifying selection for coding regions^4,5^. However, as mitochondrial genes respond to changes in energy requirements, extreme environments may favor positive selection, driving adaptations in different mitogenome genes for some lineages^5–8^.

Selection in mtDNA genes has been linked to environmental temperature, high demanding metabolism, altitude and oxygen availability in several species^7,9–12^. Among extreme environmental conditions, research on the molecular evolution of mtDNA related to high altitude has received special attention^6,13–16^, mainly because evaluating selective pressures of environmental temperature and oxygen availability on mtDNA molecular changes could provide key insights on mitogenome adaptive evolution^14^. Aquatic environments present similar challenging conditions (hypoxia and low temperatures), and accordingly, also demands mitogenome adaptations in vertebrates, which were reported for killer whales^17^, river dolphins^18^, and penguins^11^. Despite most researches on the role of mtDNA selection in endothermic organisms evolution, recently there has been an increased interest in ectothermic species which are highly dependent on environmental conditions. For instance, Escalona et al.^19^ studied mitochondrial evolution on softshell turtles revealing positively selected sites in complex I genes for Trionychidae clade and *Carettochelys insculpta*, suggesting convergent evolution of OXPHOS genes in response to a long-lasting aquatic lifestyle in both lineages.

Chelonians are a group with more than 300 species distributed in diverse ecological niches around the world, including rivers, lakes, forests, deserts and oceans^20^. Among chelonians, several lineages independently adapted to the marine environment and only the representatives of the Chelonioidea clade (extant sea turtles) have survived to the present (see Evers and Benson^21^). Sea turtles comprise seven extant species grouped into two sister families, Dermochelyidae (one species) and Cheloniidae (six species)^22^. These species present adaptations to marine environmental challenges, such as flippers, high salt excretion by modified lachrymal glands^23^, hydrodynamic shells, and cardiorespiratory adaptations to deliver O_2_ to tissues during dives^24^. In addition, sea turtles have an active lifestyle, being diving organisms, highly migratory, and with high fecundity rates^25,26^. Marine turtles are among the fastest moving extant reptiles and, since energy demand for locomotion is considered the primary determinant of metabolic rate for marine organisms^27^, the active lifestyle of marine turtles is expected to affect their energetic demands, resulting in larger metabolic rates compared to other reptiles^25,26,28^. Therefore, it is reasonable to hypothesize that mitochondrial gene adaptations may have had an important role in the adaptive success of sea turtles lineages in the marine environment, leaving molecular footprints in their mitogenomes.

Despite previous works on the characterization of sea turtles mitogenomes, mainly used in phylogenetic and phylogeographic approaches, only exploratory analysis has examined potential adaptations to the marine environment in these species at the molecular level^29^. Here we aimed to investigate the evolutionary patterns of sea turtle mitogenomes and address the possible role of mtDNA evolution in the adaptation to metabolic energetic high demands on these species. We provided a comprehensive phylogenetic analysis of mitogenome evolution in Testudines and investigated the evolutionary rates and molecular signatures of natural selection for all 13 mtDNA protein-coding genes for sea turtles, in a phylogenetic comparison against lineages of non-marine turtles and other non-avian reptiles.

## Results

### mtDNA genomes of sea turtles

Assembly for the 90 mitogenomes yielded complete mitogenome lengths between 16,386 and 21,933 bp. All 39 regions common to vertebrate mitogenomes were identified highlighting the *ND5* translocation in the Platysternidae family (Supplementary Fig. S1 online). Estimates of genetic diversity from protein-coding genes (PCGs) retrieved *ATP8* as the most diverse gene among all species (π = 0.156) followed by NADH dehydrogenase genes with *ND6* (π = 0.133) presenting higher diversity. The cytochrome c oxidase genes (*COX1*, *COX2*, and *COX3*) were the least diverse among the mtDNA genes (Table 1).

**Table 1:** Interspecific diversity for all 13 protein-coding genes on sea turtles.

Gene	Complex	bp	S	π
*ATP6*	V	681	182	0.113
*ATP8*	V	231	51	0.156
*COX1*	IV	1662	299	0.087
*COX2*	IV	696	143	0.089
*COX3*	IV	807	150	0.085
*CYTB*	III	1200	267	0.097
*ND1*	I	981	226	0.098
*ND2*	I	1086	248	0.102
*ND3*	I	390	104	0.129
*ND4*	I	1395	367	0.110
*ND4L*	I	306	70	0.100
*ND5*	I	1914	455	0.109
*ND6*	I	573	168	0.133

Polymorphic sites (S), nucleotide diversity (π).

### Phylogenetic reconstruction

**Figure 1:**
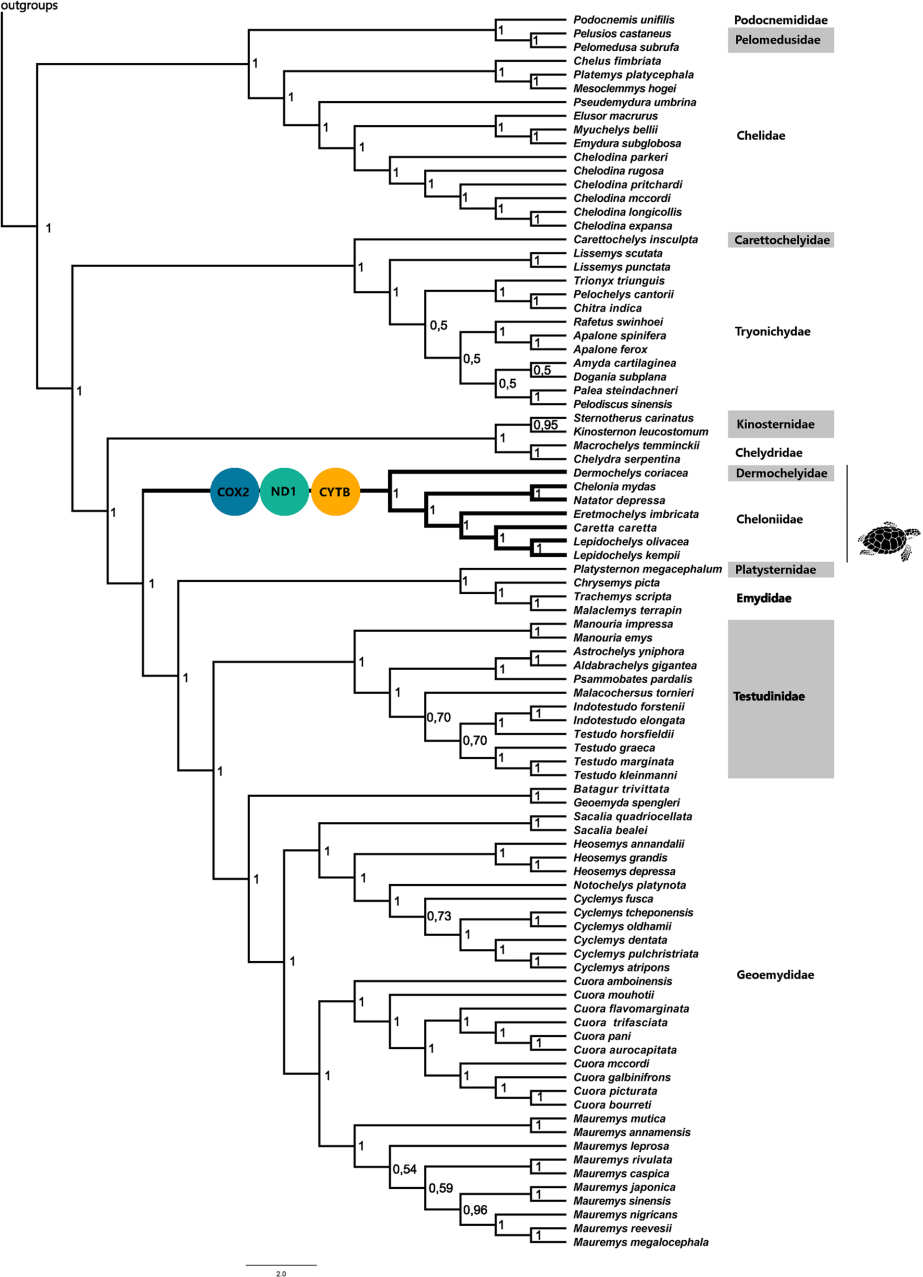
Bayesian tree (dataset II) for testudines mitogenomes and topology used to analyze the selective pressures on Chelonioidea. Circles represent mitochondrial genes with higher *dN/dS* (ω) for Chelonioidea against other chelonian lineages identified by branch model on codeML. The number on nodes represent Bayesian posterior probabilities.

ML and Bayesian inferences recovered highly similar and well-resolved topologies for an alignment of complete mitogenomes for the 110 species (dataset I) and an alignment of the 13 concatenated PCGs for the 110 species (dataset II) with high bootstrap (BP > 50%) and posterior probabilities (PP > 90%) support for most branches (Supplementary Fig. S2 online). Few differences in topologies of different datasets (I × II) obtained by different methods (Bayesian × ML) were found, focusing on the relationships among few species of Trionychidae, Testudinidae, and Geoemydidae (especially *Mauremis* group) families (Supplementary Fig. S2 online). Because ML and Bayesian approaches for both datasets yielded highly similar topologies, subsequent analyses were conducted using the Bayesian tree of dataset II (Fig. 1).

### Selection analyses

#### Branch model

**Figure 2:**
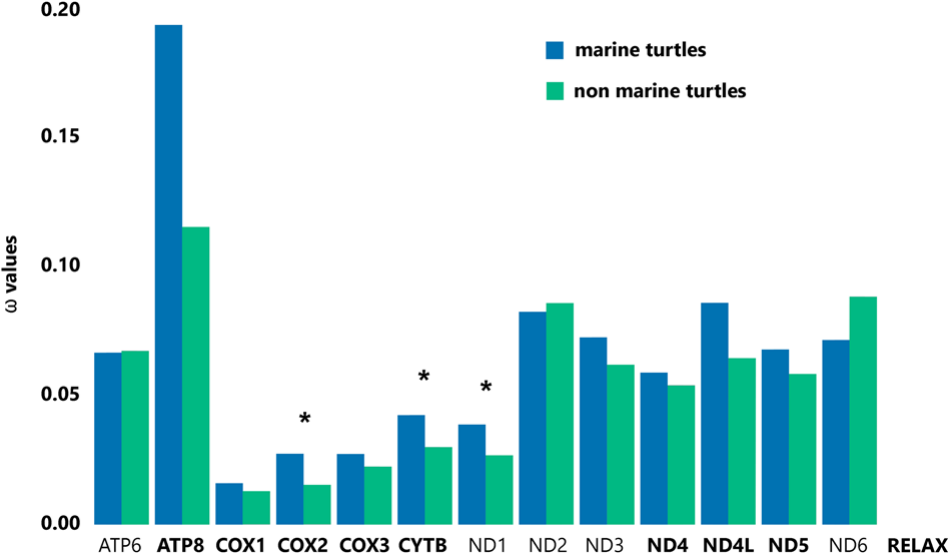
Natural selection strength and the ratio of non-synonymous to synonymous substitutions (ω) calculated with two-ratio model in codeML for the 13 protein-coding mitochondrial genes (dataset III) of marine (Chelonioidea clade) and non-marine chelonians (all other chelonian clades). Genes in which the two-model fits better to the data (*p* < 0.05) are marked with an asterisk and were estimated with the two-ratio branch model in the PAML program. Gene names in bold represent genes with significant intensified selection on marine turtles detected by RELAX (*p* < 0.05).

To test the role of selection on the Chelonioidea branch in the Testudines phylogenetic tree of the 13 PCGs we applied codeML branch model tests. The free-ratio model fit our data significantly better than the null hypothesis (one-ratio model) for all 13 PCGs, suggesting different evolutionary rates among lineages included in our dataset. Values of *dN*, *dS* and ω for each branch are available on Supplementary Data S1 online. The two-ratio model, used to calculate selective pressures acting on sea turtle lineage, fitted better for genes *COX2*, *ND1* and *CYTB* when the sea turtle lineage was labeled as foreground branch (Fig. 2). Also, the *ATP8* gene showed the highest ω values, both for the foreground and background branches, when compared to other genes. However, the LRT was unable to indicate the two-ratio model as the model with best fit to explain the differences in ω values for this gene (*p* = 0.09) (Supplementary Table S3 online). Moreover, RELAX detected significant intensified selection (K > 1) in *ATP8, COX1*, *COX2*, *COX3*, *CYTB*, *ND4*, *ND4L*, and *ND5* genes for the sea turtle lineage (Fig. 2 and Supplementary Table S4 online) and no genes were identified under relaxation.

#### Branch-site models

To calculate selective pressures acting only on the sea turtle lineage from chelonian phylogeny we performed branch-site models using codeML, FITMODEL, and aBSREL. The branch-site tests carried with codeML and FITMODEL detect significant signals of positive selection for a few proportions of the genes tested in sea turtle lineage (Supplementary Table S5 online), corresponding for only three sites under selection on *ND4* and *ND5* genes (Table 2). We applied four codon substitution models (M0, M3, M3 + S1, and M3 + S2) in FITMODEL (Guindon et al. 2004). The LRT between nested models (M0 x M3; M3 x M3 + S1; M3 + S1 x M3 + S2) suggested that the M3 + S2 model fits better for all 13 PCGs, considering switches between selection patterns at individual sites in Testudines phylogeny (Supplementary Table S6 online). Moreover, this test indicates the action of positive selection on codon 36 of *ND4* gene in the sea turtle branch (Table 2 and Supplementary Figs. S3 and S4 online). aBSREL reports two branches under episodic diversifying selection pressure in the Chelonioidea clade: positive selection on the ancestral branch of Chelonioidea lineages for 4.2% of sites on *ND5* gene, and positive selection on *Eretmochelys imbricata* lineage for 24% of sites on *ND6* gene (Table 2).

**Table 2:** Codon positions under positive selection detected by branch-site model using codeML, FITMODEL, and aBSREL for dataset III.

Marker	codeML	FITMODEL	aBSREL	Lineage
	Positive selected sites (PP)	Positive selected sites	% of positive selected sites	
ATP6	7 L (0.84)	0.0	0.0	–
ATP8	0.0	0.0	0.0	–
COX1	0.0	0.0	0.0	–
COX2	0.0	0.0	0.0	–
COX3	55 A (0.57)	0.0	0.0	Chelonioidea
CYTB	98 V (0.74)	0.0	0.0	Chelonioidea
ND1	0.0	0.0	0.0	–
ND2	0.0	0.0	0.0	–
ND3	0.0	0.0	0.0	–
ND4	169 Q (0.96*)	36 (> 0.90*)	0.0	Chelonioidea
ND4L	0.0	0.0	0.0	–
ND5	185 L (0.98*), 511 V (0.67), 596 L (0.88)	0.0	4.2%	Chelonioidea ancestral branch
ND6	19–(0.67), 247 S (0.74)	0.0	24%	*Eretmochelys imbricata*

The Chelonioidea clade was selected as a foreground in all analyses. Significance was assessed by BEB (Posterior probability (PP) > 0.90) for codeML, PP > 90 in FITMODEL, and *p* value < 0.05 in aBSREL. Lineage column refers to the lineage where the respective site under selection was found under selection. Bold numbers represent statistically significant results.

#### Site models

**Figure 3:**
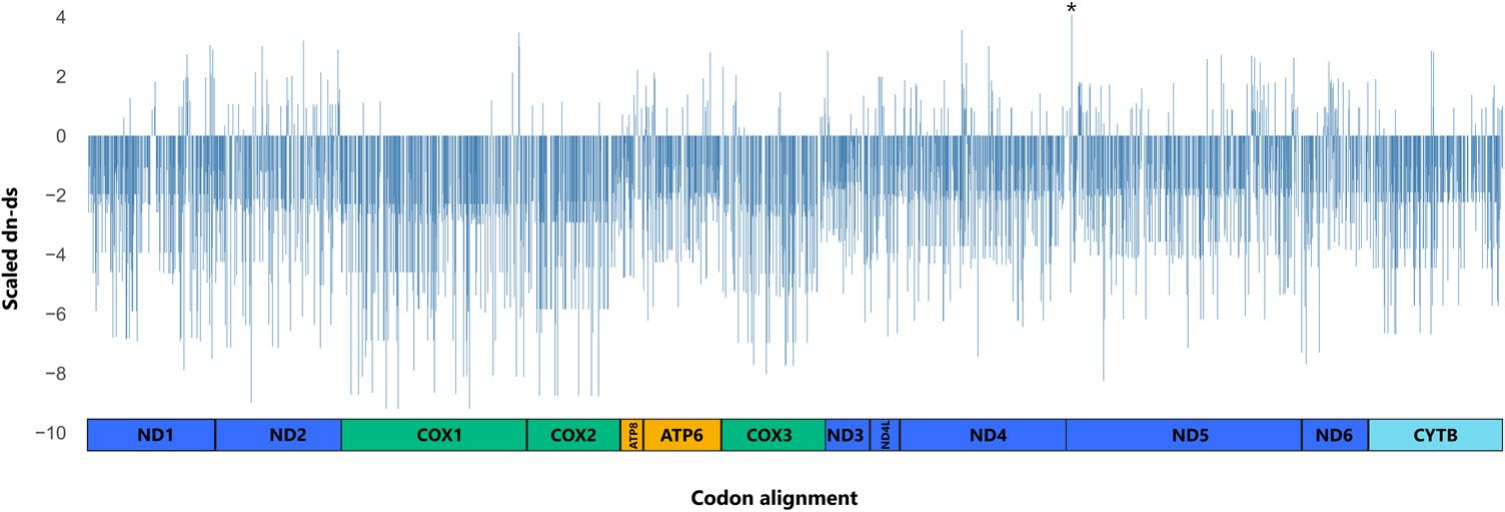
Signatures of selection for each codon for 13 mtDNA genes using SLAC method within the Chelonioidea phylogeny (dataset IV). Positive (positive values) and negative selection (negative values) are shown. *ND6* is transcribed from the light strand. * is the third codon position of *ND5* found under positive selection by FUBAR and MEME.

To further assess the sites under selection inside Chelonioidea we performed positive selection analyes using site models on FITMODEL, SLAC, MEME, and FUBAR (Table 3). FITMODEL retrieved three positively selected sites on *ATP6* and *ND4* genes (Table 3). SLAC returned many codons with high ω values (> 1), but with no significant signatures of positive selection. On SLAC results, the third position of the *ND5* gene stands out with high ω value (ω = 4.06 and *p* = 0.085) (Fig. 3). MEME revealed diversifying and episodic positive selection for *ND4*, and *ND4L* on three codon positions on lineages inside the Chelonioidea branch (Table 3 and Supplementary Table S7 online). As expected, the FUBAR test retrieved a general purifying selection pattern in several codons for all PCGs. *ND5* was the gene with the highest number of codons under purifying selection (42.86%) while *ND6* with the lowest number (10.92%). Only two positions were assigned with diversifying positive selection with FUBAR tests: the codon 3 from *ND5* (PP = 0.92) and the codon 169 from *ND4* (PP = 0.95) (Table 3 and Supplementary Table S8 online).

**Table 3:** Codon positions under positive selection detected by site model using FUBAR, MEME, and FITMODEL for dataset IV.

Marker	FUBAR	MEME	FITMODEL	Lineage
	Positive selected sites	Positive selected sites	Positive	
**Selected sites**				
ATP6	**7 L (0.84)**	179 (> 0.05)	7 (> 0.9*)	*Chelonia mydas* + *Natator depressus*
ATP8	0.0	**47 (> 0.05)**	**47 (> 0.85)**	Cheloniidae
COX1	0.0	0.0	0.0	–
COX2	0.0	0.0	0.0	–
COX3	55 A (0.57)	0.0	0.0	
CYTB	98 V (0.74)	0.0	19 (> 0.85)	*Caretta caretta*
ND1	0.0	0.0	0.0	–
ND2	0.0	0.0	131 (> 0.76)	*Chelonia mydas*
ND3	0.0	0.0	0.0	–
ND4	169 (0.95*)	181 (< 0.05*)	169 (> 0.90*) **181 (> 0.76)**	Cheloniidae *Erytmochelys imbricata*
ND4L	0.0	58 (< 0.05*)	0.0	–
ND5	3 (0.92*)	3 (0.05*)	0.0	–
ND6	0.0	0.0	7 (> 0.76)	*Dermochelys coriacea*

Significance was assessed by PP > 90 in FITMODEL and FUBAR, and *p* value < 0.05 in MEME. The lineage column refers to the lineage where the respective site was found under selection by FITMODEL. Bold numbers represent sites recovered by more than one method.

*Statistically significant results.

**Figure 4:**
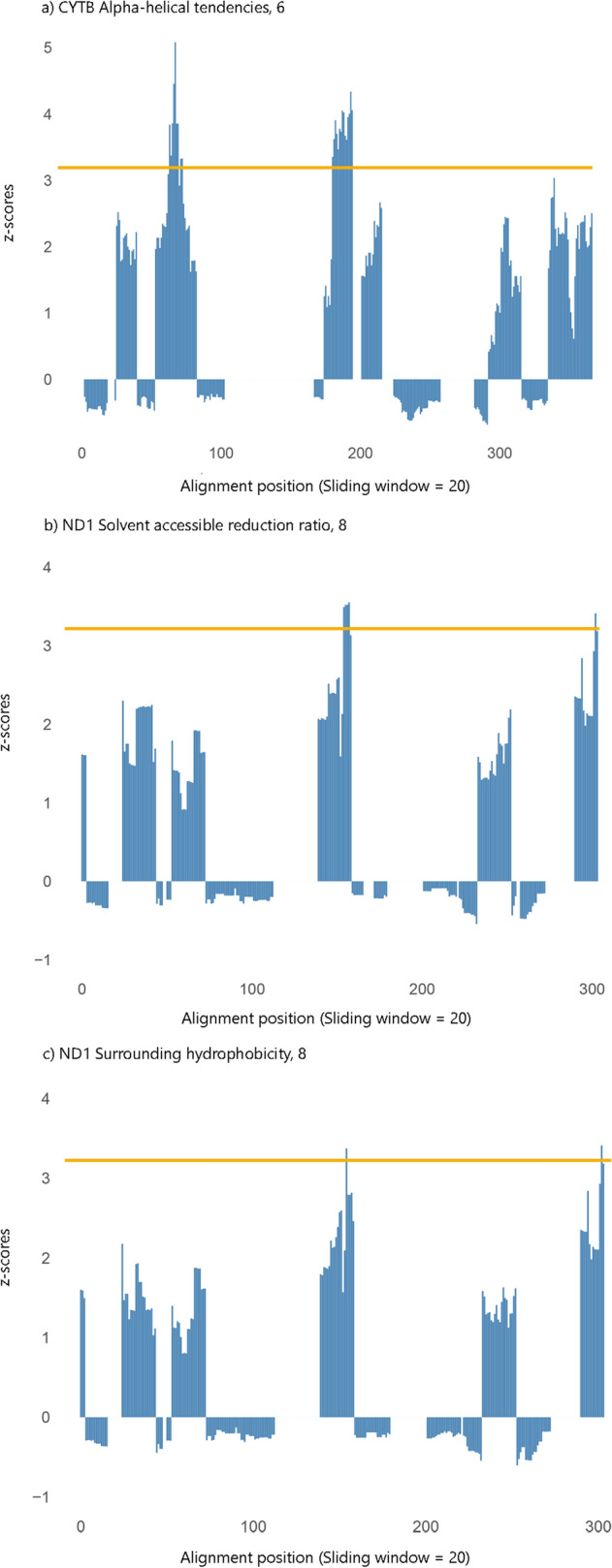
Detection of significant physicochemical amino acids changes using TreeSAAP within the Chelonioidea phylogeny (dataset IV). This analysis was performed on the genes that present higher ω values identified by codeML analysis on the Chelonioidea clade. Regions above the z-score of 3.09 (yellow line) were significantly different than assumed under neutrality. Respective property and category are shown above graphs.

Changes in amino acid physicochemical properties caused by replacements across the phylogeny estimated using TreeSAAP suggested a prevalence of purifying selection for the three genes with a significant difference in the rate of ω between Chelonioidea clade and other chelonian branches identified on the two model ratio test with codeML. Nevertheless, we found evidence for positive selection in four physicochemical properties, with global z-scores > 3.09 (*p* < 0.001) for *ND1* gene [Solvent accessible reduction ratio; Surrounding hydrophobicity; Power to be at the middle of alpha-helix, and Equilibrium constant (ionization of COOH)] and in one physicochemical property for *CYTB* gene (Alpha-helical tendencies) (Fig. 4). Functional analysis reveals that the positively selected 36th codon position is located within the catalytic domain for oxidoreductase activity of *ND4* subunit gene (Supplementary Fig. S3 online).

## Discussion

**Figure 5:**
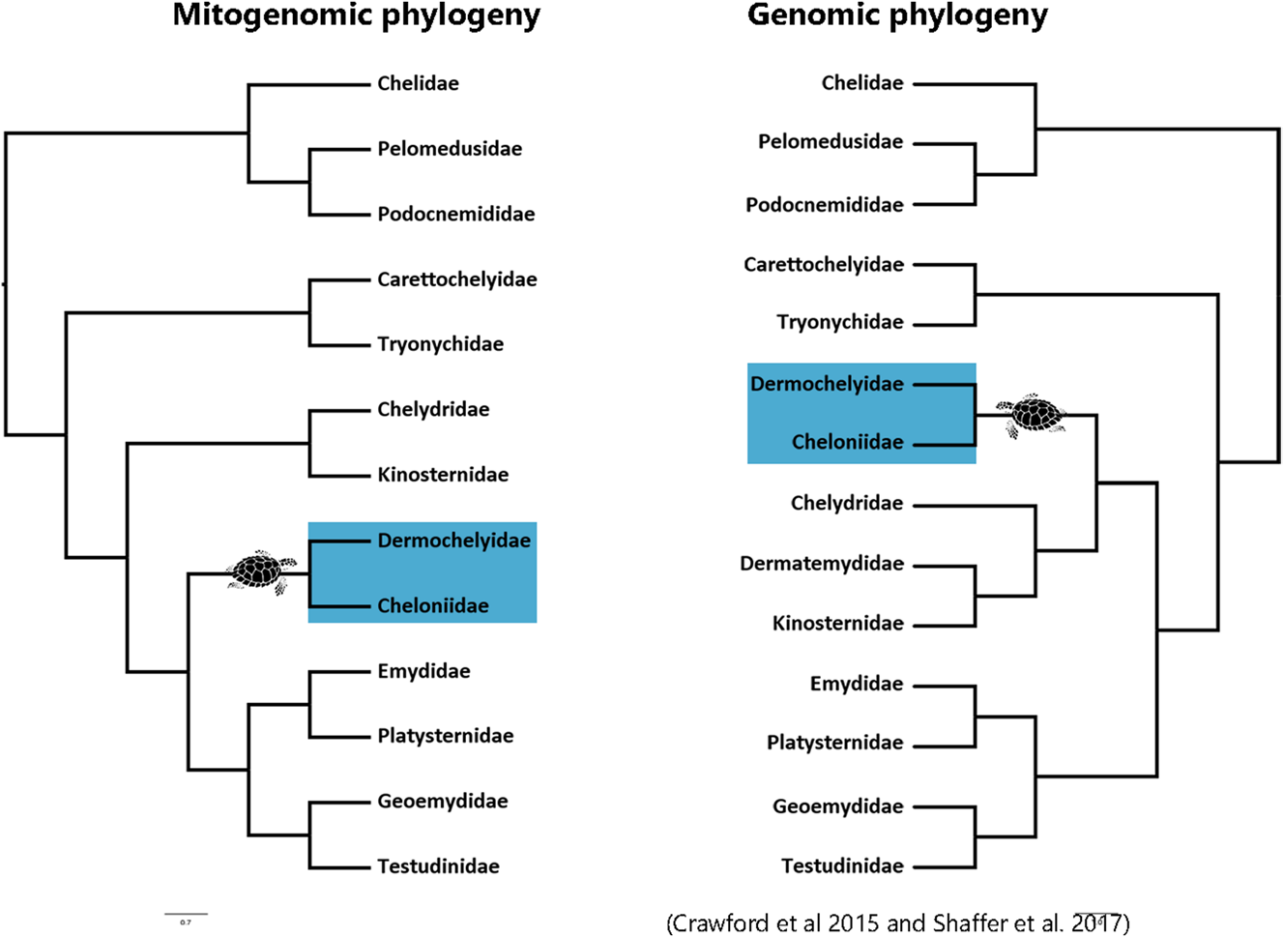
Mitochondrial and nuclear discordant phylogenies for Testudines families.

In this study we present the most comprehensive analysis of mitogenome evolution in Testudines, including data for 90 species, focusing on the molecular evolution of OXPHOS genes in sea turtles. As the data we compiled is derived from publicly available sources, no representative of the Dermatemydidae family was included in the phylogenetic analyses. Thus, the generation of the *Dermatemys mawii* mitogenome is important for achieving a complete mitochondrial phylogeny. Besides the lack of this mitogenome, the phylogenetic relationships we recovered were congruent with a recent chelonian phylogeny based on 57 complete mitochondrial turtle genomes^19^ but with incongruences regarding the positions of Chelydridae + Dermatemyidae + Kinosternidae lineage and the sea turtles lineage (Cheloniidae + Dermochelyidae) when compared to recent phylogenies using nuclear genes^30,31^ (Fig. 5). Several reasons could explain divergences on lineage relationships in mtDNA and nuDNA phylogenies: (1) incomplete lineage sorting, most common to nuDNA than for mtDNA due to the smaller effective population size of mtDNA genome^4,32,33^ (2) sex-biased disparities on dispersion and on differential introgression susceptibility of mitochondrial versus nuclear alleles, resulting in mitochondrial capture^33–35^ and (3) selection, causing trait convergence or diversifying adaptive sites^7,36,37^. The discordance between trees recovered from mitogenomes and nuclear genes was already reported for other animal groups. For example, Li et al.^38^ recovered different topologies using mtDNA and nuDNA markers in felid phylogeny and focused on the second hypotheses to explain that difference, in which historical admixture and mitochondrial capture may have occurred between cat ancestor’s lineages which had their divergences around 11 million years ago (MYA)^38,39^. Here we found widespread signature of purifying selection across chelonian mitogenomes, consistent with the previous statement that purifying selection acts constraining the mitogenome evolution to conserve OXPHOS proteins functionality^40^ but we also found evidence for positive selection on OXPHOS genes, revealed by codon-based test when comparing sea turtle lineages with other chelonian lineages. Moreover, some OXPHOS genes seem to be under intensified selection based on RELAX results (K > 1) and codeML results (ω higher in relation to other genes due to less conservative evolutionary constraints), suggesting that in sea turtles, these genes experienced an acceleration on their evolution and that selection may be a factor that could explain discordance among mitochondrial and nuclear gene trees. However, we cannot rule out possible ancient introgression and incomplete lineage sorting (ILS) as plausible sources for this divergence. Although hybridization and ILS effects are known to be stronger in the case of groups with considerably recent divergence times^33^, microevolutionary processes can also impact deep divergences ^41^ as the divergence of the ancestral lineage of sea turtles from their non-marine sister group^41^, which dates around 66 MYA (± 30 MYA, 95% HPD) ^30,31,42^. Moreover, ancient mitochondrial capture has already been suggested as an explanation for conflicting topologies of the well supported nuclear and mitochondrial trees for other groups of turtles^43–45^ and the use of historical type specimens sequencing can be effective to highlight this issue for sea turtles.

PAML analysis showed higher ω on three OXPHOS genes (*COX2*, *CYTB*, and *ND1*) specifically for the sea turtles clade over other chelonian species, although there is no statistically significant evidence for positive selection acting on a greater proportion of these genes. Also, our analysis showed a faster rate of evolution for all turtles to the ATP8 gene, when compared to the other genes, evidenced by the analysis of genetic diversity and the high estimated ω values. PAML results, together with the selection intensification signals indicated by the RELAX tests, suggest that marine lineages have accumulated a greater number of non-synonymous amino acid changes than the other testudines lineages on these genes.

The use of distinct methods and assumptions allowed us to better explore patterns of molecular evolution in sea turtles. Taken together, all selection tests were able to identify 5 sites evolving under positive selection in four genes (*ATP6*, *ND4*, *ND4L* and *ND5*) (Tables 2, 3). Moreover, aBSREL analysis found evidence of episodic diversifying selection on 4.2% of sites on the ancestral branch of the Chelonioidea clade. However, only the strongly supported sites, identified by more than one selection test, were considered under actual positive selection. These sites are 169 and 181 from *ND4* gene and 3 from *ND5* gene and were detected on lineages within the Chelonioidea clade. One exception worth to be mentioned is the 36th site of *ND4* gene, which was identified to be evolving under positive selection by FITMODEL and the only positively selected site with amino acid substitution shared by all sea turtles species. Also, this site is located within a functional region based on the domain organization of the *ND4* subunit of NADH dehydrogenase (ubiquinone), suggesting a physiological impact on this gene in sea turtles.

The three genes that showed evidence of accelerated evolution encode subunits of three separate complexes of the electron transport chain (*COX2*—complex IV, *CYTB*—complex III and *ND1*—complex I). This result reveals that these genes have an excess of non-synonymous replacements than expected for the sea turtle lineage, differentiating the evolution rate of these genes for the sea turtle lineage from the rest of the turtles. Many studies have investigated the role of selection on mitochondrial genes in organisms that inhabit high altitudes^6,10,14–16^, but few focused on organisms with a more active lifestyle. For instance, studies comparing flying versus non-flying species in groups of bats^46^ and grasshoppers^13^ found seven positively selected genes (*ATP8*, *COX3*, *ND2*, *ND4*, *ND4L*, *ND5*, and *ND6*) related to flight in grasshoppers, while *ND2*, *ND3*, *ND4L*, *ND4*, *ND5*, *ND6*, and *COX2* genes were found to have higher values of ω in bats^46^. Our results, combined with these studies, suggest that complex I and IV genes may be important candidates to be impacted by the action of positive selection related to a more active lifestyle. On the other hand, as with other studies, *COX1* and *COX3* genes showed the lowest ω values and highest signs of purifying selection^7,47^.

TreeSaap analysis of these genes indicated that regions with non-synonymous substitutions correspond to radical amino acid changes, although not showing significant positive selection signals. These radical changes occur when the altered residue does not share similar physicochemical properties with the ancestral residue indicating significant functional impact on the protein^48^. Several studies correlated variation in the properties of amino acids at mitogenomic coding regions in several species with (a) more specialized metabolic requirements, such as elephants and their large body size^49^, dugong, sloth, and pangolin, and their low energy diet^50^, (b) an increased tolerance in thermal range in cetaceans and penguins^11,17^, (c) flying in bats^46^, and (d) living at high altitudes in galliniform birds^16^ and alpine pheasants^15^. Compared to these studies, the mitogenome of sea turtles showed less evidence of the pervasive action of natural selection. This pattern was expected because, despite the deep divergence among marine species (at least 30 MYA)^29^, previous studies support a slower evolutionary ratio and a slower molecular clock for the chelonian mitogenome^51,52^. Moreover, sea turtles, as ectothermic reptiles, are considered to have a low metabolic rate, using their energy reserves more slowly than endothermic species^53^. Also, these poor thermogenic abilities generally confine sea turtles, except for *D. coriacea*, to shallow tropical waters^53^, reducing the selective pressure of temperature change, one of the main hypotheses used to explain their patterns of mitochondrial genome evolution^11^. These characteristics may explain the fewer adaptive molecular footprints in this chelonian system compared to the numerous molecular changes already described for endothermic organisms.

OXPHOS genes comprise numerous subunits that are encoded by both the mitogenome and the nuclear genome^54,55^. Hence, research considering their nuclear components can contribute to fully understand the evolution of OXPHOS genes in sea turtles^56–59^. In this scenario, investigating all OXPHOS system in turtles is important mainly due to the high incidence of hybridization among sea turtle species^60–67^. Compatibility between the nuclear and mitochondrial components of the OXPHOS system is of utmost importance for metabolic and energetic optimal fitness^54,55^, and the impact of this phenomenon could be investigated on hybrid turtles. Moreover, approaches integrating population genetic studies with biochemical and physiological experiments can represent a next step^57^ to understand the evolution of the OXPHOS system in these organisms.

## Conclusion

In summary, here we investigated evolutionary patterns and footprints of selection in sea turtles OXPHOS genes under the hypothesis that the more active lifestyle of sea turtles could be exerting greater selective pressure on these genes. We found evidence for positive selection at the coding level for several sites in *ND4* and *ND5* genes for different sea turtles species, highlighting a site within a functional domain of the *ND4* gene with selection signal shared by all species of the Chelonioidea clade. Although the active lifestyle of sea turtles does not seem to exert strong selective pressure on the mitochondrial genes of the OXPHOS system, the few genes with higher ω values compared with other chelonians and the greater fixation of non-synonymous mutations in these genes found for the sea turtle lineage may be responsible for the incongruencies between mitochondrial or nuclear marker inferred topologies. Our results emphasize the importance of using different analyses when assessing selection at the mitogenome level. Also, our study provides first insights into the adaptive evolution of the mtDNA genome in sea turtles, which may have facilitated the successful radiation and diversification of turtle species into the marine environment.

## Material and methods

### Dataset

We retrieved the whole mtDNA genome sequences of 90 turtles from NCBI database, comprising 13 of the 14 Testudines families (Supplementary Table S1 online). Only the Dermatemyididae family was not included in our analyses due to the absence of *Dermatemys mawii* mitogenome on public databases, the only living species in this family. We also retrieved 20 reptile mitogenomes encompassing Squamata, Aves, and Crocodylia orders to be used as outgroups (Supplementary Table S1 online). We generated multiple sequence alignments using MAFFT version 7^68,69^. Nucleotide sequences were first aligned, translated into amino acids, aligned again, then converted into a codon alignment, using PAL2NAL tool^70^ and manually inspected. We manually edited these sequences to preserve the expected reading frame (0-frame) prior to alignment, as some species with frameshifting insertions and/or deletions (indels) of 1 or 2 bp in *ND3* gene were observed by^71^. Due to an extensive translocation of the gene cluster trnH/trnS1/trnL1/nad5 on *Platysternon megacephalum*^72^ and the usual overlapping sites between *ATP8* and *ATP6* genes found in turtles mitogenome^19^, we extracted individual genes and non-coding regions for all species, based on the genome annotations in GenBank and on sequence alignments to keep the correct genes size and correctly infer substitution models. All sequences for *ND6* were reverse complemented due to their encoding by the reverse strand of the mitogenome. These regions were aligned individually and then concatenated, resulting in 39 partitions. Four different datasets were used in different analytical steps: (I) complete mitogenome for 110 species, (II) only protein-coding genes (PCGs) aligned separately and then concatenated for 110 species, (III) PCGs aligned individually for 110 species, and (IV) PCGs aligned individually only for the seven marine species.

To explore the patterns of diversity on mtDNA protein-coding genes for sea turtles we estimated the number of polymorphic sites (S) and nucleotide diversity (π) using the DNAsp v. 6.12.03^73^ for all 13 mitochondrial PCGs.

### Phylogenetic reconstruction

We used IQ-TREE v. 1.6.8 software^74^ to reconstruct maximum likelihood (ML) trees for all datasets, with 1000 ultrafast bootstrap replicates to assess nodal support^75^. Only nodes with support values ≥ 80 were considered robust. We also estimated Bayesian trees for datasets I and II using MRBAYES v. 3.2.6^76^, applying the partitioned models estimated with PARTITION FINDER v. 2.1.1^77^ (Supplementary Table S2 online), according to the Bayesian Information Criterion (BIC). Markov chain Monte Carlo (MCMC) was run for 5,000,000 generations with four chains, and trees were sampled every 100 generations. The convergence of parameters was assessed using TRACER v. 1.7.1^78^, after excluding an initial 10% for each run. Phylogenetic trees were constructed using dataset I and II to compare the efficiency of the presence of non-coding regions on dataset I in topology resolution on Bayesian and ML approaches for both datasets.

### Selection analyses

In order to test a possible effect of topology on the inference of sites under selection, we also performed the selection analyses using the topology inferred with genomic data. Since there was no difference, only the results with the topology inferred with the mitochondrial data are shown. We performed selection analyses using an alignment of the aligned PCGs for the 110 species (dataset III) and an alignment of the aligned PCGs only for the 7 species of the Chelonioidea clade (dataset IV) alignments after conversion into codon alignments on PAL2NAL program^70^. To explore patterns of natural selection and identify sites targeted by positive selection in each mitochondrial coding gene for sea turtle lineage, we explored variation in the ⍵ ratio (*dN*/*dS*, where *dN* is the non-synonymous substitutions rate and *dS* is the synonymous substitutions), in a Bayesian framework using FUBAR from HyPhy package v. 2.1^79^, a ML framework using codeML program from PAML v. 4.9 h package^80^, FITMODEL v. 0.5.3 software^81^, MEME and RELAX from HyPhy package v. 2.1^82^ and in a joint approach of ML and counting methods in SLAC, also from HyPhy package^83^. See Spielman et al.^84^ for a detailed comparison of HyPhy's methods.

#### Branch model analysis

To test if the ω in sea turtles were different from the rest of the tree we used codeML branch models on dataset III, which allows ω to vary among branches in the phylogeny^85^. codeML groups several different models, that vary in terms of their assumptions about how *ω* varies across branches of the phylogeny (branch models), across the sequence (site models), and across both (branch-site model)^86^. For branch models, we first estimated a unique ω value for all branches along the tree with the one-ratio model. Then, using the free-ratio model, we assumed an independent ω for each branch. Finally, we estimated one ω for sea turtle lineage and another for the rest of the phylogeny applying a two-ratio model. We labeled the superfamily Chelonioidea (lineage of sea turtles) as the foreground branch in each phylogenetic tree generated for each PCG in separate analyses, for two-ratio model and all branch-site models described below. The target clade for these analyses is represented in bold clade in Fig. 1 (Chelonioidea clade). All remaining branches, which include terrestrial and freshwater turtles, were not marked, being considered by the algorithm as background branches. The same branch-label scheme was used in RELAX (dataset III) to infer if selection strength has been intensified (K > 1) or it has been relaxed (K < 1) in the Chelonioidea superfamily.

#### Branch-site models analysis

Branch-site models were used to determine if some proportion of sites is subject to positive selection along Chelonioidea lineage. Therefore, for branch-site analysis on codeML and FITMODEL we also divided the tree inferred from dataset III into foreground branch (Chelonioidea clade), where sites may be evolving under positive selection, and background branches (all remaining lineages or non-marine turtles), where positive selection is absent^80,86,87^. On codeML we used model A versus their null model. FITMODEL is well suited for exploratory analysis and we tested if our data fitted to the nested codon-substitution models M0 and M3^80,86,87^. While model M0 assumes that all sites in a sequence alignment are subject to the same selection process, the M3 model assumes variation in selective constraint across sites and is modeled as three rate ratio classes with ω_1_, ω_2_, and ω_3_. FITMODEL allows site-specific switches between different values of the nonsynonymous/synonymous rate ratio^81^ and we tested the switching models M3 + S1 and M3 + S2. Under the Switching test on FITMODEL, a time-reversible Markov process with three additional parameters is modeled: the overall rate of interchange among rate ratio classes (δ), a coefficient for shifts between ω_1_ and ω_3_ (α), and a coefficient for shifts between ω_2_ and ω_3_ (β). The S1 model imposes equal switching rates among ω_1_, ω_2_ and ω_3_ rate ratio classes (α = β = 1), while the S2 model allows α and β to vary freely accounting for unequal rates of switches between selection classes^81^. Finally, we applied a third branch-site test using aBSREL (“adaptive branch-site random effects likelihood”) from HyPhy^88^ using dataset III. Different from codeML and FITMODEL, rates are calculated for every branch in aBSREL, allowing positive selection on background branches. To avoid overparameterization aBSREL infers, using small-sample Akaike Information Criterion correction (AICc), the optimal number of rate categories per branch, rather than assuming that each branch should be fitted with three rate classes. Also, *p* values obtained from individual tests for multiple comparisons were corrected by aBSREL using the Bonferroni–Holm procedure to control family-wise false-positive rates^84,88^.

#### Site-models

We also applied site-model analysis inside the Chelonioidea phylogeny using dataset IV. We used Single-Likelihood Ancestor Counting (SLAC)^89^ to get an overview of the 13 PCGs selection signatures for sea turtles. SLAC infer *dN* and *dS* rates on a per-site basis using a combination of ML and counting approaches. We used a mixed-effects model of evolution (MEME)^90^ to detect sites evolving under positive selection inside the Chelonioidea branch. Similar to FITMODEL, MEME applies a branch-site random effects phylogenetic framework allowing the distribution of ω to vary from site to site as well as from branch to branch, which allows MEME to identify instances of both episodic and pervasive positive selection. We also used FUBAR software to estimate the number of nonsynonymous and synonymous substitutions at each codon on 13 PCGs for sea turtles phylogeny, providing the posterior probability of every codon belonging to a set of classes of ω (ω = 1, ω < 1 or ω > 1)^91^. Significance was assessed by posterior probability (PP) > 0.95.

For all selection analyses based on ML, nested models were compared using the likelihood ratio test (LRT) and their results were evaluated against ${\chi }^{2}$ distributions with different degrees of freedom according to each test. CodeML’s branch-site and RELAX use a ${\chi }_{1}^{2}$ distribution, MEME uses a 0.33:0.3:0.37 mixture of ${\chi }_{0}^{2}$, ${\chi }_{1}^{2}$, and ${\chi }_{2}^{2}$, while FITMODEL uses a mixture of 0.5:0.5 and ${\chi }_{2}^{2}$ distributions for M0 × M3, M3 × M3 + S1 and M3 + S1 × M3 + S2 comparisons, respectively. The significance for all analyses was established at *p* < 0.05.

Genes detected with higher ω values in codeML tests were then analyzed in TreeSAAP v. 3.2^92^ inside the Chelonioidea phylogeny (dataset IV). TreeSAAP relies on the MM01 model implemented in baseML from PAML package^85^ and uses a phylogeny to reconstruct the most likely ancestral states for the gene sequences under investigation. This software assigns weight values to the nonsynonymous codon changes, for which overall physicochemical effects are assessed using a model with 31 physicochemical amino acid properties. The magnitude of the change is rated from 1 (most conservative) to 8 (most radical). A significant deviation from neutral evolution is tested via a z-score and interpreted as a result of positive selection. A highly significant z-score calculated in TreeSAAP (z > 3.09, *p* < 0.01) indicates more non-synonymous substitutions than assumed under the neutral model^92^. To ensure conservative calling of positively selected codon sites only amino acid changes with a score between 6 and 8 and with a positive z-score < 0.001 were used^92^. Finally, we employed functional analysis of PCGs using InterPro 76.0 web resource^93^, to predict protein domains and investigate whether sites identified under positive selection are present within or near functional regions.

## Supplementary information

Supplementary information.
