# Supplementary material for: The role of selection in the evolution of marine turtles mitogenomes
Source: Sci Rep. 2020 Oct 12;10:16953. doi: 10.1038/s41598-020-73874-8 (PMC7550602; doi:10.1038/s41598-020-73874-8)
Supplement: Supplementary file 1 — Supplementary information. [file 41598_2020_73874_MOESM1_ESM.docx]

**SUPPLEMENTARY MATERIAL**

**The role of selection in the evolution of marine turtles mitogenomes**

Elisa Karen da Silva Ramos, Lucas Freitas and Mariana F. Nery^*^

Laboratório de Genômica Evolutiva. Departamento de Genética. Evolução. Microbiologia e Imunologia. Universidade Estadual de Campinas. Brazil.

Elisa K. S. Ramos: elisaksramos@gmail.com

Lucas Freitas: freitas.la@protonmail.ch

Mariana F. Nery: marinery@unicamp.br

*Correspondence to Mariana F. Nery: marinery@unicamp.br

**Address: Laboratório de Genômica Evolutiva. Departamento de Genética. Evolução. Microbiologia e Imunologia. Universidade Estadual de Campinas. Cidade Universitária. 13083970 - Campinas. SP – Brazil**


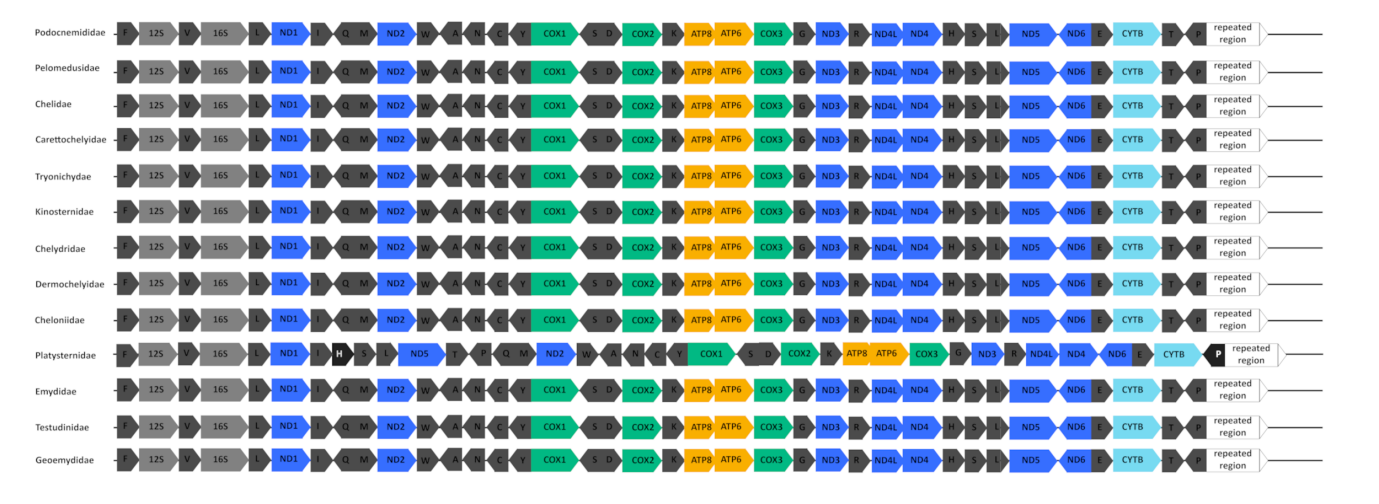


**Figure S1.** Mitochondrial genome organization of Testudines families showing tRNAs (grey), Ribosomal RNA (dark grey), protein coding genes from complex I (dark blue), complex III (light blue), complex IV (green) and complex V (yellow). Arrows ṕointed to right represent regions transcribed from the heavy strand, while arrows pointed to left represent regions transcribed from the light strand. The major strand encodes the majority of protein-coding genes with exception of *ND6*.

**
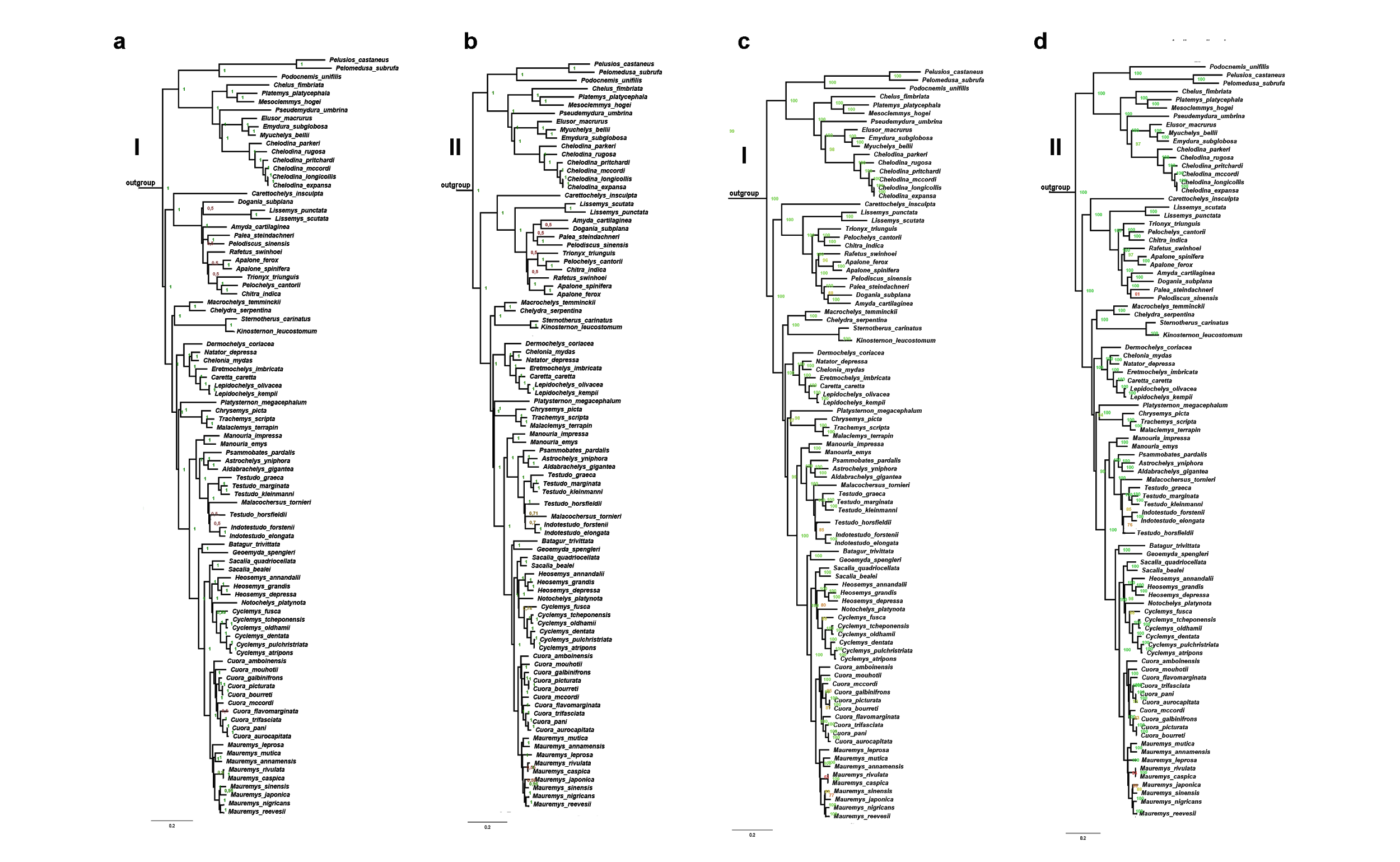
**

**Figure S2.** mtDNA trees generated in our study. **a.** Bayesian tree using dataset I. **b.** Bayesian tree using dataset II. **c.** ML tree using dataset I and **d.** ML tree using dataset II. Numbers under nodes for **a** and **b** are posterior probabilities and numbers under nodes on **c** and **d** represent bootstrap support.


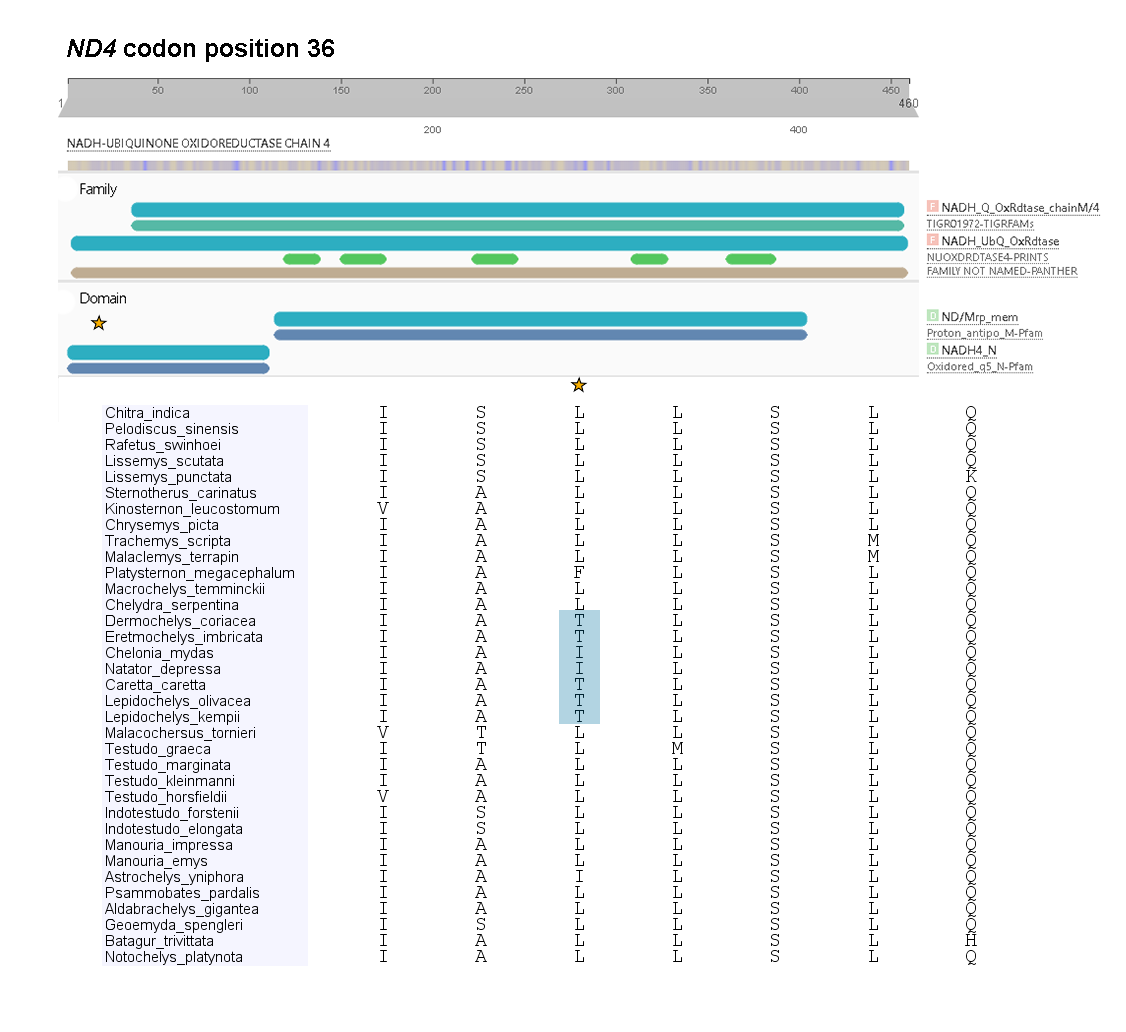


**Figure S3.** Protein domains and alignments showing the variants highlighted in the Chelonioidea clade for the 36th codon position of *ND4* gene. Stars represent the position with positive selection signs.


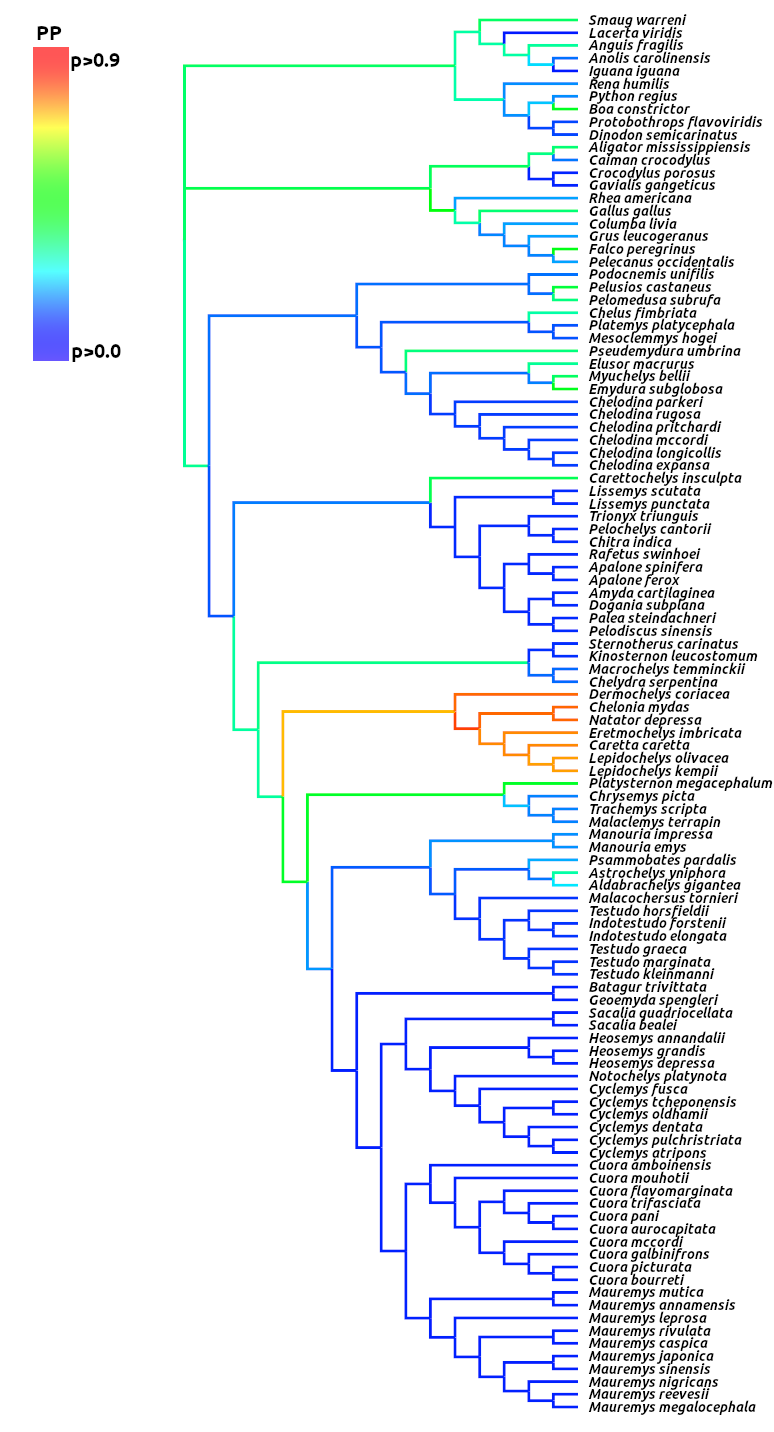


**Figure S4.** Selection shifts at the 36th codon position of *ND4* gene showing positive selection on the Chelonioidea clade. Branches under relaxed selection are shown in red (PP greater than 90% for placing a branch in the ω3 selection class). Branches under strong purifying selection are shown in blue (PP lower than 20%). Branches shown in the rest colors are under moderate purifying selection (PP from 21 to 89%).

| **Table S1.** Species used in this study and the respective accession numbers from NCBI. | | | |
| --- | --- | --- | --- |
| **Order** | **Species** | **Common name** | **CBI accession number** |
| Testudines | *Aldabrachelys gigantea* | Aldabra giant tortoise | NC_028438.1 |
|  | *Amyda cartilaginea* | Asiatic softshell turtle | KY100866.1 |
|  | *Apalone ferox* | Florida softshell turtle | NC_014054.1 |
|  | *Apalone spinifera* | Spiny softshell turtle | NC_021371.1 |
|  | *Astrochelys yniphora* | Angonoka tortoise | JX317746.1 |
|  | *Batagur trivittata* | Angonoka tortoise | NC_032300.1 |
|  | *Caretta caretta* | Loggerhead sea turtle | FR694649.1 |
|  | *Carettochelys insculpta* | Pig-nosed turtle | NC_014048.1 |
|  | *Chelodina expansa* | Broad-shelled river turtle | KY705230.1 |
|  | *Chelodina longicollis* | Eastern long-necked turtle | NC_024667.1 |
|  | *Chelodina mccordi* | Roti Island snake-necked turtle | KY705231.1 |
|  | *Chelodina parkeri* | Parker's snake-necked turtle | KY705232.1 |
|  | *Chelodina pritchardi* | Pritchard's snake-necked turtle | KY705233.1 |
|  | *Chelodina rugosa* | Northern snake-necked turtle | NC_015986.1 |
|  | *Chelonia mydas* | Green sea turtle | AB012104.1 |
|  | *Chelus fimbriata* | Matamata Turtle | NC_015989.1 |
|  | *Chelydra serpentina* | Common snapping turtle | NC_011198.1 |
|  | *Chitra indica* | Indian narrow-headed softshell turtle | NC_026028.1 |
|  | *Chrysemys picta* | Painted turtle | NC_002073.3 |
|  | *Cuora amboinensis* | Amboina box turtle | NC_014769.1 |
|  | *Cuora aurocapitata* | Yellow-headed box turtle | NC_009509.1 |
|  | *Cuora bourreti* | Bourret's box turtle | NC_017885.1 |
|  | *Cuora flavomarginata* | Chinese box turtle | NC_012054.1 |
|  | *Cuora galbinifrons* | Indochinese box turtle | NC_014102.1 |
|  | *Cuora mccordi* | McCord's box turtle | KU258498.1 |
|  | *Cuora mouhotii* | Keeled box turtle | NC_010973.1 |
|  | *Cuora pani* | Pan's box turtle | NC_014401.1 |
|  | *Cuora picturata* | Southern Vietnamese box turtle | NC_017878.1 |
|  | *Cuora trifasciata* | Golden coin turtle | NC_022857.1 |
|  | *Cyclemys atripons* | Western black-bridged leaf turtle | NC_010970.1 |
|  | *Cyclemys dentata* | Asian leaf turtle | NC_018793.1 |
|  | *Cyclemys fusca* | Myanmar brown leaf turtle | JX218031.1 |
|  | *Cyclemys oldhamii* | Oldham's leaf turtle | NC_023220.1 |
|  | *Cyclemys pulchristriata* | Eastern black-bridged leaf turtle | NC_026027.1 |
|  | *Cyclemys tcheponensis* | Stripeneck leaf turtle | NC_023221.1 |
|  | *Dermochelys coriacea* | Leatherback sea turtle | JX454992.1 |
|  | *Dogania subplana* | Malayan softshell turtle | NC_002780.1 |
|  | *Elusor macrurus* | Mary River turtle | NC_030217.1 |
|  | *Emydura subglobosa* | Red-bellied short-necked turtle | NC_026048.1 |
|  | *Eretmochelys imbricata* | Hawksbill sea turtle | DQ533485.1 |
|  | *Geoemyda spengleri* | Black-breasted leaf turtle | KU641028.1 |
|  | *Heosemys annandalii* | Yellow-headed temple turtle | NC_020668.1 |
|  | *Heosemys depressa* | Arakan forest turtle | NC_026024.1 |
|  | *Heosemys grandis* | Giant Asian pond turtle | NC_032297.1 |
|  | *Indotestudo elongata* | Elongated tortoise | DQ656607.1 |
|  | *Indotestudo forstenii* | Forsten's tortoise | NC_007696.1 |
|  | *Kinosternon leucostomum* | White-lipped mud turtle | NC_014577.1 |
|  | *Lepidochelys kempii* | Kemp's ridley sea turtle | JX454982.1 |
|  | *Lepidochelys olivacea* | Pacific ridley sea turtle | JX454991.1 |
|  | *Lissemys punctata* | Indian flapshell turtle | NC_012414.1 |
|  | *Lissemys scutata* | Burmese flapshell turtle | NC_025494.1 |
|  | *Macrochelys temminckii* | Alligator snapping turtle | NC_009260.1 |
|  | *Malaclemys terrapin* | Diamondback terrapin | NC_031300.1 |
|  | *Malacochersus tornieri* | Pancake tortoise | NC_007700.1 |
|  | *Manouria emys* | Asian forest tortoise | NC_007693.1 |
|  | *Manouria impressa* | Impressed tortoise | NC_011815.1 |
|  | *Mauremys annamensis* | Vietnamese pond turtle | NC_017875.1 |
|  | *Mauremys caspica* | Caspian turtle | NC_027826.1 |
|  | *Mauremys japonica* | Japanese pond turtle | NC_016951.1 |
|  | *Mauremys leprosa* | Spanish pond turtle | NC_031432.1 |
|  | *Mauremys mutica* | Yellow pond turtle | NC_009330.1 |
|  | *Mauremys nigricans* | Red-necked pond turtle | NC_029369.1 |
|  | *Mauremys reevesii* | Chinese pond turtle | KJ700438.1 |
|  | *Mauremys rivulata* | Balkan terrapin | NC_029183.1 |
|  | *Mauremys sinensis* | Chinese stripe-necked turtle | NC_016685.1 |
|  | *Mesoclemmys hogei* | Hoge's Toadhead Turtle | NC_036346.1 |
|  | *Myuchelys bellii* | Namoi River snapping turtle | NC_035748.1 |
|  | *Natator depressa* | Australian flatback sea turtle | NC_018550.1 |
|  | *Notochelys platynota* | Malayan flat-shelled turtle | HQ853256.1 |
|  | *Palea steindachneri* | Wattle-necked softshell turtle | NC_013841.1 |
|  | *Pelochelys cantorii* | Asian giant softshell turtle | JN016747.1 |
|  | *Pelodiscus sinensis* | Chinese softshell turtle | NC_006132.1 |
|  | *Pelomedusa subrufa* | African helmeted turtle | NC_001947.1 |
|  | *Pelusios castaneus* | West African mud turtle | NC_026049.1 |
|  | *Platemys platycephala* | Twist-necked turtle | NC_026050.1 |
|  | *Platysternon megacephalum* | Big-headed turtle | NC_007970.1 |
|  | *Podocnemis unifilis* | Yellow-spotted River Turtle | NC_018865.1 |
|  | *Psammobates pardalis* | Leopard tortoise | NC_007694.1 |
|  | *Pseudemydura umbrina* | Western swamp turtle | NC_035731.1 |
|  | *Rafetus swinhoei* | Yangtze giant softshell turtle | NC_017901.1 |
|  | *Sacalia bealei* | Beale's eyed turtle | NC_016691.1 |
|  | *Sacalia quadriocellata* | Four-eyed turtle | GU320209.1 |
|  | *Sternotherus carinatus* | Razor-back musk turtles | NC_017607.1 |
|  | *Testudo graeca* | Greek tortoise | NC_007692.1 |
|  | *Testudo horsfieldii* | Russian tortoise | NC_007697.1 |
|  | *Testudo kleinmanni* | Kleinmann's tortoise | NC_007699.1 |
|  | *Testudo marginata* | Marginated tortoise | NC_007698.1 |
|  | *Trachemys scripta* | Pond Slider | NC_011573.1 |
|  | *Trionyx triunguis* | African softshell turtle | NC_012833.1 |
| **Outgroups** |  |  |  |
| Aves | *Columba livia* | Rock dove | GU908131.1 |
|  | *Falco peregrinus* | Peregrine falcon | JQ282801.1 |
|  | *Gallus gallus* | Red junglefowl | AP003580.1 |
|  | *Grus leucogeranus* | Siberian White Crane | MH041490.1 |
|  | *Pelecanus occidentalis* | Brown Pelican | MH041272.1 |
|  | *Rhea americana* | Greater rhea | NC_000846.1 |
| Crocodilya | *Alligator mississippiensis* | American Alligator | Y13113.1 |
|  | *Caiman crocodylus* | Spectacled caiman | AJ404872.2 |
|  | *Crocodylus porosus* | Saltwater crocodile | AJ810453.1 |
|  | *Gavialis gangeticus* | Gharial | AB079596.1 |
| Squamata | *Anguis fragilis* | Common slowworm | EU443256.1 |
|  | *Anolis carolinensis* | Green Anole | EU747728.2 |
|  | *Boa constrictor* | Red-tailed boa | AB177354.1 |
|  | *Dinodon semicarinatus* | Loo-Choo Big-tooth Snake | AB008539.1 |
|  | *Iguana iguana* | Green Iguana | AJ278511.2 |
|  | *Lacerta viridis* | European green lizard | AM176577.1 |
|  | *Protobothrops flavoviridis* | Habu | LC073746.1 |
|  | *Python regius* | Royal Python | AB177878.1 |
|  | *Rena humilis* | Western Threadsnake cahuilae | AB079597.1 |
|  | *Smaug warreni* | Warren's girdled lizard | AB079613.1 |

| **Table S2**. Substitution model for all markers estimated by PartitionFinder 2. ATP synthase subunit 6 (ATP6), ATP synthase subunit 8 (ATP8), Cytochrome b (CYTB), Cytochrome c oxidase. subunit 1 (COI), Cytochrome c oxidase subunit 2 (COII), Cytochrome c oxidase subunit 3 (COIII), ribosomal RNA Large subunit (16S), NADH dehydrogenase subunit 1 (NDH1), NADH dehydrogenase subunit 2 (NADH2),NADH dehydrogenase subunit 3 (NADH3), NADH dehydrogenase subunit 4 (NADH4), NADH dehydrogenase subunit 4L (NADH4L), NADH dehydrogenase. subunit 5 (NADH5). NADH dehydrogenase. subunit 6 (NADH6). ribosomal RNA Small subunit (12S), tRNA-Alanine (tRNA_Ala), tRNA-Arginine (tRNA_Arg), tRNA-Asparagine (tRNA_Asn), tRNA-Aspartic acid (tRNA_Asp), RNA-Cysteine (tRNA_Cys), tRNA-Glutamic acid (tRNA_Glu), tRNA-Glutamine (tRNA_Gln), tRNA-Glycine (tRNA_Gly), tRNA-Histidine (tRNA_His), tRNA-Isoleucine (tRNA_Ile), tRNA-Leucine (tRNA_Leu), tRNA-Leucine (tRNA_Leu2), tRNA-Lysine (tRNA_Lys), tRNA-Methionine (tRNA_Met), tRNA-Phenylalanine (tRNA_Phe), tRNA-Proline (tRNA_Pro), tRNA-Serine (tRNA_Ser), tRNA-Serine2 (tRNA_Ser2), tRNA-Threonine (tRNA_Thr), tRNA-Tryptophan (tRNA_Trp), tRNA-Tyrosine (tRNA_Tyr), tRNA-Valine (tRNA_Val) and steemloop. | | |
| --- | --- | --- |
| **Best Model** | **# sites** | **Partition names** |
| GTR+I+G | 4716 | COIII_2stpos. COIII_1stpos. COII_1stpos. NADH3_3stpos. tRNA_Phe. NADH4_3stpos. tRNA_Met. COII_2stpos. tRNA_Ile. tRNA_Leu2. CYTB_1stpos. CYTB_3stpos |
| GTR+I+G | 3300 | 16S_1stpos. 16S_3stpos. 12S_1stpos. 12S_2stpos. 12S_3stpos. 16S_2stpos. tRNA_Arg. tRNA_Val |
| GTR+I+G | 1600 | NDH2_1stpos. NDH2_3stpos. tRNA_Trp. tRNA_Thr. NADH4L_2stpos. NADH4L_3stpos. NDH2_2stpos. tRNA_Leu |
| GTR+I+G | 3297 | tRNA_Ser2. NDH5_2stpos. NDH5_1stpos. NDH5_3stpos. CYTB_2stpos. NDH1_1stpos |
| GTR+I+G | 2387 | NDH1_3stpos. COI_1stpos. COI_3stpos. COI_2stpos. tRNA_His. NDH1_2stpos |
| GTR+I+G | 1038 | ATPase6_3stpos. ATPase6_1stpos. tRNA_Tyr. tRNA_Gly. tRNA_Gln. tRNA_Asp. NADH3_2stpos. NADH4L_1stpos |
| GTR+G | 741 | tRNA_Ala. tRNA_Asn. tRNA_Pro. tRNA_Lys. tRNA_Ser. tRNA_Cys. tRNA_Glu. steemloop |
| GTR+I+G | 882 | COIII_3stpos. COII_3stpos. ATPase6_2stpos. ATPase8_3stpos. ATPase8_2stpos |
| GTR+I+G | 1155 | NADH3_1stpos. ATPase8_1stpos. NADH4_1stpos. NADH4_2stpos |
| GTR+I+G | 629 | NDH6_3stpos. NDH6_1stpos. NDH6_2stpos |
| \| Table S3. Results of the LRT test of the branch models in the codeML program for chelonian OXPHOS genes. * statistical significant results. \| \| \| \| \| \| \| \| \| \| --- \| --- \| --- \| --- \| --- \| --- \| --- \| --- \| --- \| \| **Marker** \| **Model** \| **Lnl** \| **np** \| **w** \| **Test** \| **LRT** \| **df** \| **P -value** \| \| ATP6 \| One Model \| -27471.293724 \| 219 \| 0.06665 \| - \| - \| - \| - \| \|  \| Free Model \| -27186.248930 \| 435 \| various \| Free x One \| 570.089588 \| 216 \| 0 \| \|  \| Two model \| -27471.291603 \| 220 \| 0.06662(f) 0.06735(b) \| Two x One \| 0.004 \| 1 \| 0.95 \| \| ATP8 \| One Model \| -5982.602490 \| 217 \| 0.18638 \| - \| - \| - \| - \| \|  \| Free Model \| -5854.682081 \| 431 \| various \| Free x One \| 255.840818 \| 214 \| 0.0264* \| \|  \| Two Model \| -5981.206020 \| 218 \| 0.19367(f) 0.11530(b) \| Two x One \| 2.793 \| 1 \| 0.09 \| \| COX1 \| One Model \| -44893.055888 \| 219 \| 0.01590 \| - \| - \| - \| - \| \|  \| Free Model \| -44532.583851 \| 435 \| various \| Free x One \| 720.944074 \| 216 \| 0 \| \|  \| Two Model \| -44892.433488 \| 220 \| 0.01604(f) 0.01287(b) \| Two x One \| 1.245 \| 1 \| 0.26 \| \| COX2 \| One Model \| -20739.324721 \| 219 \| 0.02678 \| - \| - \| - \| - \| \|  \| Free Model \| -20516.422923 \| 435 \| various \| Free x One \| 445.803596 \| 216 \| 0 \| \|  \| Two Model \| -20736.386438 \| 220 \| 0.02746(f) 0.01529(b) \| Two x One \| 5.877 \| 1 \| 0.02* \| \| COX3 \| One Model \| -24919.286539 \| 219 \| 0.02712 \| - \| - \| - \| - \| \|  \| Free Model \| -24750.281627 \| 435 \| various \| Free x One \| 338.009824 \| 216 \| 2.0671e-7* \| \|  \| Two Model \| -24918.893036 \| 220 \| 0.02736(f) 0.02249(b) \| Two x One \| 0.787 \| 1 \| 0.38 \| \| ND1 \| One Model \| -34735.138570 \| 219 \| 0.03841 \| - \| - \| - \| - \| \|  \| Free Model \| -34437.623937 \| 435 \| various \| Free x One \| 595.029266 \| 216 \| 0 \| \|  \| Two Model \| -34732.628613 \| 220 \| 0.03879(f) 0.02686(b) \| Two x One \| 5.020 \| 1 \| 0.03* \| \| NDH2 \| One Model \| -43648.524589 \| 219 \| 0.08264 \| - \| - \| - \| - \| \|  \| Free Model \| -43420.033349 \| 435 \| various \| Free x One \| 456.98248 \| 216 \| 0 \| \|  \| Two Model \| -43648.479078 \| 220 \| 0.08248(f) 0.08572(b) \| Two x One \| 0.091 \| 1 \| 0.76 \| \| NDH3 \| One Model \| -11513.979992 \| 219 \| 0.07197 \| - \| - \| - \| - \| \|  \| Free Model \| -11301.466002 \| 435 \| various \| Free x One \| 425.02798 \| 216 \| 0 \| \|  \| Two Model \| -11513.786528 \| 220 \| 0.07256(f) 0.06186(b) \| Two x One \| 0.387 \| 1 \| 0.53 \| \| NDH4 \| One Model \| -52658.093508 \| 219 \| 0.05865 \| - \| - \| - \| - \| \|  \| Free Model \| -52360.185074 \| 435 \| various \| Free x One \| 595.816868 \| 216 \| 0 \| \|  \| Two Model \| -52657.848226 \| 220 \| 0.05891(f) 0.05402(b) \| Two x One \| 0.491 \| 1 \| 0.48 \| \| NDH4L \| One Model \| -6939.238770 \| 219 \| 0.08450 \| - \| - \| - \| - \| \|  \| Free Model \| -6804.271673 \| 435 \| various \| Free x One \| 269.934194 \| 216 \| 0.0073* \| \|  \| Two Model \| -6938.950172 \| 220 \| 0.085959(f) 0.06443(b) \| Two x One \| 0.577 \| 1 \| 0.45 \| \| NDH5 \| One Model \| -69072.141364 \| 219 \| 0.06731 \| - \| - \| - \| - \| \|  \| Free Model \| -68520.455212 \| 435 \| various \| Free x One \| 1103.372304 \| 216 \| 0 \| \|  \| Two Model \| -69071.064642 \| 220 \| 0.067869(f) 0.05834(b) \| Two x One \| 2.153 \| 1 \| 0.14 \| \| NDH6 \| One Model \| -13666.958118 \| 219 \| 0.07256 \| - \| - \| - \| - \| \|  \| Free Model \| -13452.761589 \| 435 \| various \| Free x One \| 428.393058 \| 216 \| 0 \| \|  \| Two Model \| -13666.518105 \| 220 \| 0.07154(f) 0.08835(b) \| Two x One \| 0.880 \| 1 \| 0.35 \| \| CYTB \| One Model \| -39988.324898 \| 219 \| 0.04174 \| - \| - \| - \| - \| \|  \| Free Model \| -39669.092779 \| 435 \| various \| Free x One \| 638.464238 \| 216 \| 0 \| \|  \| Two Model \| -39985.477613 \| 220 \| 0.04242(f) 0.03005(b) \| Two x One \| 5.695 \| 1 \| 0.02* \|  \| Table S4. RELAX results. \| \| \| \| \| \| --- \| --- \| --- \| --- \| --- \| \| **Marker** \| **K** \| **LRT** \| **p-value** \| **Significant** \| \| ATP6 \| 1.69 \| 1.56 \| 0.21 \| no \| \| ATP8 \| 20.48 \| 3.92 \| 0.05 \| * \| \| COI \| 1.59 \| 7.13 \| 0.01 \| ** \| \| COII \| 2.00 \| 14.67 \| 0.00 \| ** \| \| COIII \| 1.75 \| 3.78 \| 0.01 \| ** \| \| NDH1 \| 1.78 \| -4.58 \| 1.00 \| no \| \| NDH2 \| 1.00 \| 0.00 \| 1.00 \| no \| \| NDH3 \| 1.05 \| 0.05 \| 0.83 \| no \| \| NDH4 \| 1.33 \| 6.26 \| 0.01 \| * \| \| NDH4L \| 1.58 \| 6.57 \| 0.01 \| * \| \| NDH5 \| 2.70 \| 23.42 \| 0.00 \| ** \| \| NDH6 \| 1.74 \| 3.09 \| 0.08 \| no \| \| CYTB \| 2.14 \| 9.14 \| 0.00 \| ** \|  \| Table S5. Results of the LRT test of the branch-site models for the codeML analyses in chelonian OXPHOS genes. Site class 0: purifying selection in all branches. Site class 1: neutral evolution in all branches. Site class 2a: positive selection in foreground branch and under purifying selection in background branch. Site class 2b: positive selection in foreground branch and neutral evolution in background branch. \| \| \| \| \| \| \| \| \| \| \| \| --- \| --- \| --- \| --- \| --- \| --- \| --- \| --- \| --- \| --- \| --- \| \| **Marker** \| **Model** \| **Lnl** \| **Test** \| **LRT** \| **df** \| **P value** \| **Site class proportion** \| \| \| \| \| 0 \| 1 \| 2a \| 2b \| \| ATP6 \| null \| -29719.17 \| - \| - \| - \| - \| - \| - \| - \| - \| \|  \| MA \| -29719.10 \| MA x null \| 0.1345 \| 1 \| 0.7138 \| 0.74544 \| 0.25024 \| 0.00323 \| 0.00108 \| \| ATP8 \| null \| -8782.79 \| - \| - \| - \| - \| - \| - \| - \| - \| \|  \| MA \| -8782.79 \| MA x null \| 0.0000 \| 1 \| 1 \| 0.60249 \| 0.39751 \| 0.00000 \| 0.00000 \| \| COX1 \| null \| -48528.79 \| - \| - \| - \| - \| - \| - \| - \| - \| \|  \| branch site \| -48528.790 \| MA x null \| -0.0001 \| 1 \| 1 \| 0.91432 \| 0.08568 \| 0.00000 \| 0.00000 \| \| COX2 \| null \| -23116.94 \| - \| - \| - \| - \| - \| - \| - \| - \| \|  \| MA \| -23116.94 \| MA x null \| 0.0000 \| 1 \| 0.9974 \| 0.92205 \| 0.07795 \| 0.00000 \| 0.00000 \| \| COX3 \| null \| -26173.47 \| - \| - \| - \| - \| - \| - \| - \| - \| \|  \| MA \| -26173.47 \| MA x null \| 0.0000 \| 1 \| 1 \| 0.91432 0 \| 0.08568 \| 0.00000 \| 0.0000 \| \| ND1 \| null \| -38318.24 \| - \| - \| - \| - \| - \| - \| - \| - \| \|  \| MA \| -38318.24 \| MA x null \| 0.0001 \| 1 \| 0.9914 \| 0.83969 \| 0.16031 \| 0.00000 \| 0.00000 \| \| ND2 \| null \| -47600.76 \| - \| - \| - \| - \| - \| - \| - \| - \| \|  \| MA \| -47600.76 \| MA x null \| 0.0000 \| 1 \| 1 \| 0.79078 \| 0.20922 \| 0.00000 \| 0.00000 \| \| ND3 \| null \| -16070.30 \| - \| - \| - \| - \| - \| - \| - \| - \| \|  \| MA \| -16070.30 \| MA x null \| 0.0000 \| 1 \| 1 \| 0.72800 \| 0.27200 \| 0.00000 \| 0.00000 \| \| ND4 \| null \| -59758.39 \| - \| - \| - \| - \| - \| - \| - \| - \| \|  \| MA \| -59758.36 \| MA x null \| 0.0576 \| 1 \| 0.8102 \| 0.80942 \| 0.18748 \| 0.00252 \| 0.00058 \| \| ND4L \| null \| -13250.18 \| - \| - \| - \| - \| - \| - \| - \| - \| \|  \| MA \| -13250.18 \| MA x null \| 0.0000 \| 1 \| 1 \| 0.88599 \| 0.11401 \| 0.00000 \| 0.00000 \| \| ND5 \| null \| -85345.72 \| - \| - \| - \| - \| - \| - \| - \| - \| \|  \| MA \| -85345.35 \| MA x null \| 0.7413 \| 1 \| 0.3892 \| 0.68574 \| 0.30953 \| 0.00326 \| 0.00147 \| \| ND6 \| null \| -25494.67 \| - \| - \| - \| - \| - \| - \| - \| - \| \|  \| MA \| -25494.67 \| MA x null \| 0.0000 \| 1 \| 1 \| 0.66553 \| 0.33447 \| 0.00000 \| 0.00000 \| \| CYTB \| null \| -44431.14 \| - \| - \| - \| - \| - \| - \| - \| - \| \|  \| MA \| -44431.14 \| MA x null \| 0.0000 \| 1 \| 1 \| 0.79394 \| 0.20606 \| 0.00000 \| 0.00000 \| | | |

| Table S6. Likelihood analyses and p-values for the models of FITMODEL program for mitochondrial protein-coding genes of sea turtles mitogenomes. Likelihood value (lnL). omega value (ω). parameter estimates values (p). switching rates values (R) and P values. | | | | |
| --- | --- | --- | --- | --- |
|  | **M0** | **M3** | **M3+S1** | **M3+S2** |
| **ATP6** | | | | |
| **lnL** | -28597.918707 | -27545.945460 | -27423.335419 | -27341.257914 |
| **ω1 ω2 ω3** | 0.068 | 0.005 0.059 0.192 | 0.001 0.093 0.327 | 0.002 0.023 0.445 |
| **p1 p2 p3** | 1.000 | 0.407 0.325 0.268 | 0.594 0.269 0.136 | 0.492 0.343 0.165 |
| **R12 R13 R23** | - | - | 0.120 0.120 0.120 | 0.050 0.026 1.589 |
| **x2 (models;LRT;P)** | - | (M0 vs. M3; 2103.94; 0.000) | (M3 vs. M3S1; 245.22; 0.000) | (M3S1 vs. M3S2; 164.15; 0.000) |
| **ATP8** | | | | |
| **lnL** | -8425.553880 | -8092.420836 | -8040.543261 | -8028.943857 |
| **ω1 ω2 ω3** | 0.215 | 0.020 0.166 0.578 | 0.000 0.147 0.724 | 0.000 0.083 0.811 |
| **p1 p2 p3** | 1.000 | 0.238 0.436 0.325 | 0.306 0.341 0.352 | 0.227 0.434 0.338 |
| **R12 R13 R23** | - | - | 0.092 0.092 0.092 | 0.022 0.057 0.373 |
| **x2 (models;LRT;P)** | - | (M0 vs. M3; 666.26; 0.000) | (M3 vs. M3S1; 103.75; 0.000) | (M3S1 vs. M3S2; 23.19; 0.000) |
| **COX1** | | | | |
| **lnL** | -47148.657684 | -46014.583266 | -45905.092721 | -45843.370715 |
| **ω1 ω2 ω3** | 0.018 | 0.002 0.029 0.124 | 0.000 0.061 0.304 | 0.001 0.017 0.366 |
| **p1 p2 p3** | 1.000 | 0.660 0.249 0.091 | 0.796 0.161 0.042 | 0.738 0.213 0.049 |
| **R12 R13 R23** | - | - | 0.206 0.206 0.206 | 0.001 0.230 3.550 |
| **x2 (models;LRT;P)** | - | (M0 vs. M3; 2268.14; 0.000) | (M3 vs. M3S1; 218.98; 0.000) | (M3S1 vs. M3S2; 123.44; 0.000) |
| **COX2** | | | | |
| **lnL** | -47150.379034 | -46010.915975 | -45903.108662 | -45845.642451 |
| **ω1 ω2 ω3** | 0.018 | 0.002 0.029 0.124 | 0.000 0.054 0.268 | 0.001 0.017 0.352 |
| **p1 p2 p3** | 1.000 | 0.662 0.246 0.091 | 0.790 0.161 0.049 | 0.734 0.216 0.050 |
| **R12 R13 R23** | - | - | 0.203 0.203 0.203 | 0.003 0.216 3.197 |
| **x2 (models;LRT;P)** | - | (M0 vs. M3; 2278.92; 0.000) | (M3 vs. M3S1; 215.61; 0.000) | (M3S1 vs. M3S2; 114.93; 0.000) |
| **COX3** | | | | |
| **lnL** | -25051.320892 | -24175.455572 | -24078.422440 | -24055.908814 |
| **ω1 ω2 ω3** | 0.025 | 0.003 0.034 0.141 | 0.000 0.039 0.191 | 0.001 0.024 0.258 |
| **p1 p2 p3** | 1.000 | 0.606 0.285 0.109 | 0.691 0.224 0.085 | 0.634 0.287 0.080 |
| **R12 R13 R23** | - | - | 0.101 0.101 0.101 | 0.080 0.001 1.058 |
| **x2 (models;LRT;P)** | - | (M0 vs. M3; 1751.73; 0.000) | (M3 vs. M3S1; 194.06; 0.000) | (M3S1 vs. M3S2; 45.02; 0.000) |
| **CYTB** | | | | |
| **lnL** | -43063.217435 | -41318.822758 | -41070.265659 | -40975.123248 |
| **ω1 ω2 ω3** | 0.044 | 0.005 0.050 0.169 | 0.001 0.071 0.277 | 0.002 0.019 0.514 |
| **p1 p2 p3** | 1.000 | 0.542 0.271 0.186 | 0.659 0.227 0.114 | 0.631 0.277 0.092 |
| **R12 R13 R23** | - | - | 0.146 0.146 0.146 | 0.035 0.142 4.006 |
| **x2 (models;LRT;P)** | - | (M0 vs. M3; 3488.78; 0.000) | (M3 vs. M3S1; 497.11; 0.000) | (M3S1 vs. M3S2; 190.28; 0.000) |
| **ND1** | | | | |
| **lnL** | -37356.184109 | -36089.402935 | -35865.655701 | -35813.408608 |
| **ω1 ω2 ω3** | 0.043 | 0.004 0.044 0.155 | 0.000 0.057 0.283 | 0.001 0.033 0.340 |
| **p1 p2 p3** | 1.000 | 0.470 0.354 0.175 | 0.614 0.2801 0.105 | 0.542 0.349 0.109 |
| **R12 R13 R23** | - | - | 1.88 1.88 1.88 | 0.124 0.001 0.902 |
| **x2 (models;LRT;P)** | - | (M0 vs. M3; 2533.56; 0.000) | (M3 vs. M3S1; 447.49; 0.000) | (M3S1 vs. M3S2; 104.49; 0.000) |
| **ND2** | | | | |
| **lnL** | -46059.074167 | -44696.391846 | -44354.176507 | -44274.131505 |
| **ω1 ω2 ω3** | 0.079 | 0.014 0.074 0.250 | 0.002 0.070 0.369 | 0.004 0.033 0.490 |
| **p1 p2 p3** | 1.000 | 0.360 0.434 0.205 | 0.478 0.342 0.180 | 0.423 0.408 0.169 |
| **R12 R13 R23** | - | - | 0.161 0.161 0.161 | 0.129 0.001 1.198 |
| **x2 (models;LRT;P)** | - | (M0 vs. M3; 2725.36; 0.000) | (M3 vs. M3S1; 684.43; 0.000) | (M3S1 vs. M3S2; 160.09; 0.000) |
| **ND3** | | | | |
| **lnL** | -15704.210272 | -15184.399257 | -15031.897804 | -14973.233838 |
| **ω1 ω2 ω3** | 0.082 | 0.015 0.071 0.230 | 0.687 0.227 0.086 | 0.004 0.174 19.999 |
| **p1 p2 p3** | 1.000 | 0.471 0.270 0.259 | 0.005 0.199 0.914 | 0.722 0.272 0.006 |
| **R12 R13 R23** | - | - | 0.204 0.204 0.204 | 0.053 5.057 19.004 |
| **x2 (models;LRT;P)** | - | (M0 vs. M3; 1039.62; 0.000) | (M3 vs. M3S1; 305.00; 0.000) | (M3S1 vs. M3S2; 117.32; 0.000) |
| **ND4** | | | | |
| **lnL** | -57901.090845 | -55903.621196 | -55561.710448 | -55439.985151 |
| **ω1 ω2 ω3** | 0.060 | 0.007 0.059 0.209 | 0.001 0.077 0.390 | 0.003 0.041 0.622 |
| **p1 p2 p3** | 1.000 | 0.404 0.406 0.190 | 0.570 0.314 0.116 | 0.515 0.384 0.100 |
| **R12 R13 R23** | - | - | 0.157 0.157 0.157 | 0.096 0.001 1.486 |
| **x2 (models;LRT;P)** | - | (M0 vs. M3; 3994.93; 0.000) | (M3 vs. M3S1; 683.82; 0.000) | (M3S1 vs. M3S2; 243.45; 0.000) |
| **ND4L** | | | | |
| **lnL** | -12564.707611 | -12266.267415 | -12222.977952 | -12195.644478 |
| **ω1 ω2 ω3** | 0.068 | 0.015 0.102 0.259 | 0.009 0.117 0.486 | 0.009 0.043 0.544 |
| **p1 p2 p3** | 1.000 | 0.469 0.458 0.073 | 0.545 0.384 0.071 | 0.476 0.423 0.101 |
| **R12 R13 R23** | - | - | 0.086 0.086 0.086 | 0.019 0.054 1.979 |
| **x2 (models;LRT;P)** | - | (M0 vs. M3; 596.88; 0.000) | (M3 vs. M3S1; 86.57; 0.000) | (M3S1 vs. M3S2; 54.66; 0.000) |
| **ND5** | | | | |
| **lnL** | -81624.485899 | -78800.034691 | -78254.523894 | -78161.721619 |
| **ω1 ω2 ω3** | 0.078 | 0.009 0.067 0.207 | 0.001 0.069 0.305 | 0.000 1.000 0.050 |
| **p1 p2 p3** | 1.000 | 0.334 0.387 0.278 | 0.474 0.304 0.222 | 0.488 0.090 0.422 |
| **R12 R13 R23** | - | - | 0.124 0.124 0.124 | 0.209 0.094 3.335 |
| **x2 (models;LRT;P)** | - | (M0 vs. M3; 5648.90; 0.000) | (M3 vs. M3S1; 1091.02; 0.000) | (M3S1 vs. M3S2; 185.60; 0.000) |
| **ND6** | | | | |
| **lnL** | -24546.278468 | -23702.507287 | -23517.931786 | -23474.777941 |
| **ω1 ω2 ω3** | 0.103 | 0.021 0.087 0.287 | 0.010 0.210 0.735 | 0.010 0.021 0.552 |
| **p1 p2 p3** | 1.000 | 0.358 0.350 0.291 | 0.618 0.246 0.137 | 0.427 0.332 0.242 |
| **R12 R13 R23** | - | - | 0.168 0.168 0.168 | 0.045 0.060 1.039 |
| **x2 (models;LRT;P)** | - | 0.000 | 0.000 | 0.000 |

| **Table S7.** MEME results of positive selection analysis on mtDNA genes for the Chelonoidea clade. | | | | | | | | | | |
| --- | --- | --- | --- | --- | --- | --- | --- | --- | --- | --- |
| **Marker** | **Codon** | **Partition** | **α** | **β-** | **p-** | **β+** | **p+** | **LRT** | **p-value** | **# branches under selection** |
| ATP6 | 179 | 1 | 0.00 | 0.00 | 0.88 | 6.75 | 0.12 | 4.57 | 0.05 | 1.00 |
| ATP8 | 47 | 1 | 0.00 | 0.00 | 0.26 | 1.78 | 0.74 | 13.66 | 0.00 | 1.00 |
| COI | - | - | - | - | - | - | - | - | - | - |
| COII | - | - | - | - | - | - | - | - | - | - |
| COIII | - | - | - | - | - | - | - | - | - | - |
| CYTB | - | - | - | - | - | - | - | - | - | - |
| NDH1 | - | - | - | - | - | - | - | - | - | - |
| NDH2 | - | - | - | - | - | - | - | - | - | - |
| NDH3 | - | - | - | - | - | - | - | - | - | - |
| NDH4 | 181 | 1 | 0.00 | 0.00 | 0.89 | 27.13 | 0.11 | 6.19 | 0.02 | 1.00 |
| NDH4L | 58 | 1 | 0.00 | 0.00 | 0.89 | 51.18 | 0.11 | 4.87 | 0.04 | 1.00 |
| NDH5 | 3 | 1 | 0.00 | 0.00 | 0.76 | 20.88 | 0.24 | 7.31 | 0.01 | 0.00 |
| NDH6 | - | - | - | - | - | - | - | - | - | - |

| **Table S8.** FUBAR results. Significance (pp) >0.95. PS. Positively selected códons; NS. Negatively selected codons | | | | |
| --- | --- | --- | --- | --- |
| **Gene** | **total aa** | **PS** | **NS** | **% PS** |
| ATP6 | 227 | 0 | 63 | 27.75 |
| ATP8 | 62 | 0 | 11 | 17.74 |
| COI | 516 | 0 | 170 | 32.95 |
| COII | 228 | 0 | 68 | 29.82 |
| COIII | 261 | 0 | 67 | 25.67 |
| CYTB | 381 | 0 | 145 | 38.06 |
| ND1 | 323 | 0 | 106 | 32.82 |
| ND2 | 347 | 0 | 78 | 22.48 |
| ND3 | 116 | 0 | 28 | 24.14 |
| ND4 | 460 | 1 | 141 | 30.65 |
| ND4L | 98 | 0 | 26 | 26.53 |
| ND5 | 602 | 1 (3) 0.92 | 258 | 42.86 |
| ND6 | 174 | 0 | 19 | 10.92 |

**Data 1** – *dn*, *ds* and w ratios information of every mitochondrial protein-coding gene estimated with free-model on codeML.

**ATP6**

**dS tree:**

((((((Anolis_carolinensis: 48.785804, Iguana_iguana: 4.222695): 0.000081, Anguis_fragilis: 6.870403): 0.000027, Lacerta_viridis: 5.364556): 0.000045, Smaug_warreni: 10.873468): 0.000048, (Rena_humilis: 11.916649, ((Python_regius: 3.106181, Boa_constrictor: 48.828804): 0.000063, (Protobothrops_flavoviridis: 3.494481, Dinodon_semicarinatus: 8.856808): 0.000057): 0.000144): 0.000267): 0.000029, ((Rhea_americana: 0.870275, (Gallus_gallus: 1.521945, (Columba_livia: 1.133359, (Grus_leucogeranus: 0.931575, (Falco_peregrinus: 0.761858, Pelecanus_occidentalis: 0.864169): 0.000026): 0.079873): 0.000021): 0.913421): 0.515952, ((Alligator_mississippiensis: 0.614521, Caiman_crocodylus: 0.953060): 0.001266, (Crocodylus_porosus: 4.874709, Gavialis_gangeticus: 3.571572): 0.000103): 2.050164): 0.000004, (((Podocnemis_unifilis: 6.271801, (Pelusios_castaneus: 0.000078, Pelomedusa_subrufa: 1.230495): 5.313071): 0.000081, ((Chelus_fimbriata: 1.871601, (Platemys_platycephala: 0.522635, Mesoclemmys_hogei: 0.827743): 0.879228): 1.020428, (((Elusor_macrurus: 0.343466, (Myuchelys_bellii: 0.121292, Emydura_subglobosa: 0.279613): 0.041696): 0.411935, (Chelodina_parkeri: 0.533037, (Chelodina_rugosa: 0.278442, (Chelodina_pritchardi: 0.144019, (Chelodina_mccordi: 0.026672, (Chelodina_longicollis: 0.000002, Chelodina_expansa: 0.000002): 0.023121): 0.186778): 0.000004): 0.095680): 0.550153): 0.691536, Pseudemydura_umbrina: 0.327554): 0.000011): 5.303561): 0.000068, ((Carettochelys_insculpta: 6.803449, ((Lissemys_scutata: 2.732322, Lissemys_punctata: 0.048263): 0.139543, ((Trionyx_triunguis: 0.717064, (Pelochelys_cantorii: 0.461812, Chitra_indica: 0.828925): 0.213010): 0.870697, ((Rafetus_swinhoei: 0.274139, (Apalone_spinifera: 0.128507, Apalone_ferox: 0.180263): 0.254094): 0.566609, ((Amyda_cartilaginea: 0.754943, Dogania_subplana: 0.062825): 0.045030, (Palea_steindachneri: 0.911919, Pelodiscus_sinensis: 0.776138): 0.104627): 0.000008): 0.000012): 0.000026): 3.630004): 0.000029, (((Sternotherus_carinatus: 0.103799, Kinosternon_leucostomum: 0.399498): 1.194594, (Macrochelys_temminckii: 0.316570, Chelydra_serpentina: 0.139246): 0.376386): 0.000023, (((Platysternon_megacephalum: 0.727325, (Chrysemys_picta: 0.094975, (Trachemys_scripta: 0.044534, Malaclemys_terrapin: 0.068702): 0.237926): 0.642133): 0.151178, (((((Astrochelys_yniphora: 0.242567, Aldabrachelys_gigantea: 0.220207): 0.110544, Psammobates_pardalis: 0.370937): 0.059450, ((((Indotestudo_forstenii: 0.035416, Indotestudo_elongata: 0.077859): 0.133828, Testudo_horsfieldii: 0.213946): 0.017796, (Testudo_graeca: 0.226376, (Testudo_marginata: 0.104108, Testudo_kleinmanni: 0.101486): 0.000005): 0.085709): 0.063276, Malacochersus_tornieri: 0.260285): 0.075287): 0.000012, (Manouria_impressa: 0.218085, Manouria_emys: 0.266732): 0.277097): 0.082634, ((Batagur_trivittata: 0.623332, Geoemyda_spengleri: 0.491661): 0.036769, ((((Notochelys_platynota: 0.202833, (Cyclemys_fusca: 0.047874, ((Cyclemys_tcheponensis: 0.000004, Cyclemys_oldhamii: 0.018395): 0.103054, (Cyclemys_dentata: 0.040024, (Cyclemys_pulchristriata: 0.000004, Cyclemys_atripons: 0.008505): 0.056376): 0.192373): 0.096256): 0.051618): 0.085045, (Heosemys_annandalii: 0.378998, (Heosemys_grandis: 0.298044, Heosemys_depressa: 0.254622): 0.000001): 0.026836): 0.000004, (Sacalia_quadriocellata: 0.229211, Sacalia_bealei: 0.125067): 0.191922): 0.062087, (((((Cuora_flavomarginata: 0.128434, (Cuora_trifasciata: 0.023464, (Cuora_pani: 0.000000, Cuora_aurocapitata: 0.000000): 0.037285): 0.029333): 0.000004, (Cuora_mccordi: 0.048372, (Cuora_galbinifrons: 0.046684, (Cuora_picturata: 0.094979, Cuora_bourreti: 0.031748): 0.014218): 0.064268): 0.003141): 0.047787, Cuora_mouhotii: 0.033057): 0.084341, Cuora_amboinensis: 0.269424): 0.029317, ((Mauremys_leprosa: 0.107623, (((Mauremys_japonica: 0.041606, Mauremys_sinensis: 0.063622): 0.014268, ((Mauremys_reevesii: 0.000005, NC_015101.1: 0.004525): 0.046176, Mauremys_nigricans: 0.053388): 0.006984): 0.046531, (Mauremys_rivulata: 0.000000, Mauremys_caspica: 0.000000): 0.111336): 0.025537): 0.012449, (Mauremys_mutica: 0.088283, Mauremys_annamensis: 0.067483): 0.087820): 0.060204): 0.046069): 0.240795): 0.133524): 0.302385): 0.120475, (Dermochelys_coriacea: 0.311169, ((Eretmochelys_imbricata: 0.190846, (Caretta_caretta: 0.056930, (Lepidochelys_olivacea: 0.024142, Lepidochelys_kempii: 0.026872): 0.125858): 0.058183): 0.065276, (Chelonia_mydas: 0.264596, Natator_depressa: 0.222497): 0.119533): 0.153898): 0.452117): 0.000004): 0.000055): 10.630284): 0.000062);

**dN tree:**

((((((Anolis_carolinensis: 0.110795, Iguana_iguana: 0.038765): 0.043902, Anguis_fragilis: 0.135353): 0.020212, Lacerta_viridis: 0.187060): 0.044592, Smaug_warreni: 0.186236): 0.048394, (Rena_humilis: 0.142679, ((Python_regius: 0.066155, Boa_constrictor: 0.088631): 0.041522, (Protobothrops_flavoviridis: 0.094703, Dinodon_semicarinatus: 0.093755): 0.036488): 0.143532): 0.266346): 0.028224, ((Rhea_americana: 0.025689, (Gallus_gallus: 0.054301, (Columba_livia: 0.029529, (Grus_leucogeranus: 0.026936, (Falco_peregrinus: 0.063225, Pelecanus_occidentalis: 0.013059): 0.017048): 0.002486): 0.011116): 0.018987): 0.079976, ((Alligator_mississippiensis: 0.054888, Caiman_crocodylus: 0.061166): 0.073694, (Crocodylus_porosus: 0.045268, Gavialis_gangeticus: 0.047913): 0.070226): 0.121395): 0.000000, (((Podocnemis_unifilis: 0.090920, (Pelusios_castaneus: 0.053467, Pelomedusa_subrufa: 0.040426): 0.039561): 0.063686, ((Chelus_fimbriata: 0.077920, (Platemys_platycephala: 0.027106, Mesoclemmys_hogei: 0.019144): 0.025063): 0.023038, (((Elusor_macrurus: 0.006277, (Myuchelys_bellii: 0.002405, Emydura_subglobosa: 0.017849): 0.005058): 0.032162, (Chelodina_parkeri: 0.022283, (Chelodina_rugosa: 0.011096, (Chelodina_pritchardi: 0.002429, (Chelodina_mccordi: 0.002216, (Chelodina_longicollis: 0.002176, Chelodina_expansa: 0.002174): 0.002137): 0.017656): 0.000000): 0.014927): 0.036057): 0.016205, Pseudemydura_umbrina: 0.078168): 0.000656): 0.031795): 0.067741, ((Carettochelys_insculpta: 0.077059, ((Lissemys_scutata: 0.065718, Lissemys_punctata: 0.048122): 0.012582, ((Trionyx_triunguis: 0.032651, (Pelochelys_cantorii: 0.012654, Chitra_indica: 0.022850): 0.014282): 0.006978, ((Rafetus_swinhoei: 0.021485, (Apalone_spinifera: 0.004435, Apalone_ferox: 0.009121): 0.025835): 0.007143, ((Amyda_cartilaginea: 0.049239, Dogania_subplana: 0.050043): 0.013549, (Palea_steindachneri: 0.031804, Pelodiscus_sinensis: 0.044071): 0.000010): 0.005234): 0.005172): 0.019813): 0.045962): 0.008459, (((Sternotherus_carinatus: 0.029540, Kinosternon_leucostomum: 0.052449): 0.112897, (Macrochelys_temminckii: 0.016035, Chelydra_serpentina: 0.032889): 0.024894): 0.018852, (((Platysternon_megacephalum: 0.052030, (Chrysemys_picta: 0.008184, (Trachemys_scripta: 0.014502, Malaclemys_terrapin: 0.007057): 0.016929): 0.032629): 0.029906, (((((Astrochelys_yniphora: 0.025107, Aldabrachelys_gigantea: 0.025731): 0.000011, Psammobates_pardalis: 0.025945): 0.003734, ((((Indotestudo_forstenii: 0.016816, Indotestudo_elongata: 0.011666): 0.023785, Testudo_horsfieldii: 0.020955): 0.000002, (Testudo_graeca: 0.015922, (Testudo_marginata: 0.008134, Testudo_kleinmanni: 0.008314): 0.004655): 0.012764): 0.008388, Malacochersus_tornieri: 0.044420): 0.033574): 0.007585, (Manouria_impressa: 0.025060, Manouria_emys: 0.050504): 0.012477): 0.001355, ((Batagur_trivittata: 0.065682, Geoemyda_spengleri: 0.018314): 0.006353, ((((Notochelys_platynota: 0.038510, (Cyclemys_fusca: 0.004837, ((Cyclemys_tcheponensis: 0.000000, Cyclemys_oldhamii: 0.000002): 0.014577, (Cyclemys_dentata: 0.017205, (Cyclemys_pulchristriata: 0.000000, Cyclemys_atripons: 0.000001): 0.004832): 0.027536): 0.007204): 0.014248): 0.000009, (Heosemys_annandalii: 0.039763, (Heosemys_grandis: 0.035756, Heosemys_depressa: 0.048588): 0.000147): 0.005728): 0.003003, (Sacalia_quadriocellata: 0.009758, Sacalia_bealei: 0.014751): 0.006808): 0.002583, (((((Cuora_flavomarginata: 0.014531, (Cuora_trifasciata: 0.009689, (Cuora_pani: 0.000002, Cuora_aurocapitata: 0.000002): 0.002404): 0.007237): 0.000000, (Cuora_mccordi: 0.000005, (Cuora_galbinifrons: 0.007172, (Cuora_picturata: 0.012129, Cuora_bourreti: 0.007317): 0.002336): 0.000006): 0.000000): 0.002285, Cuora_mouhotii: 0.012276): 0.007188, Cuora_amboinensis: 0.009489): 0.000003, ((Mauremys_leprosa: 0.009868, (((Mauremys_japonica: 0.006912, Mauremys_sinensis: 0.009697): 0.000001, ((Mauremys_reevesii: 0.004645): 0.007091, Mauremys_nigricans: 0.009623): 0.002403): 0.002707, (Mauremys_rivulata: 0.000002, Mauremys_caspica: 0.000002): 0.013694): 0.003862): 0.007366, (Mauremys_mutica: 0.019044, Mauremys_annamensis: 0.000007): 0.013308): 0.005666): 0.000005): 0.006365): 0.014844): 0.017213): 0.000012, (Dermochelys_coriacea: 0.025575, ((Eretmochelys_imbricata: 0.009355, (Caretta_caretta: 0.013437, (Lepidochelys_olivacea: 0.000002, Lepidochelys_kempii: 0.004538): 0.004613): 0.011077): 0.012855, (Chelonia_mydas: 0.011220, Natator_depressa: 0.020212): 0.004188): 0.005979): 0.004801): 0.000000): 0.023246): 0.001063): 0.050387);

**w ratios as labels for TreeView:**

((((((Anolis_carolinensis #0.00227106 , Iguana_iguana #0.00918017 ) #544.829 , Anguis_fragilis #0.0197009 ) #743.084 , Lacerta_viridis #0.0348695 ) #999 , Smaug_warreni #0.0171275 ) #999 , (Rena_humilis #0.0119731 , ((Python_regius #0.0212979 , Boa_constrictor #0.00181513 ) #661.905 , (Protobothrops_flavoviridis #0.0271007 , Dinodon_semicarinatus #0.0105857 ) #640.069 ) #999 ) #999 ) #985.455 , ((Rhea_americana #0.0295184 , (Gallus_gallus #0.0356784 , (Columba_livia #0.0260546 , (Grus_leucogeranus #0.0289142 , (Falco_peregrinus #0.0829885 , Pelecanus_occidentalis #0.0151122 ) #667.711 ) #0.0311298 ) #533.035 ) #0.0207866 ) #0.155006 , ((Alligator_mississippiensis #0.0893181 , Caiman_crocodylus #0.0641785 ) #58.2181 , (Crocodylus_porosus #0.00928632 , Gavialis_gangeticus #0.0134152 ) #681.139 ) #0.0592125 ) #0.0001 , (((Podocnemis_unifilis #0.0144966 , (Pelusios_castaneus #683.591 , Pelomedusa_subrufa #0.0328536 ) #0.00744596 ) #782.606 , ((Chelus_fimbriata #0.0416329 , (Platemys_platycephala #0.0518635 , Mesoclemmys_hogei #0.0231285 ) #0.0285053 ) #0.0225766 , (((Elusor_macrurus #0.0182745 , (Myuchelys_bellii #0.0198309 , Emydura_subglobosa #0.0638347 ) #0.12131 ) #0.078075 , (Chelodina_parkeri #0.0418036 , (Chelodina_rugosa #0.0398514 , (Chelodina_pritchardi #0.0168671 , (Chelodina_mccordi #0.0830678 , (Chelodina_longicollis #999 , Chelodina_expansa #999 ) #0.0924432 ) #0.0945303 ) #0.0001 ) #0.156013 ) #0.0655395 ) #0.0234329 , Pseudemydura_umbrina #0.23864 ) #58.7498 ) #0.00599512 ) #999 , ((Carettochelys_insculpta #0.0113265 , ((Lissemys_scutata #0.0240521 , Lissemys_punctata #0.997079 ) #0.090167 , ((Trionyx_triunguis #0.0455346 , (Pelochelys_cantorii #0.0274003 , Chitra_indica #0.0275653 ) #0.0670496 ) #0.0080148 , ((Rafetus_swinhoei #0.0783726 , (Apalone_spinifera #0.0345111 , Apalone_ferox #0.0506011 ) #0.101675 ) #0.0126059 , ((Amyda_cartilaginea #0.0652217 , Dogania_subplana #0.796545 ) #0.300897 , (Palea_steindachneri #0.0348761 , Pelodiscus_sinensis #0.0567826 ) #0.0001 ) #631.499 ) #437.776 ) #754.9 ) #0.0126617 ) #287.136 , (((Sternotherus_carinatus #0.284584 , Kinosternon_leucostomum #0.131287 ) #0.0945067 , (Macrochelys_temminckii #0.0506533 , Chelydra_serpentina #0.236193 ) #0.0661402 ) #803.7 , (((Platysternon_megacephalum #0.0715365 , (Chrysemys_picta #0.0861667 , (Trachemys_scripta #0.325644 , Malaclemys_terrapin #0.102723 ) #0.0711518 ) #0.0508128 ) #0.197821 , (((((Astrochelys_yniphora #0.103507 , Aldabrachelys_gigantea #0.116851 ) #0.0001 , Psammobates_pardalis #0.0699457 ) #0.0628094 , ((((Indotestudo_forstenii #0.474793 , Indotestudo_elongata #0.149828 ) #0.177729 , Testudo_horsfieldii #0.0979472 ) #0.0001 , (Testudo_graeca #0.0703345 , (Testudo_marginata #0.078128 , Testudo_kleinmanni #0.0819193 ) #974.949 ) #0.148925 ) #0.132562 , Malacochersus_tornieri #0.170659 ) #0.445945 ) #639.248 , (Manouria_impressa #0.114911 , Manouria_emys #0.189342 ) #0.0450267 ) #0.0163934 , ((Batagur_trivittata #0.105372 , Geoemyda_spengleri #0.0372486 ) #0.17278 , ((((Notochelys_platynota #0.189861 , (Cyclemys_fusca #0.101028 , ((Cyclemys_tcheponensis #0.0001 , Cyclemys_oldhamii #0.0001 ) #0.141451 , (Cyclemys_dentata #0.429871 , (Cyclemys_pulchristriata #0.0001 , Cyclemys_atripons #0.0001 ) #0.0857082 ) #0.143141 ) #0.0748399 ) #0.276033 ) #0.0001 , (Heosemys_annandalii #0.104917 , (Heosemys_grandis #0.11997 , Heosemys_depressa #0.190824 ) #129.97 ) #0.213433 ) #764.525 , (Sacalia_quadriocellata #0.0425707 , Sacalia_bealei #0.117944 ) #0.0354711 ) #0.0416083 , (((((Cuora_flavomarginata #0.113137 , (Cuora_trifasciata #0.412928 , (Cuora_pani #154.189 , Cuora_aurocapitata #157.158 ) #0.0644856 ) #0.246712 ) #0.0001 , (Cuora_mccordi #0.0001 , (Cuora_galbinifrons #0.153621 , (Cuora_picturata #0.127704 , Cuora_bourreti #0.230458 ) #0.164284 ) #0.0001 ) #0.0001 ) #0.0478127 , Cuora_mouhotii #0.371369 ) #0.0852302 , Cuora_amboinensis #0.0352214 ) #0.0001 , ((Mauremys_leprosa #0.0916868 , (((Mauremys_japonica #0.16612 , Mauremys_sinensis #0.15241 ) #0.0001 , ((Mauremys_reevesii #999 , NC_015101.1 #0.0001 ) #0.153557 , Mauremys_nigricans #0.180252 ) #0.344027 ) #0.0581821 , (Mauremys_rivulata #647.795 , Mauremys_caspica #730.292 ) #0.122994 ) #0.151246 ) #0.591674 , (Mauremys_mutica #0.215722 , Mauremys_annamensis #0.0001 ) #0.151535 ) #0.0941202 ) #0.0001 ) #0.026434 ) #0.111174 ) #0.056924 ) #0.0001 , (Dermochelys_coriacea #0.082189 , ((Eretmochelys_imbricata #0.0490182 , (Caretta_caretta #0.236025 , (Lepidochelys_olivacea #0.0001 , Lepidochelys_kempii #0.168871 ) #0.0366538 ) #0.190383 ) #0.196938 , (Chelonia_mydas #0.0424042 , Natator_depressa #0.0908399 ) #0.0350394 ) #0.0388474 ) #0.0106198 ) #0.0001 ) #421.918 ) #0.0001 ) #809.252 );

**ATP8**

**dS tree:**

((((((Anolis_carolinensis: 0.773819, Iguana_iguana: 4.423824): 0.000180, Anguis_fragilis: 0.000506): 13.516779, Lacerta_viridis: 0.846212): 0.000259, Smaug_warreni: 0.564236): 0.000000, (Rena_humilis: 8.008288, ((Python_regius: 3.233728, Boa_constrictor: 0.784777): 0.000202, (Protobothrops_flavoviridis: 9.455745, Dinodon_semicarinatus: 51.775411): 0.000004): 0.000375): 0.000489): 0.000265, ((Rhea_americana: 0.000165, (Gallus_gallus: 1.003812, (Columba_livia: 0.151547, (Grus_leucogeranus: 0.585561, (Falco_peregrinus: 0.662853, Pelecanus_occidentalis: 0.515343): 0.000093): 0.351009): 0.266395): 0.000004): 14.451878, ((Alligator_mississippiensis: 1.758602, Caiman_crocodylus: 52.147012): 0.000113, (Crocodylus_porosus: 3.395540, Gavialis_gangeticus: 0.000298): 0.000143): 52.193770): 0.000004, (((Podocnemis_unifilis: 0.613953, (Pelusios_castaneus: 1.287069, Pelomedusa_subrufa: 0.000207): 1.805140): 0.000093, ((Chelus_fimbriata: 0.656018, (Platemys_platycephala: 0.755520, Mesoclemmys_hogei: 0.000123): 52.434387): 17.665930, (((Elusor_macrurus: 0.293411, (Myuchelys_bellii: 0.077163, Emydura_subglobosa: 0.172368): 0.000024): 0.209749, (Chelodina_parkeri: 0.191153, (Chelodina_rugosa: 0.137284, (Chelodina_pritchardi: 0.000059, (Chelodina_mccordi: 0.034171, (Chelodina_longicollis: 0.000004, Chelodina_expansa: 0.000004): 0.000004): 0.080771): 0.061492): 0.253862): 0.410531): 0.000045, Pseudemydura_umbrina: 0.103896): 1.264702): 0.000137): 0.000054, ((Carettochelys_insculpta: 52.091853, ((Lissemys_scutata: 0.766273, Lissemys_punctata: 0.000046): 2.510630, ((Trionyx_triunguis: 0.225136, (Pelochelys_cantorii: 0.329775, Chitra_indica: 0.377843): 0.000004): 0.000004, ((Rafetus_swinhoei: 0.437533, (Apalone_spinifera: 0.487694, Apalone_ferox: 0.017139): 0.165375): 0.075380, ((Amyda_cartilaginea: 0.747389, Dogania_subplana: 0.052691): 0.000024, (Palea_steindachneri: 0.567147, Pelodiscus_sinensis: 0.000082): 0.584396): 0.000021): 0.000004): 1.635221): 0.000243): 0.000044, (((Sternotherus_carinatus: 0.078772, Kinosternon_leucostomum: 0.107209): 0.765596, (Macrochelys_temminckii: 0.144342, Chelydra_serpentina: 0.186374): 0.637580): 0.000073, (((Platysternon_megacephalum: 2.094060, (Chrysemys_picta: 0.131849, (Trachemys_scripta: 0.139613, Malaclemys_terrapin: 0.046194): 0.011163): 0.000085): 0.000004, (((((Astrochelys_yniphora: 0.000081, Aldabrachelys_gigantea: 0.243937): 0.125084, Psammobates_pardalis: 0.108779): 0.000022, ((((Indotestudo_forstenii: 0.000004, Indotestudo_elongata: 0.094838): 0.356982, Testudo_horsfieldii: 0.070641): 0.191383, (Testudo_graeca: 0.060282, (Testudo_marginata: 0.097634, Testudo_kleinmanni: 0.129191): 0.078197): 0.000048): 0.358767, Malacochersus_tornieri: 0.179697): 0.187028): 0.000096, (Manouria_impressa: 0.150149, Manouria_emys: 0.120399): 0.355782): 0.000004, ((Batagur_trivittata: 0.589764, Geoemyda_spengleri: 0.512740): 0.084960, ((((Notochelys_platynota: 0.324098, (Cyclemys_fusca: 0.000004, ((Cyclemys_tcheponensis: 0.000004, Cyclemys_oldhamii: 0.000004): 0.139008, (Cyclemys_dentata: 0.000012, (Cyclemys_pulchristriata: 0.000004, Cyclemys_atripons: 0.000004): 0.085240): 0.157676): 0.305873): 0.234664): 0.000033, (Heosemys_annandalii: 0.171309, (Heosemys_grandis: 0.146320, Heosemys_depressa: 0.319595): 0.000004): 0.000004): 0.000027, (Sacalia_quadriocellata: 0.085850, Sacalia_bealei: 0.022459): 0.046556): 0.065894, (((((Cuora_flavomarginata: 0.083520, (Cuora_trifasciata: 0.000000, (Cuora_pani: 0.000000, Cuora_aurocapitata: 0.000000): 0.000015): 0.053528): 0.000004, (Cuora_mccordi: 0.000000, (Cuora_galbinifrons: 0.063635, (Cuora_picturata: 0.058555, Cuora_bourreti: 0.000004): 0.000004): 0.027160): 0.000000): 0.096302, Cuora_mouhotii: 0.115694): 0.000004, Cuora_amboinensis: 0.102706): 0.035433, ((Mauremys_leprosa: 0.093293, (((Mauremys_japonica: 0.000026, Mauremys_sinensis: 0.182242): 0.027848, ((Mauremys_reevesii: 0.029727, NC_015101.1: 0.028976): 0.028811, Mauremys_nigricans: 0.000004): 0.000026): 0.026421, (Mauremys_rivulata: 0.000004, Mauremys_caspica: 0.000004): 0.031628): 0.033090): 0.000004, (Mauremys_mutica: 0.000057, Mauremys_annamensis: 0.233030): 0.030040): 0.143132): 0.056633): 0.000042): 0.333830): 0.472104): 0.188745, (Dermochelys_coriacea: 0.598943, ((Eretmochelys_imbricata: 0.331465, (Caretta_caretta: 0.411489, Lepidochelys_olivacea: 0.171966): 0.000004): 0.000112): 0.000000, (Chelonia_mydas: 0.000028, Natator_depressa: 0.410673): 0.150062): 1.441641): 0.086311): 0.000004): 0.000145): 4.232155);

**dN tree:**

((((((Anolis_carolinensis: 0.470097, Iguana_iguana: 0.141992): 0.120820, Anguis_fragilis: 0.505071): 0.001352, Lacerta_viridis: 0.708648): 0.199410, Smaug_warreni: 0.703981): 0.000002, (Rena_humilis: 0.453651, ((Python_regius: 0.778402, Boa_constrictor: 0.553200): 0.140858, (Protobothrops_flavoviridis: 0.245973, Dinodon_semicarinatus: 0.336108): 0.000000): 0.375110): 0.488454): 0.249265, ((Rhea_americana: 0.165228, (Gallus_gallus: 0.148144, (Columba_livia: 0.054199, (Grus_leucogeranus: 0.039484, (Falco_peregrinus: 0.094292, Pelecanus_occidentalis: 0.080396): 0.022707): 0.000035): 0.014326): 0.000000): 0.165051, ((Alligator_mississippiensis: 0.206820, Caiman_crocodylus: 0.163261): 0.042161, (Crocodylus_porosus: 0.061621, Gavialis_gangeticus: 0.297399): 0.083906): 0.141512): 0.000000, (((Podocnemis_unifilis: 0.176301, (Pelusios_castaneus: 0.055342, Pelomedusa_subrufa: 0.078812): 0.194537): 0.066516, ((Chelus_fimbriata: 0.080620, (Platemys_platycephala: 0.000076, Mesoclemmys_hogei: 0.048489): 0.029590): 0.089768, (((Elusor_macrurus: 0.013447, (Myuchelys_bellii: 0.020077, Emydura_subglobosa: 0.021867): 0.010078): 0.025798, (Chelodina_parkeri: 0.037832, (Chelodina_rugosa: 0.031624, (Chelodina_pritchardi: 0.059117, (Chelodina_mccordi: 0.011953, (Chelodina_longicollis: 0.000000, Chelodina_expansa: 0.000000): 0.000000): 0.045257): 0.000006): 0.006725): 0.055387): 0.014928, Pseudemydura_umbrina: 0.053510): 0.046050): 0.090298): 0.039320, ((Carettochelys_insculpta: 0.188918, ((Lissemys_scutata: 0.089666, Lissemys_punctata: 0.021458): 0.174754, ((Trionyx_triunguis: 0.079218, (Pelochelys_cantorii: 0.091656, Chitra_indica: 0.065225): 0.000000): 0.000000, ((Rafetus_swinhoei: 0.104744, (Apalone_spinifera: 0.069033, Apalone_ferox: 0.000002): 0.024501): 0.000008, ((Amyda_cartilaginea: 0.026466, Dogania_subplana: 0.037747): 0.011261, (Palea_steindachneri: 0.041178, Pelodiscus_sinensis: 0.042566): 0.025665): 0.012256): 0.000000): 0.000164): 0.242277): 0.027544, (((Sternotherus_carinatus: 0.021778, Kinosternon_leucostomum: 0.096787): 0.375269, (Macrochelys_temminckii: 0.036700, Chelydra_serpentina: 0.093229): 0.040918): 0.055819, (((Platysternon_megacephalum: 0.189306, (Chrysemys_picta: 0.090802, (Trachemys_scripta: 0.000014, Malaclemys_terrapin: 0.068982): 0.004212): 0.041776): 0.000000, (((((Astrochelys_yniphora: 0.079509, Aldabrachelys_gigantea: 0.041145): 0.000013, Psammobates_pardalis: 0.076247): 0.013115, ((((Indotestudo_forstenii: 0.000000, Indotestudo_elongata: 0.011726): 0.000036, Testudo_horsfieldii: 0.000007): 0.000019, (Testudo_graeca: 0.036007, (Testudo_marginata: 0.022938, Testudo_kleinmanni: 0.028431): 0.000008): 0.014659): 0.000036, Malacochersus_tornieri: 0.078597): 0.000019): 0.046048, (Manouria_impressa: 0.026319, Manouria_emys: 0.012433): 0.018559): 0.000000, ((Batagur_trivittata: 0.108317, Geoemyda_spengleri: 0.104779): 0.000008, ((((Notochelys_platynota: 0.107270, (Cyclemys_fusca: 0.000000, ((Cyclemys_tcheponensis: 0.000000, Cyclemys_oldhamii: 0.000000): 0.078455, (Cyclemys_dentata: 0.011757, (Cyclemys_pulchristriata: 0.000000, Cyclemys_atripons: 0.000000): 0.037262): 0.025660): 0.000031): 0.020036): 0.026699, (Heosemys_annandalii: 0.088708, (Heosemys_grandis: 0.055619, Heosemys_depressa: 0.056115): 0.000000): 0.000000): 0.020165, (Sacalia_quadriocellata: 0.050044, Sacalia_bealei: 0.024993): 0.024489): 0.000007, (((((Cuora_flavomarginata: 0.013544, (Cuora_trifasciata: 0.000002, (Cuora_pani: 0.000002, Cuora_aurocapitata: 0.000002): 0.013074): 0.013084): 0.000000, (Cuora_mccordi: 0.000002, (Cuora_galbinifrons: 0.012418, (Cuora_picturata: 0.000006, Cuora_bourreti: 0.000000): 0.000000): 0.039845): 0.000002): 0.000010, Cuora_mouhotii: 0.066015): 0.000000, Cuora_amboinensis: 0.039873): 0.000004, ((Mauremys_leprosa: 0.000009, (((Mauremys_japonica: 0.026226, Mauremys_sinensis: 0.026077): 0.000003, ((Mauremys_reevesii: 0.000003, NC_015101.1: 0.000003): 0.025813, Mauremys_nigricans: 0.000000): 0.025808): 0.000003, (Mauremys_rivulata: 0.000000, Mauremys_caspica: 0.000000): 0.066972): 0.000003): 0.000000, (Mauremys_mutica: 0.056654, Mauremys_annamensis: 0.026326): 0.000003): 0.038525): 0.000006): 0.010572): 0.070982): 0.000047): 0.000019, (Dermochelys_coriacea: 0.017506, ((Eretmochelys_imbricata: 0.055513, (Caretta_caretta: 0.036875, Lepidochelys_olivacea: 0.026332): 0.000000): 0.068837): 0.000002, (Chelonia_mydas: 0.013158, Natator_depressa: 0.025225): 0.012592): 0.047488): 0.013168): 0.000000): 0.145167): 0.000423);

**w ratios as labels for TreeView:**

((((((Anolis_carolinensis #0.607503 , Iguana_iguana #0.0320972 ) #670.754 , Anguis_fragilis #999 ) #0.0001 , Lacerta_viridis #0.837435 ) #770.931 , Smaug_warreni #1.24767 ) #20.534 , (Rena_humilis #0.0566477 , ((Python_regius #0.240714 , Boa_constrictor #0.704914 ) #696.185 , (Protobothrops_flavoviridis #0.0260131 , Dinodon_semicarinatus #0.00649165 ) #0.0001 ) #999 ) #999 ) #941.675 , ((Rhea_americana #999 , (Gallus_gallus #0.147581 , (Columba_livia #0.357639 , (Grus_leucogeranus #0.067429 , (Falco_peregrinus #0.142251 , Pelecanus_occidentalis #0.156005 ) #245.324 ) #0.0001 ) #0.0537775 ) #0.0001 ) #0.0114207 , ((Alligator_mississippiensis #0.117605 , Caiman_crocodylus #0.00313077 ) #372.662 , (Crocodylus_porosus #0.0181476 , Gavialis_gangeticus #999 ) #585.17 ) #0.00271127 ) #0.0001 , (((Podocnemis_unifilis #0.287157 , (Pelusios_castaneus #0.0429984 , Pelomedusa_subrufa #380.927 ) #0.107768 ) #715.611 , ((Chelus_fimbriata #0.122893 , (Platemys_platycephala #0.0001 , Mesoclemmys_hogei #394.182 ) #0.000564329 ) #0.00508142 , (((Elusor_macrurus #0.0458314 , (Myuchelys_bellii #0.260182 , Emydura_subglobosa #0.126865 ) #426.852 ) #0.122996 , (Chelodina_parkeri #0.197914 , (Chelodina_rugosa #0.230355 , (Chelodina_pritchardi #999 , (Chelodina_mccordi #0.349808 , (Chelodina_longicollis #0.0001 , Chelodina_expansa #0.0001 ) #0.0001 ) #0.560312 ) #0.0001 ) #0.0264902 ) #0.134915 ) #331.649 , Pseudemydura_umbrina #0.515031 ) #0.0364116 ) #657.508 ) #733.016 , ((Carettochelys_insculpta #0.00362662 , ((Lissemys_scutata #0.117015 , Lissemys_punctata #463.726 ) #0.0696055 , ((Trionyx_triunguis #0.351868 , (Pelochelys_cantorii #0.277935 , Chitra_indica #0.172626 ) #0.0001 ) #0.0001 , ((Rafetus_swinhoei #0.239397 , (Apalone_spinifera #0.14155 , Apalone_ferox #0.0001 ) #0.148152 ) #0.0001 , ((Amyda_cartilaginea #0.0354118 , Dogania_subplana #0.716384 ) #462.811 , (Palea_steindachneri #0.072605 , Pelodiscus_sinensis #517.96 ) #0.0439164 ) #590.984 ) #0.0001 ) #0.0001 ) #999 ) #621.926 , (((Sternotherus_carinatus #0.276473 , Kinosternon_leucostomum #0.902786 ) #0.490166 , (Macrochelys_temminckii #0.254257 , Chelydra_serpentina #0.500227 ) #0.0641769 ) #759.62 , (((Platysternon_megacephalum #0.0904013 , (Chrysemys_picta #0.688683 , (Trachemys_scripta #0.0001 , Malaclemys_terrapin #1.4933 ) #0.377361 ) #493.223 ) #0.0001 , (((((Astrochelys_yniphora #981.262 , Aldabrachelys_gigantea #0.168671 ) #0.0001 , Psammobates_pardalis #0.70094 ) #604.236 , ((((Indotestudo_forstenii #0.0001 , Indotestudo_elongata #0.12364 ) #0.0001 , Testudo_horsfieldii #0.0001 ) #0.0001 , (Testudo_graeca #0.59731 , (Testudo_marginata #0.234935 , Testudo_kleinmanni #0.220073 ) #0.0001 ) #305.573 ) #0.0001 , Malacochersus_tornieri #0.437389 ) #0.0001 ) #478.818 , (Manouria_impressa #0.175287 , Manouria_emys #0.103268 ) #0.0521627 ) #0.0001 , ((Batagur_trivittata #0.183661 , Geoemyda_spengleri #0.204352 ) #0.0001 , ((((Notochelys_platynota #0.330979 , (Cyclemys_fusca #0.0001 , ((Cyclemys_tcheponensis #0.0001 , Cyclemys_oldhamii #0.0001 ) #0.564391 , (Cyclemys_dentata #991.466 , (Cyclemys_pulchristriata #0.0001 , Cyclemys_atripons #0.0001 ) #0.437148 ) #0.162739 ) #0.0001 ) #0.085381 ) #804.668 , (Heosemys_annandalii #0.517828 , (Heosemys_grandis #0.380122 , Heosemys_depressa #0.17558 ) #0.0001 ) #0.0001 ) #737.526 , (Sacalia_quadriocellata #0.582923 , Sacalia_bealei #1.11281 ) #0.526008 ) #0.0001 , (((((Cuora_flavomarginata #0.162164 , (Cuora_trifasciata #18.7394 , (Cuora_pani #18.7122 , Cuora_aurocapitata #20.6029 ) #888.549 ) #0.244429 ) #0.0001 , (Cuora_mccordi #18.9051 , (Cuora_galbinifrons #0.195144 , (Cuora_picturata #0.0001 , Cuora_bourreti #0.0001 ) #0.000100033 ) #1.46704 ) #7.69856 ) #0.0001 , Cuora_mouhotii #0.570596 ) #0.0001 , Cuora_amboinensis #0.388219 ) #0.0001 , ((Mauremys_leprosa #0.0001 , (((Mauremys_japonica #999 , Mauremys_sinensis #0.143091 ) #0.0001 , ((Mauremys_reevesii #0.0001 , NC_015101.1 #0.0001 ) #0.895931 , Mauremys_nigricans #0.0001 ) #999 ) #0.0001 , (Mauremys_rivulata #0.0001 , Mauremys_caspica #0.0001 ) #2.11746 ) #0.0001 ) #0.0001 , (Mauremys_mutica #999 , Mauremys_annamensis #0.112973 ) #0.0001 ) #0.269158 ) #0.0001 ) #250.019 ) #0.21263 ) #0.0001 ) #0.0001 , (Dermochelys_coriacea #0.0292282 , ((Eretmochelys_imbricata #0.167479 , (Caretta_caretta #0.089614 , Lepidochelys_olivacea #0.153122 ) #0.0001 ) #612.51 ) #14.1337 , (Chelonia_mydas #465.09 , Natator_depressa #0.0614225 ) #0.0839114 ) #0.0329401 ) #0.152567 ) #0.0001 ) #999 ) #0.0001 );

**COX1**

**dS tree:**

((((((Anolis_carolinensis: 2.743888, Iguana_iguana: 1.781187): 0.005158, Anguis_fragilis: 2.225108): 0.000389, Lacerta_viridis: 1.812195): 0.270959, Smaug_warreni: 2.679756): 0.042684, (Rena_humilis: 5.824910, ((Python_regius: 1.754681, Boa_constrictor: 2.975597): 1.658134, (Protobothrops_flavoviridis: 2.454055, Dinodon_semicarinatus: 3.628490): 0.000005): 1.120529): 0.001212): 0.170119, ((Rhea_americana: 0.925973, (Gallus_gallus: 0.986392, (Columba_livia: 1.000305, (Grus_leucogeranus: 0.697062, (Falco_peregrinus: 0.932388, Pelecanus_occidentalis: 0.819414): 0.000005): 0.152136): 0.508591): 0.234607): 0.944844, ((Alligator_mississippiensis: 0.475553, Caiman_crocodylus: 0.783817): 0.615750, (Crocodylus_porosus: 1.119617, Gavialis_gangeticus: 1.065336): 0.678218): 1.276082): 0.000507, (((Podocnemis_unifilis: 2.602424, (Pelusios_castaneus: 0.595671, Pelomedusa_subrufa: 0.599188): 1.170619): 0.000759, ((Chelus_fimbriata: 0.695388, (Platemys_platycephala: 0.896513, Mesoclemmys_hogei: 0.152268): 0.514631): 0.626387, (((Elusor_macrurus: 0.307010, (Myuchelys_bellii: 0.046193, Emydura_subglobosa: 0.226213): 0.231206): 0.305870, (Chelodina_parkeri: 0.275601, (Chelodina_rugosa: 0.215144, (Chelodina_pritchardi: 0.088829, (Chelodina_mccordi: 0.060044, (Chelodina_longicollis: 0.000005, Chelodina_expansa: 0.004938): 0.034448): 0.008420): 0.175266): 0.197595): 0.940988): 0.371166, Pseudemydura_umbrina: 0.825658): 0.000065): 0.654233): 0.000392, ((Carettochelys_insculpta: 3.532506, ((Lissemys_scutata: 0.553315, Lissemys_punctata: 0.038319): 2.439882, ((Trionyx_triunguis: 0.333996, (Pelochelys_cantorii: 0.290692, Chitra_indica: 0.446149): 0.160886): 0.103070, ((Rafetus_swinhoei: 0.473558, (Apalone_spinifera: 0.218569, Apalone_ferox: 0.153436): 0.300702): 0.095811, ((Amyda_cartilaginea: 0.584717, Dogania_subplana: 0.537289): 0.164525, (Palea_steindachneri: 0.399615, Pelodiscus_sinensis: 0.729901): 0.000034): 0.177718): 0.195207): 0.366942): 0.773989): 0.000257, (((Sternotherus_carinatus: 0.210602, Kinosternon_leucostomum: 0.178803): 1.016078, (Macrochelys_temminckii: 0.318262, Chelydra_serpentina: 0.272313): 0.277757): 0.047985, (((Platysternon_megacephalum: 1.004940, (Chrysemys_picta: 0.144833, (Trachemys_scripta: 0.091169, Malaclemys_terrapin: 0.083414): 0.102213): 0.301457): 0.000388, (((((Astrochelys_yniphora: 0.290328, Aldabrachelys_gigantea: 0.187514): 0.046206, Psammobates_pardalis: 0.357944): 0.029969, ((((Indotestudo_forstenii: 0.073279, Indotestudo_elongata: 0.067693): 0.145447, Testudo_horsfieldii: 0.194378): 0.000005, (Testudo_graeca: 0.126120, (Testudo_marginata: 0.128076, Testudo_kleinmanni: 0.120982): 0.047456): 0.114622): 0.030879, Malacochersus_tornieri: 0.389472): 0.091503): 0.113113, (Manouria_impressa: 0.177019, Manouria_emys: 0.178427): 0.132962): 0.094291, ((Batagur_trivittata: 0.685669, Geoemyda_spengleri: 0.482707): 0.086959, ((((Notochelys_platynota: 0.314434, (Cyclemys_fusca: 0.205153, ((Cyclemys_tcheponensis: 0.021523, Cyclemys_oldhamii: 0.005178): 0.182151, (Cyclemys_dentata: 0.052953, (Cyclemys_pulchristriata: 0.007412, Cyclemys_atripons: 0.013716): 0.053325): 0.077856): 0.049449): 0.061050): 0.000005, (Heosemys_annandalii: 0.202229, (Heosemys_grandis: 0.156497, Heosemys_depressa: 0.105289): 0.056052): 0.237630): 0.020375, (Sacalia_quadriocellata: 0.173927, Sacalia_bealei: 0.240186): 0.064772): 0.073631, (((((Cuora_flavomarginata: 0.116389, (Cuora_trifasciata: 0.031151, (Cuora_pani: 0.000065, Cuora_aurocapitata: 0.023575): 0.053177): 0.041695): 0.027830, (Cuora_mccordi: 0.090421, (Cuora_galbinifrons: 0.050680, (Cuora_picturata: 0.000014, Cuora_bourreti: 0.000015): 0.081478): 0.071629): 0.012529): 0.000005, Cuora_mouhotii: 0.144326): 0.024212, Cuora_amboinensis: 0.259821): 0.121102, ((Mauremys_leprosa: 0.112306, (((Mauremys_japonica: 0.074933, Mauremys_sinensis: 0.074363): 0.006181, ((Mauremys_reevesii: 0.004595, NC_015101.1: 0.000001): 0.057811, Mauremys_nigricans: 0.046199): 0.011203): 0.063326, (Mauremys_rivulata: 0.000001, Mauremys_caspica: 0.000001): 0.102803): 0.024674): 0.002577, (Mauremys_mutica: 0.085481, Mauremys_annamensis: 0.100387): 0.073774): 0.024109): 0.122642): 0.077359): 0.045254): 0.177545): 0.035190, (Dermochelys_coriacea: 0.448553, ((Eretmochelys_imbricata: 0.164662, (Caretta_caretta: 0.139131, (Lepidochelys_olivacea: 0.028997, Lepidochelys_kempii: 0.022849): 0.095658): 0.068058): 0.116009, (Chelonia_mydas: 0.179957, Natator_depressa: 0.159015): 0.148949): 0.072181): 0.313739): 0.092237): 0.292934): 1.099575): 1.601630);

**dN tree:**

((((((Anolis_carolinensis: 0.014016, Iguana_iguana: 0.010178): 0.003783, Anguis_fragilis: 0.032341): 0.004010, Lacerta_viridis: 0.024267): 0.004312, Smaug_warreni: 0.030133): 0.007025, (Rena_humilis: 0.077300, ((Python_regius: 0.010711, Boa_constrictor: 0.011735): 0.009937, (Protobothrops_flavoviridis: 0.016668, Dinodon_semicarinatus: 0.012417): 0.000000): 0.073420): 0.063539): 0.007615, ((Rhea_americana: 0.007438, (Gallus_gallus: 0.012829, (Columba_livia: 0.003689, (Grus_leucogeranus: 0.003731, (Falco_peregrinus: 0.013199, Pelecanus_occidentalis: 0.002749): 0.000000): 0.000015): 0.002193): 0.000276): 0.026143, ((Alligator_mississippiensis: 0.009520, Caiman_crocodylus: 0.019537): 0.009641, (Crocodylus_porosus: 0.016884, Gavialis_gangeticus: 0.009443): 0.009921): 0.036701): 0.008882, (((Podocnemis_unifilis: 0.032379, (Pelusios_castaneus: 0.029208, Pelomedusa_subrufa: 0.013911): 0.048004): 0.013175, ((Chelus_fimbriata: 0.009859, (Platemys_platycephala: 0.007826, Mesoclemmys_hogei: 0.010209): 0.004573): 0.000063, (((Elusor_macrurus: 0.002713, (Myuchelys_bellii: 0.000005, Emydura_subglobosa: 0.001828): 0.000023): 0.008038, (Chelodina_parkeri: 0.000880, (Chelodina_rugosa: 0.001792, (Chelodina_pritchardi: 0.000893, (Chelodina_mccordi: 0.000006, (Chelodina_longicollis: 0.000000, Chelodina_expansa: 0.000000): 0.000887): 0.000001): 0.000018): 0.001810): 0.003988): 0.004624, Pseudemydura_umbrina: 0.011679): 0.001528): 0.023174): 0.013299, ((Carettochelys_insculpta: 0.037630, ((Lissemys_scutata: 0.002721, Lissemys_punctata: 0.006423): 0.010283, ((Trionyx_triunguis: 0.002760, (Pelochelys_cantorii: 0.003692, Chitra_indica: 0.002682): 0.000016): 0.000920, ((Rafetus_swinhoei: 0.001847, (Apalone_spinifera: 0.016325, Apalone_ferox: 0.002754): 0.002732): 0.000910, ((Amyda_cartilaginea: 0.002847, Dogania_subplana: 0.008290): 0.000921, (Palea_steindachneri: 0.002800, Pelodiscus_sinensis: 0.033421): 0.000918): 0.001751): 0.000911): 0.000037): 0.006980): 0.004296, (((Sternotherus_carinatus: 0.002344, Kinosternon_leucostomum: 0.003186): 0.019141, (Macrochelys_temminckii: 0.003774, Chelydra_serpentina: 0.003617): 0.003988): 0.000005, (((Platysternon_megacephalum: 0.028106, (Chrysemys_picta: 0.006197, (Trachemys_scripta: 0.003984, Malaclemys_terrapin: 0.003428): 0.001843): 0.002100): 0.000000, (((((Astrochelys_yniphora: 0.002721, Aldabrachelys_gigantea: 0.002708): 0.000907, Psammobates_pardalis: 0.001845): 0.000003, ((((Indotestudo_forstenii: 0.002801, Indotestudo_elongata: 0.001776): 0.001824, Testudo_horsfieldii: 0.005519): 0.000000, (Testudo_graeca: 0.008230, (Testudo_marginata: 0.002797, Testudo_kleinmanni: 0.005454): 0.002719): 0.000918): 0.000853, Malacochersus_tornieri: 0.021232): 0.000009): 0.000926, (Manouria_impressa: 0.013208, Manouria_emys: 0.004729): 0.002767): 0.000009, ((Batagur_trivittata: 0.006963, Geoemyda_spengleri: 0.006210): 0.000009, ((((Notochelys_platynota: 0.005636, (Cyclemys_fusca: 0.012209, ((Cyclemys_tcheponensis: 0.008914, Cyclemys_oldhamii: 0.007252): 0.001799, (Cyclemys_dentata: 0.002841, (Cyclemys_pulchristriata: 0.002836, Cyclemys_atripons: 0.000945): 0.001896): 0.000937): 0.000005): 0.000006): 0.000000, (Heosemys_annandalii: 0.008319, (Heosemys_grandis: 0.001857, Heosemys_depressa: 0.000011): 0.001846): 0.000024): 0.002800, (Sacalia_quadriocellata: 0.003749, Sacalia_bealei: 0.010800): 0.000006): 0.000007, (((((Cuora_flavomarginata: 0.002820, (Cuora_trifasciata: 0.001899, (Cuora_pani: 0.000941, Cuora_aurocapitata: 0.000002): 0.000944): 0.000004): 0.000003, (Cuora_mccordi: 0.006856, (Cuora_galbinifrons: 0.000950, (Cuora_picturata: 0.000944, Cuora_bourreti: 0.000954): 0.001909): 0.000950): 0.000001): 0.000000, Cuora_mouhotii: 0.003753): 0.000942, Cuora_amboinensis: 0.001888): 0.000012, ((Mauremys_leprosa: 0.020203, (((Mauremys_japonica: 0.001871, Mauremys_sinensis: 0.000934): 0.000934, ((Mauremys_reevesii: 0.000000, NC_015101.1: 0.000001): 0.000006, Mauremys_nigricans: 0.002810): 0.000001): 0.000006, (Mauremys_rivulata: 0.000002, Mauremys_caspica: 0.000002): 0.002840): 0.000002): 0.000000, (Mauremys_mutica: 0.000009, Mauremys_annamensis: 0.001891): 0.000940): 0.000002): 0.000012): 0.002332): 0.003238): 0.000934): 0.000004, (Dermochelys_coriacea: 0.004519, ((Eretmochelys_imbricata: 0.007179, (Caretta_caretta: 0.000891, (Lepidochelys_olivacea: 0.000897, Lepidochelys_kempii: 0.000002): 0.001790): 0.000883): 0.000901, (Chelonia_mydas: 0.003548, Natator_depressa: 0.000016): 0.000888): 0.000007): 0.003628): 0.002685): 0.002661): 0.003639): 0.011110);

**w ratios as labels for TreeView:**

((((((Anolis_carolinensis #0.00510801 , Iguana_iguana #0.00571393 ) #0.733314 , Anguis_fragilis #0.0145345 ) #10.297 , Lacerta_viridis #0.0133912 ) #0.0159156 , Smaug_warreni #0.0112448 ) #0.164592 , (Rena_humilis #0.0132706 , ((Python_regius #0.00610411 , Boa_constrictor #0.00394383 ) #0.00599297 , (Protobothrops_flavoviridis #0.00679188 , Dinodon_semicarinatus #0.003422 ) #0.0001 ) #0.0655223 ) #52.4349 ) #0.0447643 , ((Rhea_americana #0.00803267 , (Gallus_gallus #0.0130055 , (Columba_livia #0.00368773 , (Grus_leucogeranus #0.00535197 , (Falco_peregrinus #0.0141556 , Pelecanus_occidentalis #0.00335446 ) #0.0001 ) #0.0001 ) #0.00431258 ) #0.0011748 ) #0.0276696 , ((Alligator_mississippiensis #0.0200178 , Caiman_crocodylus #0.0249251 ) #0.0156576 , (Crocodylus_porosus #0.01508 , Gavialis_gangeticus #0.00886341 ) #0.0146284 ) #0.0287604 ) #17.531 , (((Podocnemis_unifilis #0.012442 , (Pelusios_castaneus #0.0490339 , Pelomedusa_subrufa #0.023216 ) #0.0410077 ) #17.3554 , ((Chelus_fimbriata #0.014178 , (Platemys_platycephala #0.00872948 , Mesoclemmys_hogei #0.067049 ) #0.00888535 ) #0.0001 , (((Elusor_macrurus #0.00883674 , (Myuchelys_bellii #0.0001 , Emydura_subglobosa #0.00808298 ) #0.0001 ) #0.0262794 , (Chelodina_parkeri #0.0031914 , (Chelodina_rugosa #0.00832815 , (Chelodina_pritchardi #0.0100535 , (Chelodina_mccordi #0.0001 , (Chelodina_longicollis #0.0001 , Chelodina_expansa #0.0001 ) #0.0257413 ) #0.0001 ) #0.0001 ) #0.00915939 ) #0.00423836 ) #0.0124582 , Pseudemydura_umbrina #0.0141445 ) #23.6769 ) #0.035422 ) #33.8918 , ((Carettochelys_insculpta #0.0106525 , ((Lissemys_scutata #0.00491728 , Lissemys_punctata #0.167623 ) #0.00421461 , ((Trionyx_triunguis #0.00826364 , (Pelochelys_cantorii #0.0127007 , Chitra_indica #0.00601226 ) #0.0001 ) #0.00892657 , ((Rafetus_swinhoei #0.00390098 , (Apalone_spinifera #0.074688 , Apalone_ferox #0.0179498 ) #0.00908667 ) #0.00949435 , ((Amyda_cartilaginea #0.00486941 , Dogania_subplana #0.0154285 ) #0.00559552 , (Palea_steindachneri #0.00700732 , Pelodiscus_sinensis #0.0457881 ) #27.02 ) #0.00985221 ) #0.00466845 ) #0.0001 ) #0.00901792 ) #16.7123 , (((Sternotherus_carinatus #0.0111298 , Kinosternon_leucostomum #0.0178188 ) #0.0188384 , (Macrochelys_temminckii #0.0118586 , Chelydra_serpentina #0.013283 ) #0.0143582 ) #0.0001 , (((Platysternon_megacephalum #0.0279675 , (Chrysemys_picta #0.0427869 , (Trachemys_scripta #0.0436991 , Malaclemys_terrapin #0.041102 ) #0.0180278 ) #0.00696596 ) #0.0001 , (((((Astrochelys_yniphora #0.00937259 , Aldabrachelys_gigantea #0.0144439 ) #0.019631 , Psammobates_pardalis #0.00515515 ) #0.0001 , ((((Indotestudo_forstenii #0.0382196 , Indotestudo_elongata #0.0262295 ) #0.0125387 , Testudo_horsfieldii #0.0283924 ) #0.0001 , (Testudo_graeca #0.0652577 , (Testudo_marginata #0.0218358 , Testudo_kleinmanni #0.0450823 ) #0.0572934 ) #0.00801164 ) #0.0276125 , Malacochersus_tornieri #0.0545153 ) #0.0001 ) #0.00818409 , (Manouria_impressa #0.0746145 , Manouria_emys #0.0265051 ) #0.020811 ) #0.0001 , ((Batagur_trivittata #0.0101549 , Geoemyda_spengleri #0.0128656 ) #0.0001 , ((((Notochelys_platynota #0.0179236 , (Cyclemys_fusca #0.0595127 , ((Cyclemys_tcheponensis #0.414142 , Cyclemys_oldhamii #1.4005 ) #0.00987596 , (Cyclemys_dentata #0.0536507 , (Cyclemys_pulchristriata #0.382595 , Cyclemys_atripons #0.0688835 ) #0.0355615 ) #0.0120384 ) #0.0001 ) #0.0001 ) #0.0001 , (Heosemys_annandalii #0.0411374 , (Heosemys_grandis #0.0118688 , Heosemys_depressa #0.0001 ) #0.0329415 ) #0.0001 ) #0.137445 , (Sacalia_quadriocellata #0.0215567 , Sacalia_bealei #0.0449663 ) #0.0001 ) #0.0001 , (((((Cuora_flavomarginata #0.0242257 , (Cuora_trifasciata #0.0609707 , (Cuora_pani #14.388 , Cuora_aurocapitata #0.0001 ) #0.0177569 ) #0.0001 ) #0.0001 , (Cuora_mccordi #0.0758239 , (Cuora_galbinifrons #0.0187474 , (Cuora_picturata #65.2259 , Cuora_bourreti #65.0124 ) #0.0234342 ) #0.0132656 ) #0.0001 ) #0.0001 , Cuora_mouhotii #0.026006 ) #0.0389046 , Cuora_amboinensis #0.00726846 ) #0.0001 , ((Mauremys_leprosa #0.179896 , (((Mauremys_japonica #0.0249624 , Mauremys_sinensis #0.012557 ) #0.151138 , ((Mauremys_reevesii #0.0001 , NC_015101.1 #1.59232 ) #0.0001 , Mauremys_nigricans #0.0608272 ) #0.0001 ) #0.0001 , (Mauremys_rivulata #2.54736 , Mauremys_caspica #2.53669 ) #0.0276283 ) #0.0001 ) #0.0001 , (Mauremys_mutica #0.0001 , Mauremys_annamensis #0.018839 ) #0.012747 ) #0.0001 ) #0.0001 ) #0.0301389 ) #0.0715478 ) #0.00526258 ) #0.0001 , (Dermochelys_coriacea #0.0100737 , ((Eretmochelys_imbricata #0.0435984 , (Caretta_caretta #0.00640646 , (Lepidochelys_olivacea #0.0309272 , Lepidochelys_kempii #0.0001 ) #0.0187088 ) #0.0129686 ) #0.00776485 , (Chelonia_mydas #0.0197173 , Natator_depressa #0.0001 ) #0.00596066 ) #0.0001 ) #0.0115637 ) #0.0291102 ) #0.00908447 ) #0.00330936 ) #0.0069366 );

**COX2**

**dS tree:**

((((((Anolis_carolinensis: 4.755722, Iguana_iguana: 0.952705): 0.000019, Anguis_fragilis: 2.705787): 0.000039, Lacerta_viridis: 3.755242): 0.812047, Smaug_warreni: 2.769742): 0.000017, (Rena_humilis: 4.868489, ((Python_regius: 2.029944, Boa_constrictor: 74.299855): 0.000014, (Protobothrops_flavoviridis: 2.112411, Dinodon_semicarinatus: 2.047500): 0.000018): 2.924590): 0.461334): 0.000029, ((Rhea_americana: 1.276498, (Gallus_gallus: 1.169872, (Columba_livia: 0.705524, (Grus_leucogeranus: 0.583303, (Falco_peregrinus: 1.234151, Pelecanus_occidentalis: 0.748135): 0.399765): 0.000006): 0.599282): 0.000016): 1.130340, ((Alligator_mississippiensis: 1.158206, Caiman_crocodylus: 0.755609): 0.508057, (Crocodylus_porosus: 1.476499, Gavialis_gangeticus: 2.651143): 0.106296): 2.141041): 0.000023, (((Podocnemis_unifilis: 1.756276, (Pelusios_castaneus: 0.380702, Pelomedusa_subrufa: 0.740039): 1.785478): 0.963724, ((Chelus_fimbriata: 1.290451, (Platemys_platycephala: 0.359170, Mesoclemmys_hogei: 0.587092): 0.059298): 1.253937, (((Elusor_macrurus: 0.352956, (Myuchelys_bellii: 0.128301, Emydura_subglobosa: 0.241226): 0.069610): 0.182164, (Chelodina_parkeri: 0.371683, (Chelodina_rugosa: 0.207612, (Chelodina_pritchardi: 0.161078, (Chelodina_mccordi: 0.055915, (Chelodina_longicollis: 0.000000, Chelodina_expansa: 0.000000): 0.014898): 0.030262): 0.119553): 0.063857): 1.738308): 0.590635, Pseudemydura_umbrina: 0.631420): 0.555693): 0.621878): 0.000055, ((Carettochelys_insculpta: 74.203298, ((Lissemys_scutata: 0.598262, Lissemys_punctata: 0.000054): 3.808035, ((Trionyx_triunguis: 0.786349, (Pelochelys_cantorii: 0.444966, Chitra_indica: 0.311793): 0.000006): 0.269834, ((Rafetus_swinhoei: 0.177218, (Apalone_spinifera: 0.115522, Apalone_ferox: 0.107342): 0.355154): 0.082643, ((Amyda_cartilaginea: 0.388657, Dogania_subplana: 0.502084): 0.126129, (Palea_steindachneri: 0.394326, Pelodiscus_sinensis: 0.641055): 0.000005): 0.216418): 0.000006): 0.000007): 0.000006): 0.641789, (((Sternotherus_carinatus: 0.511825, Kinosternon_leucostomum: 0.000007): 0.972726, (Macrochelys_temminckii: 0.324578, Chelydra_serpentina: 0.356269): 0.120481): 0.156522, (((Platysternon_megacephalum: 0.476006, (Chrysemys_picta: 0.149418, (Trachemys_scripta: 0.211713, Malaclemys_terrapin: 0.011458): 0.194242): 0.217894): 0.019475, (((((Astrochelys_yniphora: 0.193755, Aldabrachelys_gigantea: 0.149725): 0.137593, Psammobates_pardalis: 0.319518): 0.130792, ((((Indotestudo_forstenii: 0.062368, Indotestudo_elongata: 0.104162): 0.062253, Testudo_horsfieldii: 0.215780): 0.072212, (Testudo_graeca: 0.189786, (Testudo_marginata: 0.116655, Testudo_kleinmanni: 0.204964): 0.001155): 0.180541): 0.057006, Malacochersus_tornieri: 0.188826): 0.145344): 0.000006, (Manouria_impressa: 0.260490, Manouria_emys: 0.135549): 0.159103): 0.183816, ((Batagur_trivittata: 0.498347, Geoemyda_spengleri: 0.632208): 0.000004, ((((Notochelys_platynota: 0.261684, (Cyclemys_fusca: 0.018827, ((Cyclemys_tcheponensis: 0.006981, Cyclemys_oldhamii: 0.000006): 0.007030, (Cyclemys_dentata: 0.059458, (Cyclemys_pulchristriata: 0.013271, Cyclemys_atripons: 0.014050): 0.005507): 0.294108): 0.002602): 0.382157): 0.041021, (Heosemys_annandalii: 0.169606, (Heosemys_grandis: 0.198403, Heosemys_depressa: 0.160502): 0.058380): 0.201212): 0.000011, (Sacalia_quadriocellata: 0.155753, Sacalia_bealei: 0.213590): 0.100439): 0.076913, (((((Cuora_flavomarginata: 0.129723, (Cuora_trifasciata: 0.089110, (Cuora_pani: 0.000000, Cuora_aurocapitata: 0.010878): 0.068060): 0.047462): 0.009956, (Cuora_mccordi: 0.034300, (Cuora_galbinifrons: 0.128504, (Cuora_picturata: 0.000002, Cuora_bourreti: 0.006844): 0.025727): 0.189773): 0.000006): 0.112603, Cuora_mouhotii: 0.134151): 0.065541, Cuora_amboinensis: 0.182780): 0.072934, ((Mauremys_leprosa: 0.122556, (((Mauremys_japonica: 0.112705, Mauremys_sinensis: 0.049813): 0.001986, ((Mauremys_reevesii: 0.000000): 0.033357, Mauremys_nigricans: 0.036366): 0.056723): 0.055412, (Mauremys_rivulata: 0.000006, Mauremys_caspica: 0.000006): 0.127027): 0.035174): 0.000002, (Mauremys_mutica: 0.076581, Mauremys_annamensis: 0.105787): 0.059643): 0.122187): 0.082071): 0.063520): 0.000009): 0.319832): 0.000006, (Dermochelys_coriacea: 0.419970, ((Eretmochelys_imbricata: 0.140659, (Caretta_caretta: 0.195935, (Lepidochelys_olivacea: 0.000006, Lepidochelys_kempii: 0.074899): 0.177292): 0.135543): 0.149409, (Chelonia_mydas: 0.184557, Natator_depressa: 0.304788): 0.087315): 0.075842): 0.237900): 0.071933): 0.403480): 1.747003): 2.155577);

**dN tree:**

((((((Anolis_carolinensis: 0.043062, Iguana_iguana: 0.019162): 0.012487, Anguis_fragilis: 0.117954): 0.012528, Lacerta_viridis: 0.070760): 0.003994, Smaug_warreni: 0.056809): 0.013437, (Rena_humilis: 0.171921, ((Python_regius: 0.039675, Boa_constrictor: 0.028876): 0.011641, (Protobothrops_flavoviridis: 0.035369, Dinodon_semicarinatus: 0.042947): 0.017530): 0.192061): 0.130555): 0.029085, ((Rhea_americana: 0.010651, (Gallus_gallus: 0.025354, (Columba_livia: 0.015885, (Grus_leucogeranus: 0.011561, (Falco_peregrinus: 0.026681, Pelecanus_occidentalis: 0.014138): 0.000040): 0.000000): 0.002884): 0.008926): 0.127795, ((Alligator_mississippiensis: 0.027882, Caiman_crocodylus: 0.038710): 0.052281, (Crocodylus_porosus: 0.033601, Gavialis_gangeticus: 0.022561): 0.031968): 0.107321): 0.023110, (((Podocnemis_unifilis: 0.023143, (Pelusios_castaneus: 0.022662, Pelomedusa_subrufa: 0.056296): 0.075594): 0.035270, ((Chelus_fimbriata: 0.024213, (Platemys_platycephala: 0.010460, Mesoclemmys_hogei: 0.005507): 0.003665): 0.010017, (((Elusor_macrurus: 0.001940, (Myuchelys_bellii: 0.003869, Emydura_subglobosa: 0.000024): 0.001942): 0.008600, (Chelodina_parkeri: 0.009940, (Chelodina_rugosa: 0.000021, (Chelodina_pritchardi: 0.002011, (Chelodina_mccordi: 0.000006, (Chelodina_longicollis: 0.000002, Chelodina_expansa: 0.000002): 0.000001): 0.000003): 0.000012): 0.000006): 0.013306): 0.002947, Pseudemydura_umbrina: 0.023757): 0.000056): 0.032922): 0.015132, ((Carettochelys_insculpta: 0.056751, ((Lissemys_scutata: 0.005467, Lissemys_punctata: 0.016243): 0.021243, ((Trionyx_triunguis: 0.010958, (Pelochelys_cantorii: 0.012894, Chitra_indica: 0.004678): 0.005613): 0.006313, ((Rafetus_swinhoei: 0.007652, (Apalone_spinifera: 0.001912, Apalone_ferox: 0.001909): 0.000036): 0.000008, ((Amyda_cartilaginea: 0.007634, Dogania_subplana: 0.019533): 0.001818, (Palea_steindachneri: 0.003811, Pelodiscus_sinensis: 0.001924): 0.001888): 0.003834): 0.000000): 0.007337): 0.005495): 0.004701, (((Sternotherus_carinatus: 0.010159, Kinosternon_leucostomum: 0.003715): 0.044539, (Macrochelys_temminckii: 0.001907, Chelydra_serpentina: 0.018320): 0.011909): 0.000016, (((Platysternon_megacephalum: 0.016783, (Chrysemys_picta: 0.014112, (Trachemys_scripta: 0.012221, Malaclemys_terrapin: 0.004089): 0.000019): 0.006250): 0.000002, (((((Astrochelys_yniphora: 0.003747, Aldabrachelys_gigantea: 0.005820): 0.000014, Psammobates_pardalis: 0.011040): 0.002663, ((((Indotestudo_forstenii: 0.007937, Indotestudo_elongata: 0.001850): 0.011896, Testudo_horsfieldii: 0.010010): 0.000007, (Testudo_graeca: 0.009921, (Testudo_marginata: 0.007853, Testudo_kleinmanni: 0.011863): 0.000000): 0.007787): 0.001898, Malacochersus_tornieri: 0.041815): 0.001996): 0.000000, (Manouria_impressa: 0.011932, Manouria_emys: 0.003886): 0.007932): 0.000018, ((Batagur_trivittata: 0.007913, Geoemyda_spengleri: 0.020157): 0.004277, ((((Notochelys_platynota: 0.047396, (Cyclemys_fusca: 0.000002, ((Cyclemys_tcheponensis: 0.000001, Cyclemys_oldhamii: 0.000000): 0.001954, (Cyclemys_dentata: 0.003951, (Cyclemys_pulchristriata: 0.000001, Cyclemys_atripons: 0.000001): 0.000001): 0.006085): 0.000000): 0.000038): 0.000004, (Heosemys_annandalii: 0.003959, (Heosemys_grandis: 0.005864, Heosemys_depressa: 0.005957): 0.001987): 0.000020): 0.001953, (Sacalia_quadriocellata: 0.005954, Sacalia_bealei: 0.001999): 0.001966): 0.003513, (((((Cuora_flavomarginata: 0.004016, (Cuora_trifasciata: 0.004122, (Cuora_pani: 0.000002, Cuora_aurocapitata: 0.028219): 0.002030): 0.004081): 0.000001, (Cuora_mccordi: 0.000003, (Cuora_galbinifrons: 0.000013, (Cuora_picturata: 0.001956, Cuora_bourreti: 0.000001): 0.003934): 0.003996): 0.000000): 0.000011, Cuora_mouhotii: 0.010136): 0.000007, Cuora_amboinensis: 0.000018): 0.000007, ((Mauremys_leprosa: 0.001540, (((Mauremys_japonica: 0.001918, Mauremys_sinensis: 0.001927): 0.000000, ((Mauremys_reevesii: 0.000002, NC_015101.1: 0.000002): 0.001979, Mauremys_nigricans: 0.001982): 0.001950): 0.002346, (Mauremys_rivulata: 0.000000, Mauremys_caspica: 0.000000): 0.007354): 0.000004): 0.002368, (Mauremys_mutica: 0.000008, Mauremys_annamensis: 0.001991): 0.000006): 0.000012): 0.004345): 0.001927): 0.009431): 0.007873): 0.000000, (Dermochelys_coriacea: 0.003778, ((Eretmochelys_imbricata: 0.007727, (Caretta_caretta: 0.003275, (Lepidochelys_olivacea: 0.000000, Lepidochelys_kempii: 0.001897): 0.002457): 0.003673): 0.000015, (Chelonia_mydas: 0.000018, Natator_depressa: 0.003751): 0.001911): 0.000008): 0.005848): 0.007737): 0.000040): 0.000175): 0.013973);

**w ratios as labels for TreeView:**

((((((Anolis_carolinensis #0.00905486 , Iguana_iguana #0.0201128 ) #663.891 , Anguis_fragilis #0.0435932 ) #324.997 , Lacerta_viridis #0.018843 ) #0.00491876 , Smaug_warreni #0.0205107 ) #778.528 , (Rena_humilis #0.035313 , ((Python_regius #0.0195451 , Boa_constrictor #0.000388642 ) #811.825 , (Protobothrops_flavoviridis #0.0167434 , Dinodon_semicarinatus #0.0209753 ) #999 ) #0.065671 ) #0.282995 ) #999 , ((Rhea_americana #0.00834409 , (Gallus_gallus #0.0216725 , (Columba_livia #0.0225148 , (Grus_leucogeranus #0.0198204 , (Falco_peregrinus #0.0216188 , Pelecanus_occidentalis #0.0188977 ) #0.0001 ) #0.0001 ) #0.00481178 ) #556.99 ) #0.113059 , ((Alligator_mississippiensis #0.0240738 , Caiman_crocodylus #0.0512301 ) #0.102905 , (Crocodylus_porosus #0.0227576 , Gavialis_gangeticus #0.00851009 ) #0.300748 ) #0.0501256 ) #999 , (((Podocnemis_unifilis #0.0131772 , (Pelusios_castaneus #0.059528 , Pelomedusa_subrufa #0.0760721 ) #0.0423383 ) #0.0365975 , ((Chelus_fimbriata #0.0187632 , (Platemys_platycephala #0.0291217 , Mesoclemmys_hogei #0.00937972 ) #0.0618037 ) #0.0079885 , (((Elusor_macrurus #0.0054964 , (Myuchelys_bellii #0.0301583 , Emydura_subglobosa #0.0001 ) #0.027896 ) #0.0472105 , (Chelodina_parkeri #0.0267433 , (Chelodina_rugosa #0.0001 , (Chelodina_pritchardi #0.0124831 , (Chelodina_mccordi #0.0001 , (Chelodina_longicollis #111.514 , Chelodina_expansa #419.574 ) #0.0001 ) #0.0001 ) #0.0001 ) #0.0001 ) #0.00765464 ) #0.00499023 , Pseudemydura_umbrina #0.0376252 ) #0.0001 ) #0.0529404 ) #277.409 , ((Carettochelys_insculpta #0.000764801 , ((Lissemys_scutata #0.00913758 , Lissemys_punctata #300.873 ) #0.00557849 , ((Trionyx_triunguis #0.0139349 , (Pelochelys_cantorii #0.028978 , Chitra_indica #0.0150023 ) #994.927 ) #0.0233946 , ((Rafetus_swinhoei #0.0431767 , (Apalone_spinifera #0.0165532 , Apalone_ferox #0.017781 ) #0.0001 ) #0.0001 , ((Amyda_cartilaginea #0.0196414 , Dogania_subplana #0.038903 ) #0.0144175 , (Palea_steindachneri #0.00966341 , Pelodiscus_sinensis #0.0030008 ) #385.172 ) #0.0177178 ) #0.0001 ) #999 ) #930.267 ) #0.00732462 , (((Sternotherus_carinatus #0.019849 , Kinosternon_leucostomum #498.893 ) #0.0457883 , (Macrochelys_temminckii #0.00587644 , Chelydra_serpentina #0.0514226 ) #0.0988485 ) #0.0001 , (((Platysternon_megacephalum #0.0352576 , (Chrysemys_picta #0.0944455 , (Trachemys_scripta #0.057726 , Malaclemys_terrapin #0.356828 ) #0.0001 ) #0.0286823 ) #0.0001 , (((((Astrochelys_yniphora #0.0193403 , Aldabrachelys_gigantea #0.0388683 ) #0.0001 , Psammobates_pardalis #0.0345534 ) #0.0203573 , ((((Indotestudo_forstenii #0.127264 , Indotestudo_elongata #0.0177588 ) #0.191099 , Testudo_horsfieldii #0.0463882 ) #0.0001 , (Testudo_graeca #0.0522747 , (Testudo_marginata #0.0673186 , Testudo_kleinmanni #0.0578767 ) #0.0001 ) #0.0431314 ) #0.0332996 , Malacochersus_tornieri #0.221446 ) #0.0137351 ) #0.0001 , (Manouria_impressa #0.045806 , Manouria_emys #0.0286712 ) #0.0498544 ) #0.0001 , ((Batagur_trivittata #0.0158782 , Geoemyda_spengleri #0.0318829 ) #999 , ((((Notochelys_platynota #0.181119 , (Cyclemys_fusca #0.0001 , ((Cyclemys_tcheponensis #0.0001 , Cyclemys_oldhamii #0.0001 ) #0.277901 , (Cyclemys_dentata #0.0664471 , (Cyclemys_pulchristriata #0.0001 , Cyclemys_atripons #0.0001 ) #0.0001 ) #0.0206897 ) #0.0001 ) #0.0001 ) #0.0001 , (Heosemys_annandalii #0.0233446 , (Heosemys_grandis #0.0295536 , Heosemys_depressa #0.0371129 ) #0.0340321 ) #0.0001 ) #184.648 , (Sacalia_quadriocellata #0.038226 , Sacalia_bealei #0.0093611 ) #0.0195716 ) #0.0456702 , (((((Cuora_flavomarginata #0.0309582 , (Cuora_trifasciata #0.0462533 , (Cuora_pani #42.4603 , Cuora_aurocapitata #2.59413 ) #0.0298266 ) #0.0859811 ) #0.0001 , (Cuora_mccordi #0.0001 , (Cuora_galbinifrons #0.0001 , (Cuora_picturata #999 , Cuora_bourreti #0.0001 ) #0.152919 ) #0.0210566 ) #0.0001 ) #0.0001 , Cuora_mouhotii #0.0755578 ) #0.0001 , Cuora_amboinensis #0.0001 ) #0.0001 , ((Mauremys_leprosa #0.0125626 , (((Mauremys_japonica #0.0170183 , Mauremys_sinensis #0.0386795 ) #0.0001 , ((Mauremys_reevesii #469.746 , NC_015101.1 #437.818 ) #0.0593214 , Mauremys_nigricans #0.0545027 ) #0.0343854 ) #0.0423426 , (Mauremys_rivulata #0.0001 , Mauremys_caspica #0.0001 ) #0.0578941 ) #0.0001 ) #999 , (Mauremys_mutica #0.0001 , Mauremys_annamensis #0.0188205 ) #0.0001 ) #0.0001 ) #0.0529424 ) #0.0303425 ) #999 ) #0.0246158 ) #0.0001 , (Dermochelys_coriacea #0.00899583 , ((Eretmochelys_imbricata #0.0549357 , (Caretta_caretta #0.0167148 , (Lepidochelys_olivacea #0.0001 , Lepidochelys_kempii #0.025327 ) #0.0138599 ) #0.0270967 ) #0.0001 , (Chelonia_mydas #0.0001 , Natator_depressa #0.012306 ) #0.0218813 ) #0.0001 ) #0.02458 ) #0.107564 ) #0.0001 ) #0.0001 ) #0.00648208 );

**COX3**

**dS tree:**

((((((Anolis_carolinensis: 64.019722, Iguana_iguana: 2.144201): 0.000173, Anguis_fragilis: 3.268967): 0.000081, Lacerta_viridis: 23.989743): 0.000135, Smaug_warreni: 1.228173): 0.698265, (Rena_humilis: 6.140489, ((Python_regius: 1.747664, Boa_constrictor: 11.581410): 0.000481, (Protobothrops_flavoviridis: 1.123552, Dinodon_semicarinatus: 1.976647): 2.001551): 1.456243): 0.000429): 0.000138, ((Rhea_americana: 1.646210, (Gallus_gallus: 2.393785, (Columba_livia: 1.726609, (Grus_leucogeranus: 2.821161, (Falco_peregrinus: 1.457901, Pelecanus_occidentalis: 0.921493): 0.010350): 0.000054): 0.203513): 0.000005): 1.525460, ((Alligator_mississippiensis: 0.727728, Caiman_crocodylus: 0.580919): 0.742560, (Crocodylus_porosus: 0.381527, Gavialis_gangeticus: 1.431636): 2.509661): 0.163077): 0.180207, (((Podocnemis_unifilis: 5.144586, (Pelusios_castaneus: 0.000203, Pelomedusa_subrufa: 1.320995): 3.269776): 0.000204, ((Chelus_fimbriata: 1.301358, (Platemys_platycephala: 0.704555, Mesoclemmys_hogei: 0.140969): 0.394629): 0.572901, (((Elusor_macrurus: 0.000116, (Myuchelys_bellii: 0.166358, Emydura_subglobosa: 0.285720): 0.371016): 0.737838, (Chelodina_parkeri: 0.316863, (Chelodina_rugosa: 0.371527, (Chelodina_pritchardi: 0.159445, (Chelodina_mccordi: 0.038897, (Chelodina_longicollis: 0.005048, Chelodina_expansa: 0.005443): 0.033669): 0.197136): 0.053873): 0.000051): 2.108686): 0.000086, Pseudemydura_umbrina: 1.994983): 0.000241): 25.331145): 0.000217, ((Carettochelys_insculpta: 64.014752, ((Lissemys_scutata: 0.000115, Lissemys_punctata: 0.466217): 5.545256, ((Trionyx_triunguis: 0.853572, (Pelochelys_cantorii: 0.499967, Chitra_indica: 0.245304): 0.000005): 0.306916, ((Rafetus_swinhoei: 0.310677, (Apalone_spinifera: 0.110846, Apalone_ferox: 0.295936): 0.386159): 0.000015, ((Amyda_cartilaginea: 0.525801, Dogania_subplana: 0.445751): 0.224392, (Palea_steindachneri: 0.414553, Pelodiscus_sinensis: 0.296510): 0.025442): 0.036983): 0.076869): 0.000062): 0.000096): 0.000040, (((Sternotherus_carinatus: 0.103194, Kinosternon_leucostomum: 0.304272): 0.764023, (Macrochelys_temminckii: 0.251188, Chelydra_serpentina: 0.288992): 0.374542): 0.003697, (((Platysternon_megacephalum: 0.630415, (Chrysemys_picta: 0.230021, (Trachemys_scripta: 0.056906, Malaclemys_terrapin: 0.124588): 0.097647): 0.187831): 0.044337, (((((Astrochelys_yniphora: 0.188060, Aldabrachelys_gigantea: 0.243472): 0.080821, Psammobates_pardalis: 0.304887): 0.161266, ((((Indotestudo_forstenii: 0.057448, Indotestudo_elongata: 0.071332): 0.107928, Testudo_horsfieldii: 0.216279): 0.049783, (Testudo_graeca: 0.180838, (Testudo_marginata: 0.044866, Testudo_kleinmanni: 0.103020): 0.056575): 0.172282): 0.009455, Malacochersus_tornieri: 0.283616): 0.074312): 0.106239, (Manouria_impressa: 0.155127, Manouria_emys: 0.215803): 0.193159): 0.000088, ((Batagur_trivittata: 0.539964, Geoemyda_spengleri: 0.362527): 0.067411, ((((Notochelys_platynota: 0.323496, (Cyclemys_fusca: 0.154069, ((Cyclemys_tcheponensis: 0.018701, Cyclemys_oldhamii: 0.000001): 0.113699, (Cyclemys_dentata: 0.039324, (Cyclemys_pulchristriata: 0.000005, Cyclemys_atripons: 0.014461): 0.048969): 0.127261): 0.053236): 0.070633): 0.040509, (Heosemys_annandalii: 0.129185, (Heosemys_grandis: 0.103790, Heosemys_depressa: 0.101951): 0.063812): 0.148590): 0.031665, (Sacalia_quadriocellata: 0.133697, Sacalia_bealei: 0.177495): 0.067435): 0.069597, (((((Cuora_flavomarginata: 0.138211, (Cuora_trifasciata: 0.046381, (Cuora_pani: 0.000000, Cuora_aurocapitata: 0.000000): 0.036396): 0.024022): 0.005198, (Cuora_mccordi: 0.010571, (Cuora_galbinifrons: 0.118213, (Cuora_picturata: 0.000000, Cuora_bourreti: 0.000000): 0.051378): 0.151227): 0.012202): 0.072412, Cuora_mouhotii: 0.168011): 0.080018, Cuora_amboinensis: 0.165690): 0.040195, ((Mauremys_leprosa: 0.220186, (((Mauremys_japonica: 0.068021, Mauremys_sinensis: 0.039929): 0.012092, ((Mauremys_reevesii: 0.005408, NC_015101.1: 0.006273): 0.059241, Mauremys_nigricans: 0.066227): 0.007470): 0.049962, (Mauremys_rivulata: 0.005056, Mauremys_caspica: 0.000005): 0.096336): 0.003785): 0.000000, (Mauremys_mutica: 0.136408, Mauremys_annamensis: 0.104060): 0.122872): 0.079640): 0.103825): 0.091085): 0.079161): 0.211685): 0.086397, (Dermochelys_coriacea: 0.378114, ((Eretmochelys_imbricata: 0.173043, (Caretta_caretta: 0.133345, (Lepidochelys_olivacea: 0.029599, Lepidochelys_kempii: 0.014040): 0.138284): 0.051716): 0.180155, (Chelonia_mydas: 0.253380, Natator_depressa: 0.121294): 0.060446): 0.130435): 0.221873): 0.128533): 0.866548): 64.143187): 0.000192);

**dN tree:**

((((((Anolis_carolinensis: 0.054403, Iguana_iguana: 0.022569): 0.020530, Anguis_fragilis: 0.069523): 0.007069, Lacerta_viridis: 0.062128): 0.010505, Smaug_warreni: 0.028271): 0.025743, (Rena_humilis: 0.164768, ((Python_regius: 0.019663, Boa_constrictor: 0.041077): 0.027489, (Protobothrops_flavoviridis: 0.031665, Dinodon_semicarinatus: 0.046527): 0.009787): 0.061806): 0.075602): 0.026888, ((Rhea_americana: 0.014062, (Gallus_gallus: 0.018095, (Columba_livia: 0.019028, (Grus_leucogeranus: 0.105522, (Falco_peregrinus: 0.041087, Pelecanus_occidentalis: 0.006985): 0.007303): 0.002004): 0.010835): 0.000000): 0.064278, ((Alligator_mississippiensis: 0.019868, Caiman_crocodylus: 0.040022): 0.019355, (Crocodylus_porosus: 0.026255, Gavialis_gangeticus: 0.009024): 0.019312): 0.041076): 0.010103, (((Podocnemis_unifilis: 0.031929, (Pelusios_castaneus: 0.024672, Pelomedusa_subrufa: 0.013729): 0.065255): 0.024916, ((Chelus_fimbriata: 0.036645, (Platemys_platycephala: 0.006810, Mesoclemmys_hogei: 0.010838): 0.007173): 0.005627, (((Elusor_macrurus: 0.013094, (Myuchelys_bellii: 0.009517, Emydura_subglobosa: 0.002607): 0.005226): 0.012944, (Chelodina_parkeri: 0.004687, (Chelodina_rugosa: 0.007755, (Chelodina_pritchardi: 0.004216, (Chelodina_mccordi: 0.000004, (Chelodina_longicollis: 0.000001, Chelodina_expansa: 0.000001): 0.003473): 0.006309): 0.005555): 0.003342): 0.016401): 0.001977, Pseudemydura_umbrina: 0.022218): 0.006879): 0.035208): 0.021870, ((Carettochelys_insculpta: 0.056147, ((Lissemys_scutata: 0.012985, Lissemys_punctata: 0.007121): 0.030801, ((Trionyx_triunguis: 0.018191, (Pelochelys_cantorii: 0.012350, Chitra_indica: 0.011378): 0.000000): 0.002010, ((Rafetus_swinhoei: 0.005181, (Apalone_spinifera: 0.004178, Apalone_ferox: 0.006072): 0.013485): 0.002234, ((Amyda_cartilaginea: 0.008666, Dogania_subplana: 0.021049): 0.000022, (Palea_steindachneri: 0.008656, Pelodiscus_sinensis: 0.008655): 0.000003): 0.007612): 0.004922): 0.007589): 0.012798): 0.002995, (((Sternotherus_carinatus: 0.010587, Kinosternon_leucostomum: 0.005237): 0.030146, (Macrochelys_temminckii: 0.011641, Chelydra_serpentina: 0.011460): 0.006986): 0.000000, (((Platysternon_megacephalum: 0.035618, (Chrysemys_picta: 0.008131, (Trachemys_scripta: 0.002504, Malaclemys_terrapin: 0.011697): 0.003195): 0.006481): 0.005288, (((((Astrochelys_yniphora: 0.010613, Aldabrachelys_gigantea: 0.003471): 0.003231, Psammobates_pardalis: 0.008876): 0.000016, ((((Indotestudo_forstenii: 0.002748, Indotestudo_elongata: 0.011115): 0.002330, Testudo_horsfieldii: 0.012822): 0.000718, (Testudo_graeca: 0.010290, (Testudo_marginata: 0.002007, Testudo_kleinmanni: 0.003150): 0.004520): 0.001379): 0.001505, Malacochersus_tornieri: 0.018780): 0.001874): 0.000011, (Manouria_impressa: 0.010923, Manouria_emys: 0.012303): 0.006197): 0.004985, ((Batagur_trivittata: 0.008923, Geoemyda_spengleri: 0.018220): 0.000007, ((((Notochelys_platynota: 0.014435, (Cyclemys_fusca: 0.005561, ((Cyclemys_tcheponensis: 0.000002, Cyclemys_oldhamii: 0.000002): 0.003637, (Cyclemys_dentata: 0.001822, (Cyclemys_pulchristriata: 0.000000, Cyclemys_atripons: 0.000001): 0.003628): 0.001791): 0.000005): 0.001771): 0.000004, (Heosemys_annandalii: 0.007309, (Heosemys_grandis: 0.007108, Heosemys_depressa: 0.003532): 0.001757): 0.000015): 0.000003, (Sacalia_quadriocellata: 0.005374, Sacalia_bealei: 0.007238): 0.001784): 0.005316, (((((Cuora_flavomarginata: 0.005260, (Cuora_trifasciata: 0.003490, (Cuora_pani: 0.000002, Cuora_aurocapitata: 0.000002): 0.003511): 0.001752): 0.000001, (Cuora_mccordi: 0.000001, (Cuora_galbinifrons: 0.003508, (Cuora_picturata: 0.000002, Cuora_bourreti: 0.000002): 0.003492): 0.003506): 0.000001): 0.003518, Cuora_mouhotii: 0.008885): 0.000008, Cuora_amboinensis: 0.007023): 0.000004, ((Mauremys_leprosa: 0.005183, (((Mauremys_japonica: 0.003444, Mauremys_sinensis: 0.000004): 0.000001, ((Mauremys_reevesii: 0.000001, NC_015101.1: 0.004625): 0.005164, Mauremys_nigricans: 0.003448): 0.001714): 0.003449, (Mauremys_rivulata: 0.000001, Mauremys_caspica: 0.000000): 0.001716): 0.001718): 0.000002, (Mauremys_mutica: 0.003454, Mauremys_annamensis: 0.005188): 0.000012): 0.000008): 0.001736): 0.001751): 0.001751): 0.005263): 0.001744, (Dermochelys_coriacea: 0.012097, ((Eretmochelys_imbricata: 0.000017, (Caretta_caretta: 0.003375, (Lepidochelys_olivacea: 0.000003, Lepidochelys_kempii: 0.001679): 0.007064): 0.005068): 0.001692, (Chelonia_mydas: 0.001681, Natator_depressa: 0.003420): 0.003407): 0.001750): 0.000022): 0.000825): 0.012008): 0.011089): 0.015754);

**w ratios as labels for TreeView:**

((((((Anolis_carolinensis #0.000849788 , Iguana_iguana #0.0105256 ) #118.583 , Anguis_fragilis #0.0212676 ) #87.6059 , Lacerta_viridis #0.00258976 ) #78.0579 , Smaug_warreni #0.0230189 ) #0.0368669 , (Rena_humilis #0.0268331 , ((Python_regius #0.0112512 , Boa_constrictor #0.00354681 ) #57.2075 , (Protobothrops_flavoviridis #0.0281831 , Dinodon_semicarinatus #0.0235385 ) #0.00488968 ) #0.0424418 ) #176.207 ) #194.914 , ((Rhea_americana #0.00854224 , (Gallus_gallus #0.00755911 , (Columba_livia #0.0110202 , (Grus_leucogeranus #0.0374036 , (Falco_peregrinus #0.0281826 , Pelecanus_occidentalis #0.00758001 ) #0.705582 ) #37.1866 ) #0.0532405 ) #0.0001 ) #0.0421371 , ((Alligator_mississippiensis #0.0273017 , Caiman_crocodylus #0.068894 ) #0.0260654 , (Crocodylus_porosus #0.0688149 , Gavialis_gangeticus #0.00630329 ) #0.00769526 ) #0.251883 ) #0.0560627 , (((Podocnemis_unifilis #0.00620635 , (Pelusios_castaneus #121.792 , Pelomedusa_subrufa #0.0103927 ) #0.019957 ) #122.374 , ((Chelus_fimbriata #0.0281587 , (Platemys_platycephala #0.0096654 , Mesoclemmys_hogei #0.0768822 ) #0.0181753 ) #0.00982116 , (((Elusor_macrurus #112.403 , (Myuchelys_bellii #0.0572086 , Emydura_subglobosa #0.00912416 ) #0.0140849 ) #0.0175425 , (Chelodina_parkeri #0.0147916 , (Chelodina_rugosa #0.0208727 , (Chelodina_pritchardi #0.0264396 , (Chelodina_mccordi #0.0001 , (Chelodina_longicollis #0.0001 , Chelodina_expansa #0.0001 ) #0.103159 ) #0.0320016 ) #0.103119 ) #65.2823 ) #0.00777799 ) #23.0919 , Pseudemydura_umbrina #0.0111367 ) #28.5707 ) #0.0013899 ) #100.749 , ((Carettochelys_insculpta #0.000877091 , ((Lissemys_scutata #112.475 , Lissemys_punctata #0.0152745 ) #0.00555452 , ((Trionyx_triunguis #0.021312 , (Pelochelys_cantorii #0.0247022 , Chitra_indica #0.0463824 ) #0.0001 ) #0.0065491 , ((Rafetus_swinhoei #0.0166757 , (Apalone_spinifera #0.0376956 , Apalone_ferox #0.0205188 ) #0.0349205 ) #145.711 , ((Amyda_cartilaginea #0.0164808 , Dogania_subplana #0.0472203 ) #0.0001 , (Palea_steindachneri #0.0208815 , Pelodiscus_sinensis #0.0291879 ) #0.0001 ) #0.205831 ) #0.0640338 ) #123.184 ) #133.704 ) #74.605 , (((Sternotherus_carinatus #0.102593 , Kinosternon_leucostomum #0.0172107 ) #0.0394575 , (Macrochelys_temminckii #0.0463437 , Chelydra_serpentina #0.0396562 ) #0.018652 ) #0.0001 , (((Platysternon_megacephalum #0.0564998 , (Chrysemys_picta #0.0353487 , (Trachemys_scripta #0.0440084 , Malaclemys_terrapin #0.0938826 ) #0.0327196 ) #0.0345019 ) #0.119271 , (((((Astrochelys_yniphora #0.0564331 , Aldabrachelys_gigantea #0.0142565 ) #0.0399781 , Psammobates_pardalis #0.0291138 ) #0.0001 , ((((Indotestudo_forstenii #0.0478327 , Indotestudo_elongata #0.155815 ) #0.0215898 , Testudo_horsfieldii #0.0592829 ) #0.01442 , (Testudo_graeca #0.0569003 , (Testudo_marginata #0.0447416 , Testudo_kleinmanni #0.0305766 ) #0.0798962 ) #0.0080024 ) #0.159138 , Malacochersus_tornieri #0.0662161 ) #0.025214 ) #0.0001 , (Manouria_impressa #0.0704119 , Manouria_emys #0.0570118 ) #0.0320809 ) #56.8908 , ((Batagur_trivittata #0.0165249 , Geoemyda_spengleri #0.0502594 ) #0.0001 , ((((Notochelys_platynota #0.0446217 , (Cyclemys_fusca #0.0360952 , ((Cyclemys_tcheponensis #0.0001 , Cyclemys_oldhamii #2.21443 ) #0.0319917 , (Cyclemys_dentata #0.046336 , (Cyclemys_pulchristriata #0.0001 , Cyclemys_atripons #0.0001 ) #0.0740773 ) #0.0140743 ) #0.0001 ) #0.0250688 ) #0.0001 , (Heosemys_annandalii #0.0565797 , (Heosemys_grandis #0.0684845 , Heosemys_depressa #0.0346396 ) #0.0275276 ) #0.0001 ) #0.0001 , (Sacalia_quadriocellata #0.040196 , Sacalia_bealei #0.0407769 ) #0.0264617 ) #0.0763846 , (((((Cuora_flavomarginata #0.0380574 , (Cuora_trifasciata #0.0752365 , (Cuora_pani #4.20866 , Cuora_aurocapitata #5.5673 ) #0.0964536 ) #0.0729302 ) #0.0001 , (Cuora_mccordi #0.0001 , (Cuora_galbinifrons #0.0296732 , (Cuora_picturata #3.52516 , Cuora_bourreti #10.1231 ) #0.0679605 ) #0.0231853 ) #0.0001 ) #0.0485772 , Cuora_mouhotii #0.052883 ) #0.0001 , Cuora_amboinensis #0.0423868 ) #0.0001 , ((Mauremys_leprosa #0.0235385 , (((Mauremys_japonica #0.0506351 , Mauremys_sinensis #0.0001 ) #0.0001 , ((Mauremys_reevesii #0.0001 , NC_015101.1 #0.737274 ) #0.0871676 , Mauremys_nigricans #0.0520669 ) #0.229472 ) #0.0690403 , (Mauremys_rivulata #0.0001 , Mauremys_caspica #0.0001 ) #0.0178077 ) #0.453882 ) #9.94454 , (Mauremys_mutica #0.0253221 , Mauremys_annamensis #0.049854 ) #0.0001 ) #0.0001 ) #0.0167237 ) #0.0192275 ) #0.0221178 ) #0.0248634 ) #0.0201853 , (Dermochelys_coriacea #0.0319924 , ((Eretmochelys_imbricata #0.0001 , (Caretta_caretta #0.0253104 , (Lepidochelys_olivacea #0.0001 , Lepidochelys_kempii #0.119557 ) #0.0510858 ) #0.0979912 ) #0.00939111 , (Chelonia_mydas #0.00663523 , Natator_depressa #0.0281993 ) #0.0563592 ) #0.0134158 ) #0.0001 ) #0.00642034 ) #0.0138577 ) #0.000172883 ) #82.0701 );

**CYTB**

**dS tree:**

((((((Anolis_carolinensis: 2.127152, Iguana_iguana: 2.458628): 0.895966, Anguis_fragilis: 3.741316): 0.000206, Lacerta_viridis: 2.141167): 0.000267, Smaug_warreni: 3.530476): 0.141809, (Rena_humilis: 11.026290, ((Python_regius: 11.934551, Boa_constrictor: 64.232969): 0.001224, (Protobothrops_flavoviridis: 4.304996, Dinodon_semicarinatus: 64.210963): 0.001053): 0.003101): 0.001584): 0.000024, ((Rhea_americana: 1.051615, (Gallus_gallus: 1.668120, (Columba_livia: 1.114329, (Grus_leucogeranus: 0.602137, (Falco_peregrinus: 2.208278, Pelecanus_occidentalis: 0.617308): 0.167736): 0.205403): 0.544131): 0.082897): 1.820116, ((Alligator_mississippiensis: 0.567231, Caiman_crocodylus: 1.217878): 1.131955, (Crocodylus_porosus: 3.027397, Gavialis_gangeticus: 0.923627): 0.976657): 1.343542): 0.000692, (((Podocnemis_unifilis: 5.055728, (Pelusios_castaneus: 0.462137, Pelomedusa_subrufa: 0.834927): 2.072250): 0.000675, ((Chelus_fimbriata: 1.200053, (Platemys_platycephala: 0.616243, Mesoclemmys_hogei: 0.390404): 0.338318): 0.000480, (((Elusor_macrurus: 0.236749, (Myuchelys_bellii: 0.148323, Emydura_subglobosa: 0.294627): 0.310467): 0.787651, (Chelodina_parkeri: 0.684740, (Chelodina_rugosa: 0.516356, (Chelodina_pritchardi: 0.143788, (Chelodina_mccordi: 0.086984, (Chelodina_longicollis: 0.000000, Chelodina_expansa: 0.006982): 0.016932): 0.044694): 0.073674): 0.000001): 0.830253): 0.000366, Pseudemydura_umbrina: 1.520041): 1.526005): 3.265294): 0.000461, ((Carettochelys_insculpta: 4.150419, ((Lissemys_scutata: 0.670347, Lissemys_punctata: 0.000626): 1.832220, ((Trionyx_triunguis: 1.039979, (Pelochelys_cantorii: 0.299973, Chitra_indica: 0.553280): 0.241992): 0.317393, ((Rafetus_swinhoei: 0.351455, (Apalone_spinifera: 0.223119, Apalone_ferox: 0.127284): 0.448869): 0.071411, ((Amyda_cartilaginea: 0.468973, Dogania_subplana: 0.526212): 0.182813, (Palea_steindachneri: 0.331029, Pelodiscus_sinensis: 0.696207): 0.000099): 0.330077): 0.000155): 1.492752): 0.886083): 0.000835, (((Sternotherus_carinatus: 0.000296, Kinosternon_leucostomum: 0.470366): 1.980602, (Macrochelys_temminckii: 0.468394, Chelydra_serpentina: 0.401224): 0.358070): 0.000157, (((Platysternon_megacephalum: 0.868535, (Chrysemys_picta: 0.202908, (Trachemys_scripta: 0.070530, Malaclemys_terrapin: 0.102156): 0.127430): 0.555156): 0.062720, (((((Astrochelys_yniphora: 0.247105, Aldabrachelys_gigantea: 0.419838): 0.122945, Psammobates_pardalis: 0.237923): 0.087540, ((((Indotestudo_forstenii: 0.122363, Indotestudo_elongata: 0.179551): 0.199773, Testudo_horsfieldii: 0.230287): 0.000029, (Testudo_graeca: 0.281202, (Testudo_marginata: 0.130031, Testudo_kleinmanni: 0.126460): 0.031876): 0.160692): 0.021681, Malacochersus_tornieri: 0.373094): 0.170860): 0.178372, (Manouria_impressa: 0.264347, Manouria_emys: 0.131838): 0.269553): 0.119496, ((Batagur_trivittata: 0.817955, Geoemyda_spengleri: 0.410065): 0.220072, ((((Notochelys_platynota: 0.416078, (Cyclemys_fusca: 0.532546, ((Cyclemys_tcheponensis: 0.005613, Cyclemys_oldhamii: 0.021799): 0.135415, (Cyclemys_dentata: 0.084980, (Cyclemys_pulchristriata: 0.010706, Cyclemys_atripons: 0.025905): 0.051210): 0.101851): 0.061392): 0.057551): 0.009262, (Heosemys_annandalii: 0.230222, (Heosemys_grandis: 0.171686, Heosemys_depressa: 0.122434): 0.095369): 0.197427): 0.045011, (Sacalia_quadriocellata: 0.153137, Sacalia_bealei: 0.231064): 0.058668): 0.082759, (((((Cuora_flavomarginata: 0.148425, (Cuora_trifasciata: 0.048521, (Cuora_pani: 0.000023, Cuora_aurocapitata: 0.047448): 0.064245): 0.054832): 0.015065, (Cuora_mccordi: 0.147993, (Cuora_galbinifrons: 0.073414, (Cuora_picturata: 0.014655, Cuora_bourreti: 0.003124): 0.028012): 0.032226): 0.046579): 0.026692, Cuora_mouhotii: 0.116270): 0.000005, Cuora_amboinensis: 0.190974): 0.135175, ((Mauremys_leprosa: 0.176071, (((Mauremys_japonica: 0.115912, Mauremys_sinensis: 0.064641): 0.008806, ((Mauremys_reevesii: 0.012068, NC_015101.1: 0.005989): 0.078932, Mauremys_nigricans: 0.075094): 0.015492): 0.118653, (Mauremys_rivulata: 0.000031, Mauremys_caspica: 0.000001): 0.186680): 0.021031): 0.000005, (Mauremys_mutica: 0.187623, Mauremys_annamensis: 0.116492): 0.055804): 0.063508): 0.167962): 0.128634): 0.000044): 0.420955): 0.139918, (Dermochelys_coriacea: 1.022267, ((Eretmochelys_imbricata: 0.237576, (Caretta_caretta: 0.139332, (Lepidochelys_olivacea: 0.024369, Lepidochelys_kempii: 0.022489): 0.154092): 0.054360): 0.176494, (Chelonia_mydas: 0.256667, Natator_depressa: 0.259300): 0.022906): 0.132251): 0.610723): 0.027096): 1.898110): 1.996993): 0.000810);

**dN tree:**

((((((Anolis_carolinensis: 0.071833, Iguana_iguana: 0.047669): 0.006564, Anguis_fragilis: 0.138460): 0.004773, Lacerta_viridis: 0.067974): 0.009974, Smaug_warreni: 0.102441): 0.019320, (Rena_humilis: 0.119681, ((Python_regius: 0.042398, Boa_constrictor: 0.047673): 0.016421, (Protobothrops_flavoviridis: 0.059052, Dinodon_semicarinatus: 0.055362): 0.030526): 0.158922): 0.120209): 0.000204, ((Rhea_americana: 0.027728, (Gallus_gallus: 0.019794, (Columba_livia: 0.014302, (Grus_leucogeranus: 0.019692, (Falco_peregrinus: 0.027725, Pelecanus_occidentalis: 0.017460): 0.006194): 0.002542): 0.023384): 0.002983): 0.047660, ((Alligator_mississippiensis: 0.045860, Caiman_crocodylus: 0.054921): 0.043858, (Crocodylus_porosus: 0.061019, Gavialis_gangeticus: 0.027381): 0.042718): 0.134891): 0.026760, (((Podocnemis_unifilis: 0.063524, (Pelusios_castaneus: 0.032704, Pelomedusa_subrufa: 0.051176): 0.042377): 0.023707, ((Chelus_fimbriata: 0.049562, (Platemys_platycephala: 0.024390, Mesoclemmys_hogei: 0.018114): 0.018292): 0.011411, (((Elusor_macrurus: 0.014472, (Myuchelys_bellii: 0.009676, Emydura_subglobosa: 0.011744): 0.006173): 0.025114, (Chelodina_parkeri: 0.018662, (Chelodina_rugosa: 0.011385, (Chelodina_pritchardi: 0.007970, (Chelodina_mccordi: 0.002580, (Chelodina_longicollis: 0.000002, Chelodina_expansa: 0.005121): 0.006405): 0.005156): 0.009316): 0.000001): 0.046261): 0.007107, Pseudemydura_umbrina: 0.046689): 0.015184): 0.027625): 0.020292, ((Carettochelys_insculpta: 0.053859, ((Lissemys_scutata: 0.013845, Lissemys_punctata: 0.007553): 0.046754, ((Trionyx_triunguis: 0.024956, (Pelochelys_cantorii: 0.014097, Chitra_indica: 0.014163): 0.003548): 0.007624, ((Rafetus_swinhoei: 0.009135, (Apalone_spinifera: 0.008591, Apalone_ferox: 0.004042): 0.017638): 0.002481, ((Amyda_cartilaginea: 0.020251, Dogania_subplana: 0.018930): 0.011883, (Palea_steindachneri: 0.016370, Pelodiscus_sinensis: 0.013790): 0.005457): 0.000033): 0.005429): 0.016864): 0.028491): 0.016339, (((Sternotherus_carinatus: 0.009973, Kinosternon_leucostomum: 0.009419): 0.049921, (Macrochelys_temminckii: 0.013036, Chelydra_serpentina: 0.023034): 0.018143): 0.004459, (((Platysternon_megacephalum: 0.041416, (Chrysemys_picta: 0.008262, (Trachemys_scripta: 0.008710, Malaclemys_terrapin: 0.002993): 0.006418): 0.018278): 0.003044, (((((Astrochelys_yniphora: 0.016148, Aldabrachelys_gigantea: 0.013301): 0.006194, Psammobates_pardalis: 0.017401): 0.004191, ((((Indotestudo_forstenii: 0.008940, Indotestudo_elongata: 0.002545): 0.015773, Testudo_horsfieldii: 0.018696): 0.000917, (Testudo_graeca: 0.016788, (Testudo_marginata: 0.010987, Testudo_kleinmanni: 0.006609): 0.004608): 0.003196): 0.006090, Malacochersus_tornieri: 0.043287): 0.006185): 0.007259, (Manouria_impressa: 0.022470, Manouria_emys: 0.010941): 0.005987): 0.008996, ((Batagur_trivittata: 0.024669, Geoemyda_spengleri: 0.023347): 0.006988, ((((Notochelys_platynota: 0.031820, (Cyclemys_fusca: 0.052600, ((Cyclemys_tcheponensis: 0.003115, Cyclemys_oldhamii: 0.022350): 0.009567, (Cyclemys_dentata: 0.004378, (Cyclemys_pulchristriata: 0.002541, Cyclemys_atripons: 0.001247): 0.004551): 0.001358): 0.006867): 0.008346): 0.000001, (Heosemys_annandalii: 0.010706, (Heosemys_grandis: 0.012234, Heosemys_depressa: 0.014897): 0.002359): 0.004800): 0.003339, (Sacalia_quadriocellata: 0.020942, Sacalia_bealei: 0.009281): 0.003937): 0.000008, (((((Cuora_flavomarginata: 0.016281, (Cuora_trifasciata: 0.005315, (Cuora_pani: 0.001293, Cuora_aurocapitata: 0.049297): 0.007281): 0.005456): 0.002718, (Cuora_mccordi: 0.004119, (Cuora_galbinifrons: 0.001262, (Cuora_picturata: 0.000001, Cuora_bourreti: 0.003772): 0.006322): 0.002198): 0.001243): 0.000003, Cuora_mouhotii: 0.011696): 0.000000, Cuora_amboinensis: 0.014866): 0.000014, ((Mauremys_leprosa: 0.011286, (((Mauremys_japonica: 0.003766, Mauremys_sinensis: 0.003671): 0.001017, ((Mauremys_reevesii: 0.000001, NC_015101.1: 0.000001): 0.006110, Mauremys_nigricans: 0.001386): 0.001245): 0.001451, (Mauremys_rivulata: 0.002496, Mauremys_caspica: 0.000001): 0.004988): 0.000002): 0.000000, (Mauremys_mutica: 0.008857, Mauremys_annamensis: 0.008823): 0.002430): 0.005878): 0.005456): 0.002608): 0.009116): 0.009521): 0.000014, (Dermochelys_coriacea: 0.015898, ((Eretmochelys_imbricata: 0.002935, (Caretta_caretta: 0.009093, (Lepidochelys_olivacea: 0.002433, Lepidochelys_kempii: 0.003640): 0.004142): 0.000005): 0.004198, (Chelonia_mydas: 0.006871, Natator_depressa: 0.008042): 0.004048): 0.012131): 0.012855): 0.009866): 0.004978): 0.007452): 0.045340);

**w ratios as labels for TreeView:**

((((((Anolis_carolinensis #0.0337698 , Iguana_iguana #0.0193886 ) #0.00732589 , Anguis_fragilis #0.0370083 ) #23.2224 , Lacerta_viridis #0.0317464 ) #37.3228 , Smaug_warreni #0.0290162 ) #0.136238 , (Rena_humilis #0.0108542 , ((Python_regius #0.00355252 , Boa_constrictor #0.000742196 ) #13.4157 , (Protobothrops_flavoviridis #0.0137171 , Dinodon_semicarinatus #0.000862188 ) #28.9781 ) #51.2456 ) #75.9051 ) #8.58863 , ((Rhea_americana #0.0263674 , (Gallus_gallus #0.0118663 , (Columba_livia #0.0128349 , (Grus_leucogeranus #0.0327028 , (Falco_peregrinus #0.0125548 , Pelecanus_occidentalis #0.0282848 ) #0.0369259 ) #0.0123775 ) #0.0429758 ) #0.0359851 ) #0.0261851 , ((Alligator_mississippiensis #0.0808493 , Caiman_crocodylus #0.0450957 ) #0.0387453 , (Crocodylus_porosus #0.0201555 , Gavialis_gangeticus #0.0296454 ) #0.0437394 ) #0.100399 ) #38.6517 , (((Podocnemis_unifilis #0.0125647 , (Pelusios_castaneus #0.0707677 , Pelomedusa_subrufa #0.0612942 ) #0.0204495 ) #35.1408 , ((Chelus_fimbriata #0.0412998 , (Platemys_platycephala #0.0395793 , Mesoclemmys_hogei #0.0463986 ) #0.0540663 ) #23.7824 , (((Elusor_macrurus #0.0611283 , (Myuchelys_bellii #0.0652355 , Emydura_subglobosa #0.039862 ) #0.0198841 ) #0.0318848 , (Chelodina_parkeri #0.0272536 , (Chelodina_rugosa #0.0220485 , (Chelodina_pritchardi #0.0554286 , (Chelodina_mccordi #0.0296597 , (Chelodina_longicollis #3.49454 , Chelodina_expansa #0.733437 ) #0.378311 ) #0.115369 ) #0.126452 ) #1.33313 ) #0.0557192 ) #19.4389 , Pseudemydura_umbrina #0.0307156 ) #0.00995041 ) #0.00846006 ) #43.9795 , ((Carettochelys_insculpta #0.0129769 , ((Lissemys_scutata #0.0206536 , Lissemys_punctata #12.0737 ) #0.0255175 , ((Trionyx_triunguis #0.0239969 , (Pelochelys_cantorii #0.0469955 , Chitra_indica #0.025599 ) #0.0146603 ) #0.0240215 , ((Rafetus_swinhoei #0.025992 , (Apalone_spinifera #0.0385058 , Apalone_ferox #0.0317563 ) #0.0392934 ) #0.0347494 , ((Amyda_cartilaginea #0.0431811 , Dogania_subplana #0.0359746 ) #0.0649983 , (Palea_steindachneri #0.0494513 , Pelodiscus_sinensis #0.0198076 ) #55.2322 ) #0.0001 ) #35.0993 ) #0.0112975 ) #0.0321537 ) #19.5747 , (((Sternotherus_carinatus #33.6909 , Kinosternon_leucostomum #0.0200245 ) #0.025205 , (Macrochelys_temminckii #0.0278314 , Chelydra_serpentina #0.0574096 ) #0.0506683 ) #28.3209 , (((Platysternon_megacephalum #0.047685 , (Chrysemys_picta #0.0407167 , (Trachemys_scripta #0.123497 , Malaclemys_terrapin #0.0292959 ) #0.0503622 ) #0.032924 ) #0.048533 , (((((Astrochelys_yniphora #0.0653489 , Aldabrachelys_gigantea #0.031681 ) #0.0503813 , Psammobates_pardalis #0.0731376 ) #0.0478718 , ((((Indotestudo_forstenii #0.0730588 , Indotestudo_elongata #0.0141766 ) #0.0789533 , Testudo_horsfieldii #0.0811866 ) #31.2905 , (Testudo_graeca #0.0597001 , (Testudo_marginata #0.0844987 , Testudo_kleinmanni #0.0522633 ) #0.144571 ) #0.0198907 ) #0.280909 , Malacochersus_tornieri #0.116021 ) #0.0361966 ) #0.0406977 , (Manouria_impressa #0.0850019 , Manouria_emys #0.0829874 ) #0.0222123 ) #0.0752835 , ((Batagur_trivittata #0.0301594 , Geoemyda_spengleri #0.0569346 ) #0.0317518 , ((((Notochelys_platynota #0.0764764 , (Cyclemys_fusca #0.0987711 , ((Cyclemys_tcheponensis #0.555019 , Cyclemys_oldhamii #1.02529 ) #0.0706504 , (Cyclemys_dentata #0.0515196 , (Cyclemys_pulchristriata #0.23731 , Cyclemys_atripons #0.0481228 ) #0.08887 ) #0.0133313 ) #0.111853 ) #0.145021 ) #0.0001 , (Heosemys_annandalii #0.0465025 , (Heosemys_grandis #0.0712605 , Heosemys_depressa #0.12167 ) #0.024731 ) #0.0243148 ) #0.0741722 , (Sacalia_quadriocellata #0.136751 , Sacalia_bealei #0.0401662 ) #0.0671028 ) #0.0001 , (((((Cuora_flavomarginata #0.109693 , (Cuora_trifasciata #0.109545 , (Cuora_pani #55.3622 , Cuora_aurocapitata #1.03897 ) #0.113335 ) #0.0995036 ) #0.180399 , (Cuora_mccordi #0.0278302 , (Cuora_galbinifrons #0.0171882 , (Cuora_picturata #0.0001 , Cuora_bourreti #1.20773 ) #0.225696 ) #0.0681994 ) #0.0266886 ) #0.0001 , Cuora_mouhotii #0.10059 ) #0.0001 , Cuora_amboinensis #0.0778443 ) #0.0001 , ((Mauremys_leprosa #0.0640999 , (((Mauremys_japonica #0.0324913 , Mauremys_sinensis #0.056784 ) #0.115497 , ((Mauremys_reevesii #0.0001 , NC_015101.1 #0.0001 ) #0.0774052 , Mauremys_nigricans #0.0184514 ) #0.0803547 ) #0.0122313 , (Mauremys_rivulata #79.5816 , Mauremys_caspica #1.56227 ) #0.026721 ) #0.0001 ) #0.0001 , (Mauremys_mutica #0.0472051 , Mauremys_annamensis #0.075737 ) #0.0435522 ) #0.0925583 ) #0.0324837 ) #0.020275 ) #205.816 ) #0.0226171 ) #0.0001 , (Dermochelys_coriacea #0.0155513 , ((Eretmochelys_imbricata #0.0123556 , (Caretta_caretta #0.0652592 , (Lepidochelys_olivacea #0.09986 , Lepidochelys_kempii #0.161841 ) #0.026877 ) #0.0001 ) #0.023787 , (Chelonia_mydas #0.0267707 , Natator_depressa #0.0310153 ) #0.176703 ) #0.0917282 ) #0.0210482 ) #0.364108 ) #0.0026228 ) #0.0037316 ) #55.9621 );

**ND1**

**dS tree:**

((((((Anolis_carolinensis: 6.839134, Iguana_iguana: 3.575985): 0.001731, Anguis_fragilis: 3.547523): 0.001817, Lacerta_viridis: 4.010819): 0.099298, Smaug_warreni: 5.119787): 0.000434, (Rena_humilis: 5.907051, ((Python_regius: 2.485808, Boa_constrictor: 2.120313): 8.465834, (Protobothrops_flavoviridis: 5.544564, Dinodon_semicarinatus: 7.144499): 0.346778): 0.001670): 0.167431): 0.000681, ((Rhea_americana: 1.298384, (Gallus_gallus: 2.229714, (Columba_livia: 1.203536, (Grus_leucogeranus: 0.719853, (Falco_peregrinus: 1.035273, Pelecanus_occidentalis: 0.961635): 0.084552): 0.234215): 0.152609): 1.030475): 0.001714, ((Alligator_mississippiensis: 0.640025, Caiman_crocodylus: 1.249116): 0.414335, (Crocodylus_porosus: 1.355159, Gavialis_gangeticus: 1.342615): 0.539096): 1.345445): 0.371607, (((Podocnemis_unifilis: 8.953185, (Pelusios_castaneus: 0.530565, Pelomedusa_subrufa: 0.835635): 1.111653): 0.000448, ((Chelus_fimbriata: 1.289893, (Platemys_platycephala: 1.012484, Mesoclemmys_hogei: 0.291333): 0.442146): 0.257515, (((Elusor_macrurus: 0.495206, (Myuchelys_bellii: 0.070932, Emydura_subglobosa: 0.274365): 0.034652): 0.873705, (Chelodina_parkeri: 0.512120, (Chelodina_rugosa: 0.353712, (Chelodina_pritchardi: 0.091482, (Chelodina_mccordi: 0.076374, (Chelodina_longicollis: 0.000001, Chelodina_expansa: 0.003796): 0.045913): 0.037118): 0.338231): 0.000168): 1.655442): 0.038870, Pseudemydura_umbrina: 0.997538): 0.903334): 2.041940): 0.084491, ((Carettochelys_insculpta: 7.818628, ((Lissemys_scutata: 0.621159, Lissemys_punctata: 0.366155): 12.096748, ((Trionyx_triunguis: 0.426083, (Pelochelys_cantorii: 0.314659, Chitra_indica: 0.670802): 0.221380): 0.040135, ((Rafetus_swinhoei: 0.375749, (Apalone_spinifera: 0.143963, Apalone_ferox: 0.259374): 0.550662): 0.077641, ((Amyda_cartilaginea: 0.662825, Dogania_subplana: 0.454158): 0.235263, (Palea_steindachneri: 0.628239, Pelodiscus_sinensis: 0.612119): 0.000184): 0.055923): 0.641668): 2.478659): 0.000776): 0.000517, (((Sternotherus_carinatus: 0.329975, Kinosternon_leucostomum: 0.108007): 1.166254, (Macrochelys_temminckii: 0.562948, Chelydra_serpentina: 0.570115): 0.341504): 0.000179, (((Platysternon_megacephalum: 1.179370, (Chrysemys_picta: 0.394606, (Trachemys_scripta: 0.090107, Malaclemys_terrapin: 0.079810): 0.000352): 0.960352): 0.000002, (((((Astrochelys_yniphora: 0.303071, Aldabrachelys_gigantea: 0.329895): 0.066018, Psammobates_pardalis: 0.388686): 0.059460, ((((Indotestudo_forstenii: 0.064360, Indotestudo_elongata: 0.159022): 0.218129, Testudo_horsfieldii: 0.247962): 0.016883, (Testudo_graeca: 0.175258, (Testudo_marginata: 0.149081, Testudo_kleinmanni: 0.163175): 0.023941): 0.178384): 0.041921, Malacochersus_tornieri: 0.443773): 0.057971): 0.126102, (Manouria_impressa: 0.233450, Manouria_emys: 0.232503): 0.335842): 0.260783, ((Batagur_trivittata: 0.611547, Geoemyda_spengleri: 0.447343): 0.044295, ((((Notochelys_platynota: 0.288219, (Cyclemys_fusca: 0.209736, ((Cyclemys_tcheponensis: 0.011404, Cyclemys_oldhamii: 0.014578): 0.153784, (Cyclemys_dentata: 0.073102, (Cyclemys_pulchristriata: 0.015778, Cyclemys_atripons: 0.004671): 0.119969): 0.012501): 0.027954): 0.122447): 0.012129, (Heosemys_annandalii: 0.245797, (Heosemys_grandis: 0.109039, Heosemys_depressa: 0.194074): 0.042193): 0.374441): 0.020758, (Sacalia_quadriocellata: 0.135782, Sacalia_bealei: 0.157888): 0.070587): 0.117943, (((((Cuora_flavomarginata: 0.143100, (Cuora_trifasciata: 0.058793, (Cuora_pani: 0.004111, Cuora_aurocapitata: 0.000027): 0.035946): 0.036851): 0.033738, (Cuora_mccordi: 0.122442, (Cuora_galbinifrons: 0.102478, (Cuora_picturata: 0.000001, Cuora_bourreti: 0.000001): 0.054175): 0.030013): 0.018921): 0.011916, Cuora_mouhotii: 0.136933): 0.039518, Cuora_amboinensis: 0.269243): 0.035058, ((Mauremys_leprosa: 0.251926, (((Mauremys_japonica: 0.118061, Mauremys_sinensis: 0.069522): 0.004472, ((Mauremys_reevesii: 0.004541, NC_015101.1: 0.003903): 0.089989, Mauremys_nigricans: 0.079521): 0.000044): 0.145910, (Mauremys_rivulata: 0.003997, Mauremys_caspica: 0.000005): 0.170930): 0.000005): 0.016190, (Mauremys_mutica: 0.078238, Mauremys_annamensis: 0.089047): 0.062506): 0.108389): 0.096651): 0.017598): 0.000439): 0.760064): 0.000120, (Dermochelys_coriacea: 0.519512, ((Eretmochelys_imbricata: 0.261513, (Caretta_caretta: 0.112897, (Lepidochelys_olivacea: 0.053873, Lepidochelys_kempii: 0.042055): 0.192820): 0.083666): 0.146189, (Chelonia_mydas: 0.158869, Natator_depressa: 0.222374): 0.073173): 0.459471): 0.084300): 0.199322): 3.148783): 0.000393): 0.422168);

**dN tree:**

((((((Anolis_carolinensis: 0.085941, Iguana_iguana: 0.043443): 0.031088, Anguis_fragilis: 0.086024): 0.028806, Lacerta_viridis: 0.093826): 0.014530, Smaug_warreni: 0.083643): 0.010627, (Rena_humilis: 0.078466, ((Python_regius: 0.025491, Boa_constrictor: 0.041763): 0.012869, (Protobothrops_flavoviridis: 0.043200, Dinodon_semicarinatus: 0.030078): 0.021198): 0.074258): 0.089256): 0.035832, ((Rhea_americana: 0.034299, (Gallus_gallus: 0.048317, (Columba_livia: 0.019386, (Grus_leucogeranus: 0.033422, (Falco_peregrinus: 0.033905, Pelecanus_occidentalis: 0.011557): 0.008023): 0.000023): 0.004484): 0.003602): 0.088393, ((Alligator_mississippiensis: 0.045559, Caiman_crocodylus: 0.062992): 0.023168, (Crocodylus_porosus: 0.045619, Gavialis_gangeticus: 0.024921): 0.041969): 0.102121): 0.012950, (((Podocnemis_unifilis: 0.067285, (Pelusios_castaneus: 0.080093, Pelomedusa_subrufa: 0.043302): 0.066529): 0.008022, ((Chelus_fimbriata: 0.034716, (Platemys_platycephala: 0.018463, Mesoclemmys_hogei: 0.006673): 0.012435): 0.013832, (((Elusor_macrurus: 0.003638, (Myuchelys_bellii: 0.003789, Emydura_subglobosa: 0.003343): 0.008455): 0.017534, (Chelodina_parkeri: 0.015884, (Chelodina_rugosa: 0.010310, (Chelodina_pritchardi: 0.001475, (Chelodina_mccordi: 0.004406, (Chelodina_longicollis: 0.000002, Chelodina_expansa: 0.001483): 0.001471): 0.001470): 0.000034): 0.002986): 0.019300): 0.008938, Pseudemydura_umbrina: 0.030886): 0.018573): 0.025615): 0.013811, ((Carettochelys_insculpta: 0.107663, ((Lissemys_scutata: 0.021960, Lissemys_punctata: 0.026551): 0.039943, ((Trionyx_triunguis: 0.032437, (Pelochelys_cantorii: 0.022221, Chitra_indica: 0.020645): 0.001408): 0.004938, ((Rafetus_swinhoei: 0.013226, (Apalone_spinifera: 0.009373, Apalone_ferox: 0.010700): 0.010408): 0.005165, ((Amyda_cartilaginea: 0.040367, Dogania_subplana: 0.021051): 0.003574, (Palea_steindachneri: 0.056620, Pelodiscus_sinensis: 0.016177): 0.005875): 0.011231): 0.001626): 0.018959): 0.019402): 0.014899, (((Sternotherus_carinatus: 0.005491, Kinosternon_leucostomum: 0.001761): 0.061872, (Macrochelys_temminckii: 0.011334, Chelydra_serpentina: 0.014469): 0.012964): 0.002220, (((Platysternon_megacephalum: 0.031228, (Chrysemys_picta: 0.011322, (Trachemys_scripta: 0.002324, Malaclemys_terrapin: 0.003476): 0.003990): 0.007740): 0.000001, (((((Astrochelys_yniphora: 0.010981, Aldabrachelys_gigantea: 0.028109): 0.010090, Psammobates_pardalis: 0.034809): 0.009466, ((((Indotestudo_forstenii: 0.001416, Indotestudo_elongata: 0.001374): 0.008002, Testudo_horsfieldii: 0.019046): 0.004799, (Testudo_graeca: 0.021415, (Testudo_marginata: 0.002629, Testudo_kleinmanni: 0.018856): 0.004247): 0.003478): 0.001986, Malacochersus_tornieri: 0.065186): 0.016696): 0.006964, (Manouria_impressa: 0.008672, Manouria_emys: 0.004516): 0.004935): 0.007046, ((Batagur_trivittata: 0.051879, Geoemyda_spengleri: 0.017471): 0.005363, ((((Notochelys_platynota: 0.036823, (Cyclemys_fusca: 0.023586, ((Cyclemys_tcheponensis: 0.000001, Cyclemys_oldhamii: 0.000001): 0.004437, (Cyclemys_dentata: 0.008740, (Cyclemys_pulchristriata: 0.010350, Cyclemys_atripons: 0.000000): 0.008781): 0.000001): 0.002885): 0.002633): 0.000001, (Heosemys_annandalii: 0.004337, (Heosemys_grandis: 0.002937, Heosemys_depressa: 0.016040): 0.003379): 0.004590): 0.000002, (Sacalia_quadriocellata: 0.008795, Sacalia_bealei: 0.019080): 0.001494): 0.007439, (((((Cuora_flavomarginata: 0.010438, (Cuora_trifasciata: 0.004406, (Cuora_pani: 0.000000, Cuora_aurocapitata: 0.001454): 0.001465): 0.004318): 0.000003, (Cuora_mccordi: 0.013477, (Cuora_galbinifrons: 0.000010, (Cuora_picturata: 0.000001, Cuora_bourreti: 0.000002): 0.002919): 0.007181): 0.000002): 0.001475, Cuora_mouhotii: 0.008937): 0.002902, Cuora_amboinensis: 0.010693): 0.000004, ((Mauremys_leprosa: 0.007408, (((Mauremys_japonica: 0.008832, Mauremys_sinensis: 0.016222): 0.004247, ((Mauremys_reevesii: 0.000000, NC_015101.1: 0.001456): 0.001420, Mauremys_nigricans: 0.002931): 0.001457): 0.001441, (Mauremys_rivulata: 0.000000, Mauremys_caspica: 0.000000): 0.007323): 0.000000): 0.001491, (Mauremys_mutica: 0.000008, Mauremys_annamensis: 0.005953): 0.005771): 0.004411): 0.007025): 0.003326): 0.022315): 0.011651): 0.002847, (Dermochelys_coriacea: 0.011906, ((Eretmochelys_imbricata: 0.009914, (Caretta_caretta: 0.002793, (Lepidochelys_olivacea: 0.001452, Lepidochelys_kempii: 0.002893): 0.005734): 0.000008): 0.005030, (Chelonia_mydas: 0.002767, Natator_depressa: 0.008411): 0.002014): 0.004493): 0.007491): 0.006100): 0.020573): 0.006439): 0.057413);

**w ratios as labels for TreeView:**

((((((Anolis_carolinensis #0.012566 , Iguana_iguana #0.0121487 ) #17.9618 , Anguis_fragilis #0.0242491 ) #15.8527 , Lacerta_viridis #0.0233933 ) #0.146325 , Smaug_warreni #0.0163372 ) #24.4604 , (Rena_humilis #0.0132835 , ((Python_regius #0.0102545 , Boa_constrictor #0.0196965 ) #0.00152013 , (Protobothrops_flavoviridis #0.00779143 , Dinodon_semicarinatus #0.00420992 ) #0.0611276 ) #44.4575 ) #0.533092 ) #52.6286 , ((Rhea_americana #0.026417 , (Gallus_gallus #0.0216695 , (Columba_livia #0.0161072 , (Grus_leucogeranus #0.0464288 , (Falco_peregrinus #0.03275 , Pelecanus_occidentalis #0.0120177 ) #0.0948859 ) #0.0001 ) #0.0293834 ) #0.00349502 ) #51.5772 , ((Alligator_mississippiensis #0.0711831 , Caiman_crocodylus #0.0504291 ) #0.0559162 , (Crocodylus_porosus #0.0336629 , Gavialis_gangeticus #0.0185612 ) #0.0778512 ) #0.0759015 ) #0.0348492 , (((Podocnemis_unifilis #0.00751525 , (Pelusios_castaneus #0.150958 , Pelomedusa_subrufa #0.0518189 ) #0.0598467 ) #17.9029 , ((Chelus_fimbriata #0.0269141 , (Platemys_platycephala #0.0182353 , Mesoclemmys_hogei #0.0229038 ) #0.0281242 ) #0.0537139 , (((Elusor_macrurus #0.00734706 , (Myuchelys_bellii #0.0534239 , Emydura_subglobosa #0.012183 ) #0.243998 ) #0.0200682 , (Chelodina_parkeri #0.0310152 , (Chelodina_rugosa #0.0291468 , (Chelodina_pritchardi #0.0161272 , (Chelodina_mccordi #0.0576905 , (Chelodina_longicollis #2.28956 , Chelodina_expansa #0.390747 ) #0.0320369 ) #0.0396147 ) #0.0001 ) #17.8199 ) #0.0116587 ) #0.229947 , Pseudemydura_umbrina #0.0309625 ) #0.0205609 ) #0.0125444 ) #0.163457 , ((Carettochelys_insculpta #0.0137701 , ((Lissemys_scutata #0.0353537 , Lissemys_punctata #0.0725144 ) #0.003302 , ((Trionyx_triunguis #0.0761294 , (Pelochelys_cantorii #0.0706198 , Chitra_indica #0.030776 ) #0.00635877 ) #0.123044 , ((Rafetus_swinhoei #0.0351989 , (Apalone_spinifera #0.0651057 , Apalone_ferox #0.0412518 ) #0.0189013 ) #0.0665267 , ((Amyda_cartilaginea #0.0609018 , Dogania_subplana #0.0463511 ) #0.0151932 , (Palea_steindachneri #0.0901248 , Pelodiscus_sinensis #0.0264283 ) #31.9639 ) #0.200833 ) #0.00253407 ) #0.00764887 ) #25.0066 ) #28.8266 , (((Sternotherus_carinatus #0.0166397 , Kinosternon_leucostomum #0.0163056 ) #0.0530517 , (Macrochelys_temminckii #0.0201341 , Chelydra_serpentina #0.0253795 ) #0.0379603 ) #12.3783 , (((Platysternon_megacephalum #0.0264786 , (Chrysemys_picta #0.0286929 , (Trachemys_scripta #0.0257883 , Malaclemys_terrapin #0.0435477 ) #11.3227 ) #0.00805921 ) #0.476107 , (((((Astrochelys_yniphora #0.0362334 , Aldabrachelys_gigantea #0.0852067 ) #0.152832 , Psammobates_pardalis #0.0895563 ) #0.159204 , ((((Indotestudo_forstenii #0.0219948 , Indotestudo_elongata #0.00864105 ) #0.0366839 , Testudo_horsfieldii #0.0768108 ) #0.284217 , (Testudo_graeca #0.122191 , (Testudo_marginata #0.0176366 , Testudo_kleinmanni #0.115557 ) #0.177387 ) #0.0194966 ) #0.0473752 , Malacochersus_tornieri #0.146891 ) #0.288008 ) #0.0552285 , (Manouria_impressa #0.0371466 , Manouria_emys #0.0194222 ) #0.014695 ) #0.0270191 , ((Batagur_trivittata #0.0848322 , Geoemyda_spengleri #0.0390544 ) #0.121076 , ((((Notochelys_platynota #0.12776 , (Cyclemys_fusca #0.112455 , ((Cyclemys_tcheponensis #0.0001 , Cyclemys_oldhamii #0.0001 ) #0.0288515 , (Cyclemys_dentata #0.119555 , (Cyclemys_pulchristriata #0.655958 , Cyclemys_atripons #0.0001 ) #0.073194 ) #0.0001 ) #0.103199 ) #0.0215053 ) #0.0001 , (Heosemys_annandalii #0.0176447 , (Heosemys_grandis #0.0269333 , Heosemys_depressa #0.0826477 ) #0.0800799 ) #0.0122594 ) #0.0001 , (Sacalia_quadriocellata #0.0647697 , Sacalia_bealei #0.120848 ) #0.02117 ) #0.0630754 , (((((Cuora_flavomarginata #0.0729406 , (Cuora_trifasciata #0.0749482 , (Cuora_pani #0.0001 , Cuora_aurocapitata #52.9793 ) #0.0407658 ) #0.117166 ) #0.0001 , (Cuora_mccordi #0.110072 , (Cuora_galbinifrons #0.0001 , (Cuora_picturata #1.58535 , Cuora_bourreti #1.98498 ) #0.0538837 ) #0.239252 ) #0.0001 ) #0.12381 , Cuora_mouhotii #0.0652677 ) #0.0734267 , Cuora_amboinensis #0.0397145 ) #0.0001 , ((Mauremys_leprosa #0.029406 , (((Mauremys_japonica #0.0748123 , Mauremys_sinensis #0.233334 ) #0.949762 , ((Mauremys_reevesii #0.0001 , NC_015101.1 #0.373184 ) #0.0157846 , Mauremys_nigricans #0.0368587 ) #32.8304 ) #0.00987309 , (Mauremys_rivulata #0.0001 , Mauremys_caspica #0.0001 ) #0.0428437 ) #0.0001 ) #0.0921047 , (Mauremys_mutica #0.0001 , Mauremys_annamensis #0.0668471 ) #0.0923306 ) #0.0406991 ) #0.0726829 ) #0.188979 ) #50.8393 ) #0.0153295 ) #23.711 , (Dermochelys_coriacea #0.0229168 , ((Eretmochelys_imbricata #0.0379096 , (Caretta_caretta #0.0247376 , (Lepidochelys_olivacea #0.0269436 , Lepidochelys_kempii #0.0687945 ) #0.0297381 ) #0.0001 ) #0.034407 , (Chelonia_mydas #0.0174176 , Natator_depressa #0.0378224 ) #0.0275199 ) #0.00977755 ) #0.0888613 ) #0.0306041 ) #0.0065335 ) #16.4017 ) #0.135996 );

**ND2**

**dS tree:**

((((((Anolis_carolinensis: 2.759105, Iguana_iguana: 3.455866): 0.000599, Anguis_fragilis: 11.324787): 0.000419, Lacerta_viridis: 3.931385): 0.000485, Smaug_warreni: 5.595785): 0.000263, (Rena_humilis: 3.597137, ((Python_regius: 9.494970, Boa_constrictor: 63.613649): 0.002294, (Protobothrops_flavoviridis: 3.500232, Dinodon_semicarinatus: 4.351050): 0.270974): 0.001813): 0.142721): 0.000625, ((Rhea_americana: 1.708675, (Gallus_gallus: 1.166702, (Columba_livia: 1.116985, (Grus_leucogeranus: 0.557033, (Falco_peregrinus: 1.695931, Pelecanus_occidentalis: 0.792640): 0.013259): 0.128594): 0.551254): 0.291815): 0.985872, ((Alligator_mississippiensis: 3.541027, Caiman_crocodylus: 3.431512): 0.000314, (Crocodylus_porosus: 2.242945, Gavialis_gangeticus: 0.650582): 1.916741): 0.002318): 0.000483, (((Podocnemis_unifilis: 5.593784, (Pelusios_castaneus: 0.345820, Pelomedusa_subrufa: 0.609992): 2.210673): 0.002111, ((Chelus_fimbriata: 3.209128, (Platemys_platycephala: 0.448101, Mesoclemmys_hogei: 0.690103): 0.001413): 0.648497, (((Elusor_macrurus: 0.097111, (Myuchelys_bellii: 0.166240, Emydura_subglobosa: 0.247257): 0.206550): 0.549516, (Chelodina_parkeri: 0.355445, (Chelodina_rugosa: 0.297063, (Chelodina_pritchardi: 0.073734, (Chelodina_mccordi: 0.034676, (Chelodina_longicollis: 0.007386, Chelodina_expansa: 0.003434): 0.014504): 0.039887): 0.068227): 0.139738): 0.734266): 0.054038, Pseudemydura_umbrina: 0.927915): 0.303282): 0.160636): 0.084878, ((Carettochelys_insculpta: 39.064047, ((Lissemys_scutata: 0.230957, Lissemys_punctata: 0.369108): 63.635213, ((Trionyx_triunguis: 0.373755, (Pelochelys_cantorii: 0.453380, Chitra_indica: 0.622473): 0.072924): 0.193092, ((Rafetus_swinhoei: 0.407506, (Apalone_spinifera: 0.106361, Apalone_ferox: 0.195001): 0.625273): 0.000108, ((Amyda_cartilaginea: 0.363402, Dogania_subplana: 0.442037): 0.172616, (Palea_steindachneri: 0.446689, Pelodiscus_sinensis: 0.513994): 0.000871): 0.197470): 0.180495): 0.509581): 0.000552): 0.223238, (((Sternotherus_carinatus: 0.170780, Kinosternon_leucostomum: 0.173883): 0.881857, (Macrochelys_temminckii: 0.166527, Chelydra_serpentina: 0.399215): 0.293047): 0.178081, (((Platysternon_megacephalum: 0.473078, (Chrysemys_picta: 0.178975, (Trachemys_scripta: 0.066490, Malaclemys_terrapin: 0.070889): 0.093433): 0.266451): 0.041067, (((((Astrochelys_yniphora: 0.355808, Aldabrachelys_gigantea: 0.276384): 0.131227, Psammobates_pardalis: 0.276657): 0.104650, ((((Indotestudo_forstenii: 0.122253, Indotestudo_elongata: 0.040120): 0.152433, Testudo_horsfieldii: 0.196047): 0.053093, (Testudo_graeca: 0.264025, (Testudo_marginata: 0.140490, Testudo_kleinmanni: 0.151897): 0.055778): 0.129499): 0.046786, Malacochersus_tornieri: 0.398283): 0.066152): 0.164220, (Manouria_impressa: 0.154107, Manouria_emys: 0.293275): 0.087979): 0.004679, ((Batagur_trivittata: 0.676999, Geoemyda_spengleri: 0.333220): 0.059634, ((((Notochelys_platynota: 0.382828, (Cyclemys_fusca: 0.170889, ((Cyclemys_tcheponensis: 0.003700, Cyclemys_oldhamii: 0.004114): 0.097104, (Cyclemys_dentata: 0.082370, (Cyclemys_pulchristriata: 0.058192, Cyclemys_atripons: 0.024626): 0.163734): 0.038833): 0.092314): 0.052951): 0.028787, (Heosemys_annandalii: 0.174520, (Heosemys_grandis: 0.094093, Heosemys_depressa: 0.108666): 0.071633): 0.106937): 0.022480, (Sacalia_quadriocellata: 0.204498, Sacalia_bealei: 0.168808): 0.046533): 0.058080, (((((Cuora_flavomarginata: 0.118823, (Cuora_trifasciata: 0.063248, (Cuora_pani: 0.000005, Cuora_aurocapitata: 0.000001): 0.053452): 0.034838): 0.045162, (Cuora_mccordi: 0.085221, (Cuora_galbinifrons: 0.065013, (Cuora_picturata: 0.004031, Cuora_bourreti: 0.000005): 0.053618): 0.042542): 0.024515): 0.000005, Cuora_mouhotii: 0.136407): 0.000018, Cuora_amboinensis: 0.242727): 0.052499, ((Mauremys_leprosa: 0.159568, (((Mauremys_japonica: 0.096034, Mauremys_sinensis: 0.092545): 0.011113, ((Mauremys_reevesii: 0.043892, NC_015101.1: 0.206455): 0.069219, Mauremys_nigricans: 0.071501): 0.011749): 0.092277, (Mauremys_rivulata: 0.003952, Mauremys_caspica: 0.000005): 0.131013): 0.009455): 0.000005, (Mauremys_mutica: 0.131619, Mauremys_annamensis: 0.126772): 0.094488): 0.075406): 0.131201): 0.151654): 0.131468): 0.238027): 0.041346, (Dermochelys_coriacea: 0.431915, ((Eretmochelys_imbricata: 0.142143, (Caretta_caretta: 0.110867, (Lepidochelys_olivacea: 0.022038, Lepidochelys_kempii: 0.034226): 0.157023): 0.043870): 0.104425, (Chelonia_mydas: 0.180883, Natator_depressa: 0.212832): 0.081652): 0.047655): 0.152740): 0.068124): 0.333796): 1.181486): 1.709233);

**dN tree:**

((((((Anolis_carolinensis: 0.150615, Iguana_iguana: 0.076791): 0.053988, Anguis_fragilis: 0.165174): 0.045292, Lacerta_viridis: 0.191168): 0.069755, Smaug_warreni: 0.133532): 0.033127, (Rena_humilis: 0.214874, ((Python_regius: 0.066207, Boa_constrictor: 0.089290): 0.038939, (Protobothrops_flavoviridis: 0.118087, Dinodon_semicarinatus: 0.080953): 0.051328): 0.155502): 0.172553): 0.121006, ((Rhea_americana: 0.050059, (Gallus_gallus: 0.085262, (Columba_livia: 0.057524, (Grus_leucogeranus: 0.033729, (Falco_peregrinus: 0.096954, Pelecanus_occidentalis: 0.035441): 0.000001): 0.014972): 0.018813): 0.021316): 0.094160, ((Alligator_mississippiensis: 0.037463, Caiman_crocodylus: 0.208490): 0.013818, (Crocodylus_porosus: 0.069558, Gavialis_gangeticus: 0.039666): 0.061360): 0.551721): 0.071641, (((Podocnemis_unifilis: 0.131295, (Pelusios_castaneus: 0.039162, Pelomedusa_subrufa: 0.088116): 0.079052): 0.084724, ((Chelus_fimbriata: 0.223088, (Platemys_platycephala: 0.041080, Mesoclemmys_hogei: 0.031458): 0.038687): 0.035004, (((Elusor_macrurus: 0.030960, (Myuchelys_bellii: 0.018546, Emydura_subglobosa: 0.008441): 0.009397): 0.031573, (Chelodina_parkeri: 0.021896, (Chelodina_rugosa: 0.027709, (Chelodina_pritchardi: 0.004420, (Chelodina_mccordi: 0.004473, (Chelodina_longicollis: 0.000001, Chelodina_expansa: 0.000000): 0.001392): 0.004376): 0.009872): 0.003907): 0.055224): 0.000005, Pseudemydura_umbrina: 0.090672): 0.008362): 0.050919): 0.036913, ((Carettochelys_insculpta: 0.308619, ((Lissemys_scutata: 0.028067, Lissemys_punctata: 0.036831): 0.081675, ((Trionyx_triunguis: 0.032903, (Pelochelys_cantorii: 0.020333, Chitra_indica: 0.023991): 0.020984): 0.005108, ((Rafetus_swinhoei: 0.035626, (Apalone_spinifera: 0.010688, Apalone_ferox: 0.015539): 0.022299): 0.008531, ((Amyda_cartilaginea: 0.033876, Dogania_subplana: 0.039709): 0.001184, (Palea_steindachneri: 0.033664, Pelodiscus_sinensis: 0.038007): 0.003166): 0.018541): 0.007967): 0.025202): 0.047777): 0.024338, (((Sternotherus_carinatus: 0.017528, Kinosternon_leucostomum: 0.013120): 0.086871, (Macrochelys_temminckii: 0.038175, Chelydra_serpentina: 0.017307): 0.049971): 0.001213, (((Platysternon_megacephalum: 0.035785, (Chrysemys_picta: 0.008475, (Trachemys_scripta: 0.005716, Malaclemys_terrapin: 0.005172): 0.008184): 0.032785): 0.003299, (((((Astrochelys_yniphora: 0.019345, Aldabrachelys_gigantea: 0.012970): 0.007129, Psammobates_pardalis: 0.021774): 0.004290, ((((Indotestudo_forstenii: 0.012296, Indotestudo_elongata: 0.002594): 0.018734, Testudo_horsfieldii: 0.026410): 0.000005, (Testudo_graeca: 0.020998, (Testudo_marginata: 0.010196, Testudo_kleinmanni: 0.013950): 0.003863): 0.011857): 0.002755, Malacochersus_tornieri: 0.044141): 0.010085): 0.011601, (Manouria_impressa: 0.042995, Manouria_emys: 0.025051): 0.005274): 0.004443, ((Batagur_trivittata: 0.066806, Geoemyda_spengleri: 0.067739): 0.012355, ((((Notochelys_platynota: 0.024494, (Cyclemys_fusca: 0.043051, ((Cyclemys_tcheponensis: 0.000000, Cyclemys_oldhamii: 0.001339): 0.017169, (Cyclemys_dentata: 0.004861, (Cyclemys_pulchristriata: 0.031651, Cyclemys_atripons: 0.001201): 0.010434): 0.005383): 0.004953): 0.002521): 0.001209, (Heosemys_annandalii: 0.018464, (Heosemys_grandis: 0.007915, Heosemys_depressa: 0.010962): 0.010556): 0.006983): 0.000002, (Sacalia_quadriocellata: 0.012127, Sacalia_bealei: 0.046603): 0.011342): 0.008531, (((((Cuora_flavomarginata: 0.014863, (Cuora_trifasciata: 0.014859, (Cuora_pani: 0.000000, Cuora_aurocapitata: 0.000001): 0.006770): 0.001276): 0.001429, (Cuora_mccordi: 0.011934, (Cuora_galbinifrons: 0.003972, (Cuora_picturata: 0.001336, Cuora_bourreti: 0.000000): 0.009432): 0.008164): 0.002639): 0.000000, Cuora_mouhotii: 0.017483): 0.001187, Cuora_amboinensis: 0.009466): 0.007772, ((Mauremys_leprosa: 0.012494, (((Mauremys_japonica: 0.013721, Mauremys_sinensis: 0.010896): 0.003248, ((Mauremys_reevesii: 0.002022, NC_015101.1: 0.027331): 0.007527, Mauremys_nigricans: 0.005192): 0.002070): 0.004809, (Mauremys_rivulata: 0.001329, Mauremys_caspica: 0.000000): 0.010365): 0.002646): 0.000000, (Mauremys_mutica: 0.036176, Mauremys_annamensis: 0.012731): 0.003894): 0.001785): 0.009773): 0.007282): 0.067628): 0.008651): 0.003916, (Dermochelys_coriacea: 0.032095, ((Eretmochelys_imbricata: 0.008346, (Caretta_caretta: 0.009036, (Lepidochelys_olivacea: 0.004082, Lepidochelys_kempii: 0.002612): 0.023209): 0.003055): 0.009035, (Chelonia_mydas: 0.012597, Natator_depressa: 0.012984): 0.007607): 0.008961): 0.012763): 0.010778): 0.020843): 0.016518): 0.077147);

**w ratios as labels for TreeView:**

((((((Anolis_carolinensis #0.0545884 , Iguana_iguana #0.0222204 ) #90.133 , Anguis_fragilis #0.0145852 ) #108.194 , Lacerta_viridis #0.0486262 ) #143.692 , Smaug_warreni #0.023863 ) #126.113 , (Rena_humilis #0.0597348 , ((Python_regius #0.00697283 , Boa_constrictor #0.00140363 ) #16.9737 , (Protobothrops_flavoviridis #0.0337369 , Dinodon_semicarinatus #0.0186054 ) #0.189421 ) #85.7649 ) #1.20902 ) #193.569 , ((Rhea_americana #0.0292971 , (Gallus_gallus #0.0730797 , (Columba_livia #0.0514992 , (Grus_leucogeranus #0.0605519 , (Falco_peregrinus #0.0571684 , Pelecanus_occidentalis #0.0447121 ) #0.0001 ) #0.116428 ) #0.0341273 ) #0.0730463 ) #0.0955095 , ((Alligator_mississippiensis #0.0105796 , Caiman_crocodylus #0.0607574 ) #44.0001 , (Crocodylus_porosus #0.031012 , Gavialis_gangeticus #0.06097 ) #0.0320128 ) #237.988 ) #148.448 , (((Podocnemis_unifilis #0.0234715 , (Pelusios_castaneus #0.113244 , Pelomedusa_subrufa #0.144454 ) #0.035759 ) #40.1292 , ((Chelus_fimbriata #0.0695167 , (Platemys_platycephala #0.0916754 , Mesoclemmys_hogei #0.0455838 ) #27.3846 ) #0.0539767 , (((Elusor_macrurus #0.318808 , (Myuchelys_bellii #0.111564 , Emydura_subglobosa #0.0341395 ) #0.045493 ) #0.0574562 , (Chelodina_parkeri #0.0616023 , (Chelodina_rugosa #0.0932755 , (Chelodina_pritchardi #0.0599438 , (Chelodina_mccordi #0.128983 , (Chelodina_longicollis #0.0001 , Chelodina_expansa #0.0001 ) #0.0960075 ) #0.109715 ) #0.144686 ) #0.0279626 ) #0.0752105 ) #0.0001 , Pseudemydura_umbrina #0.0977154 ) #0.027572 ) #0.316985 ) #0.434894 , ((Carettochelys_insculpta #0.00790033 , ((Lissemys_scutata #0.121523 , Lissemys_punctata #0.0997831 ) #0.00128349 , ((Trionyx_triunguis #0.0880332 , (Pelochelys_cantorii #0.0448479 , Chitra_indica #0.038541 ) #0.287746 ) #0.0264557 , ((Rafetus_swinhoei #0.0874243 , (Apalone_spinifera #0.10049 , Apalone_ferox #0.079686 ) #0.0356628 ) #79.1692 , ((Amyda_cartilaginea #0.093218 , Dogania_subplana #0.0898326 ) #0.00685816 , (Palea_steindachneri #0.0753631 , Pelodiscus_sinensis #0.0739441 ) #3.63271 ) #0.0938927 ) #0.0441394 ) #0.0494559 ) #86.5952 ) #0.109022 , (((Sternotherus_carinatus #0.102633 , Kinosternon_leucostomum #0.0754547 ) #0.0985094 , (Macrochelys_temminckii #0.22924 , Chelydra_serpentina #0.0433527 ) #0.170522 ) #0.00681308 , (((Platysternon_megacephalum #0.0756426 , (Chrysemys_picta #0.0473554 , (Trachemys_scripta #0.0859633 , Malaclemys_terrapin #0.0729555 ) #0.0875872 ) #0.123042 ) #0.0803305 , (((((Astrochelys_yniphora #0.0543687 , Aldabrachelys_gigantea #0.0469275 ) #0.0543293 , Psammobates_pardalis #0.0787054 ) #0.0409974 , ((((Indotestudo_forstenii #0.100581 , Indotestudo_elongata #0.0646521 ) #0.122901 , Testudo_horsfieldii #0.134713 ) #0.0001 , (Testudo_graeca #0.0795311 , (Testudo_marginata #0.0725722 , Testudo_kleinmanni #0.0918406 ) #0.0692477 ) #0.0915614 ) #0.0588782 , Malacochersus_tornieri #0.110827 ) #0.152452 ) #0.0706412 , (Manouria_impressa #0.278997 , Manouria_emys #0.0854196 ) #0.0599495 ) #0.949608 , ((Batagur_trivittata #0.0986797 , Geoemyda_spengleri #0.203287 ) #0.207183 , ((((Notochelys_platynota #0.0639809 , (Cyclemys_fusca #0.251922 , ((Cyclemys_tcheponensis #0.0001 , Cyclemys_oldhamii #0.325608 ) #0.17681 , (Cyclemys_dentata #0.0590112 , (Cyclemys_pulchristriata #0.543914 , Cyclemys_atripons #0.0487526 ) #0.063724 ) #0.138624 ) #0.0536594 ) #0.0476038 ) #0.0419938 , (Heosemys_annandalii #0.105797 , (Heosemys_grandis #0.084116 , Heosemys_depressa #0.10088 ) #0.147368 ) #0.0653045 ) #0.0001 , (Sacalia_quadriocellata #0.0593024 , Sacalia_bealei #0.276073 ) #0.243741 ) #0.146881 , (((((Cuora_flavomarginata #0.125089 , (Cuora_trifasciata #0.234927 , (Cuora_pani #0.0001 , Cuora_aurocapitata #0.892586 ) #0.126654 ) #0.0366215 ) #0.0316426 , (Cuora_mccordi #0.140036 , (Cuora_galbinifrons #0.0610994 , (Cuora_picturata #0.331569 , Cuora_bourreti #0.0001 ) #0.175909 ) #0.191902 ) #0.107643 ) #0.0001 , Cuora_mouhotii #0.128171 ) #65.1908 , Cuora_amboinensis #0.0389979 ) #0.148042 , ((Mauremys_leprosa #0.0782994 , (((Mauremys_japonica #0.142873 , Mauremys_sinensis #0.117743 ) #0.292239 , ((Mauremys_reevesii #0.0460672 , NC_015101.1 #0.13238 ) #0.108743 , Mauremys_nigricans #0.072609 ) #0.176169 ) #0.0521134 , (Mauremys_rivulata #0.336409 , Mauremys_caspica #0.0001 ) #0.079113 ) #0.279814 ) #0.0001 , (Mauremys_mutica #0.274856 , Mauremys_annamensis #0.100425 ) #0.0412088 ) #0.0236753 ) #0.0744852 ) #0.0480169 ) #0.514409 ) #0.0363455 ) #0.0947165 , (Dermochelys_coriacea #0.0743097 , ((Eretmochelys_imbricata #0.0587176 , (Caretta_caretta #0.0815051 , (Lepidochelys_olivacea #0.185251 , Lepidochelys_kempii #0.0763189 ) #0.147809 ) #0.0696449 ) #0.0865221 , (Chelonia_mydas #0.0696429 , Natator_depressa #0.0610039 ) #0.093158 ) #0.188033 ) #0.0835625 ) #0.15821 ) #0.0624411 ) #0.0139804 ) #0.0451353 );

**ND3**

**dS tree:**

((((((Anolis_carolinensis: 2.338707, Iguana_iguana: 0.895605): 0.066592, Anguis_fragilis: 3.844906): 0.000044, Lacerta_viridis: 2.137629): 0.000018, Smaug_warreni: 56.157315): 0.000005, (Rena_humilis: 12.126128, ((Python_regius: 2.723582, Boa_constrictor: 9.302465): 0.000091, (Protobothrops_flavoviridis: 2.760018, Dinodon_semicarinatus: 2.871202): 0.000117): 0.000115): 0.029230): 0.000098, ((Rhea_americana: 0.977352, (Gallus_gallus: 0.492580, (Columba_livia: 0.546228, (Grus_leucogeranus: 0.846794, (Falco_peregrinus: 1.253706, Pelecanus_occidentalis: 1.361428): 0.000009): 0.000005): 0.862523): 0.281095): 0.267309, ((Alligator_mississippiensis: 1.158903, Caiman_crocodylus: 0.532512): 0.000062, (Crocodylus_porosus: 3.596063, Gavialis_gangeticus: 2.204908): 0.000107): 0.317266): 0.197971, (((Podocnemis_unifilis: 1.207062, (Pelusios_castaneus: 0.000067, Pelomedusa_subrufa: 0.850214): 3.064927): 11.348611, ((Chelus_fimbriata: 2.266577, (Platemys_platycephala: 0.292935, Mesoclemmys_hogei: 0.605959): 0.000005): 3.562707, (((Elusor_macrurus: 0.208548, (Myuchelys_bellii: 0.229278, Emydura_subglobosa: 0.245962): 0.218277): 0.808019, (Chelodina_parkeri: 0.000063, (Chelodina_rugosa: 0.418694, (Chelodina_pritchardi: 0.150418, (Chelodina_mccordi: 0.071309, (Chelodina_longicollis: 0.000000, Chelodina_expansa: 0.000000): 0.025391): 0.000005): 0.111065): 0.554820): 1.988736): 0.591748, Pseudemydura_umbrina: 0.177801): 0.000040): 0.000129): 0.000075, ((Carettochelys_insculpta: 56.020234, ((Lissemys_scutata: 0.486584, Lissemys_punctata: 0.000005): 4.448943, ((Trionyx_triunguis: 0.464536, (Pelochelys_cantorii: 0.385426, Chitra_indica: 0.214722): 0.330929): 0.000038, ((Rafetus_swinhoei: 0.540038, (Apalone_spinifera: 0.195106, Apalone_ferox: 0.163028): 0.292178): 0.000016, ((Amyda_cartilaginea: 0.394916, Dogania_subplana: 0.458577): 0.000005, (Palea_steindachneri: 0.273915, Pelodiscus_sinensis: 0.799920): 0.000057): 0.095614): 0.317281): 0.299729): 0.000183): 1.179756, (((Sternotherus_carinatus: 0.127206, Kinosternon_leucostomum: 0.387217): 1.455517, (Macrochelys_temminckii: 0.000019, Chelydra_serpentina: 0.548656): 0.406239): 0.000018, (((Platysternon_megacephalum: 0.481907, (Chrysemys_picta: 0.172291, (Trachemys_scripta: 0.168179, Malaclemys_terrapin: 0.046336): 0.073339): 0.505884): 0.214466, (((((Astrochelys_yniphora: 0.158631, Aldabrachelys_gigantea: 0.155335): 0.150193, Psammobates_pardalis: 0.382373): 0.070234, ((((Indotestudo_forstenii: 0.139746, Indotestudo_elongata: 0.075348): 0.041506, Testudo_horsfieldii: 0.110748): 0.000001, (Testudo_graeca: 0.324295, (Testudo_marginata: 0.148582, Testudo_kleinmanni: 0.109185): 0.000014): 0.254170): 0.000018, Malacochersus_tornieri: 0.283437): 0.152419): 0.058548, (Manouria_impressa: 0.432228, Manouria_emys: 0.131032): 0.197816): 0.473370, ((Batagur_trivittata: 0.461351, Geoemyda_spengleri: 0.788999): 0.074679, ((((Notochelys_platynota: 0.266604, (Cyclemys_fusca: 0.000000, ((Cyclemys_tcheponensis: 0.000000, Cyclemys_oldhamii: 0.710577): 0.000000, (Cyclemys_dentata: 0.017965, (Cyclemys_pulchristriata: 0.413730, Cyclemys_atripons: 0.000025): 0.034963): 0.731247): 0.000000): 0.520891): 0.054788, (Heosemys_annandalii: 0.472504, (Heosemys_grandis: 0.119877, Heosemys_depressa: 0.053998): 0.000042): 0.266172): 0.085830, (Sacalia_quadriocellata: 0.000041, Sacalia_bealei: 0.344832): 0.186952): 0.000005, (((((Cuora_flavomarginata: 0.170612, (Cuora_trifasciata: 0.000005, (Cuora_pani: 0.054720, Cuora_aurocapitata: 0.036335): 2.143589): 0.055413): 0.000005, (Cuora_mccordi: 0.072505, (Cuora_galbinifrons: 0.028801, (Cuora_picturata: 0.000005, Cuora_bourreti: 0.000005): 0.028867): 0.039519): 0.009506): 0.112966, Cuora_mouhotii: 0.089998): 0.019072, Cuora_amboinensis: 0.240165): 0.014328, ((Mauremys_leprosa: 0.152897, (((Mauremys_japonica: 0.044475, Mauremys_sinensis: 0.123422): 0.000005, ((Mauremys_reevesii: 0.000006, NC_015101.1: 0.027032): 0.027780, Mauremys_nigricans: 0.043615): 0.015727): 0.106279, (Mauremys_rivulata: 0.000005, Mauremys_caspica: 0.000016): 0.089468): 0.000005): 0.000006, (Mauremys_mutica: 0.082249, Mauremys_annamensis: 0.107929): 0.058678): 0.042948): 0.000009): 0.700243): 0.000067): 0.298681): 0.000024, (Dermochelys_coriacea: 1.016631, ((Eretmochelys_imbricata: 0.095093, (Caretta_caretta: 0.078333, (Lepidochelys_olivacea: 0.064473, Lepidochelys_kempii: 0.019464): 0.171681): 0.108687): 0.163402, (Chelonia_mydas: 0.125749, Natator_depressa: 0.368935): 0.000009): 0.265408): 0.000013): 0.000012): 0.000005): 1.509298): 56.267389);

**dN tree:**

((((((Anolis_carolinensis: 0.047043, Iguana_iguana: 0.062421): 0.060313, Anguis_fragilis: 0.161924): 0.037135, Lacerta_viridis: 0.143704): 0.011466, Smaug_warreni: 0.096370): 0.000000, (Rena_humilis: 0.241855, ((Python_regius: 0.082455, Boa_constrictor: 0.064488): 0.040443, (Protobothrops_flavoviridis: 0.070083, Dinodon_semicarinatus: 0.116159): 0.014532): 0.094619): 0.169593): 0.082417, ((Rhea_americana: 0.087825, (Gallus_gallus: 0.068819, (Columba_livia: 0.043033, (Grus_leucogeranus: 0.033917, (Falco_peregrinus: 0.069328, Pelecanus_occidentalis: 0.043714): 0.003390): 0.000000): 0.041495): 0.009417): 0.000027, ((Alligator_mississippiensis: 0.057815, Caiman_crocodylus: 0.076979): 0.050455, (Crocodylus_porosus: 0.042300, Gavialis_gangeticus: 0.059327): 0.044847): 0.249947): 0.009799, (((Podocnemis_unifilis: 0.064085, (Pelusios_castaneus: 0.052473, Pelomedusa_subrufa: 0.094028): 0.064839): 0.041904, ((Chelus_fimbriata: 0.067791, (Platemys_platycephala: 0.007672, Mesoclemmys_hogei: 0.019890): 0.000000): 0.035374, (((Elusor_macrurus: 0.005682, (Myuchelys_bellii: 0.000023, Emydura_subglobosa: 0.017416): 0.000022): 0.030890, (Chelodina_parkeri: 0.027828, (Chelodina_rugosa: 0.022162, (Chelodina_pritchardi: 0.010915, (Chelodina_mccordi: 0.016084, (Chelodina_longicollis: 0.000002, Chelodina_expansa: 0.000002): 0.000003): 0.000000): 0.010710): 0.021591): 0.000199): 0.046450, Pseudemydura_umbrina: 0.019460): 0.007752): 0.021536): 0.024615, ((Carettochelys_insculpta: 0.153889, ((Lissemys_scutata: 0.016351, Lissemys_punctata: 0.000000): 0.070860, ((Trionyx_triunguis: 0.022881, (Pelochelys_cantorii: 0.005405, Chitra_indica: 0.000021): 0.015752): 0.011363, ((Rafetus_swinhoei: 0.022840, (Apalone_spinifera: 0.010648, Apalone_ferox: 0.005377): 0.050536): 0.010236, ((Amyda_cartilaginea: 0.034913, Dogania_subplana: 0.208604): 0.000000, (Palea_steindachneri: 0.012832, Pelodiscus_sinensis: 0.078228): 0.015473): 0.016993): 0.006801): 0.008772): 0.069044): 0.034556, (((Sternotherus_carinatus: 0.010860, Kinosternon_leucostomum: 0.016141): 0.058975, (Macrochelys_temminckii: 0.015074, Chelydra_serpentina: 0.023254): 0.027774): 0.010050, (((Platysternon_megacephalum: 0.040986, (Chrysemys_picta: 0.040716, (Trachemys_scripta: 0.000017, Malaclemys_terrapin: 0.016860): 0.000007): 0.005603): 0.000021, (((((Astrochelys_yniphora: 0.006587, Aldabrachelys_gigantea: 0.025816): 0.007897, Psammobates_pardalis: 0.009998): 0.013519, ((((Indotestudo_forstenii: 0.033501, Indotestudo_elongata: 0.000008): 0.021962, Testudo_horsfieldii: 0.028856): 0.000002, (Testudo_graeca: 0.052386, (Testudo_marginata: 0.014792, Testudo_kleinmanni: 0.013475): 0.004881): 0.065546): 0.018226, Malacochersus_tornieri: 0.131082): 0.029761): 0.019080, (Manouria_impressa: 0.033958, Manouria_emys: 0.027359): 0.000020): 0.005589, ((Batagur_trivittata: 0.046560, Geoemyda_spengleri: 0.047131): 0.000007, ((((Notochelys_platynota: 0.041415, (Cyclemys_fusca: 0.000002, ((Cyclemys_tcheponensis: 0.000002, Cyclemys_oldhamii: 0.030999): 0.000002, (Cyclemys_dentata: 0.018073, (Cyclemys_pulchristriata: 0.048281, Cyclemys_atripons: 0.024826): 0.000003): 0.000073): 0.000002): 0.005750): 0.000005, (Heosemys_annandalii: 0.010903, (Heosemys_grandis: 0.011377, Heosemys_depressa: 0.005668): 0.011374): 0.011280): 0.000009, (Sacalia_quadriocellata: 0.041005, Sacalia_bealei: 0.017683): 0.000019): 0.000000, (((((Cuora_flavomarginata: 0.005544, (Cuora_trifasciata: 0.000000, (Cuora_pani: 0.007981, Cuora_aurocapitata: 0.170524): 1.360096): 0.005677): 0.000000, (Cuora_mccordi: 0.040692, (Cuora_galbinifrons: 0.000003, (Cuora_picturata: 0.000000, Cuora_bourreti: 0.000000): 0.005489): 0.000004): 0.000001): 0.000011, Cuora_mouhotii: 0.023347): 0.000002, Cuora_amboinensis: 0.022541): 0.000001, ((Mauremys_leprosa: 0.011071, (((Mauremys_japonica: 0.005515, Mauremys_sinensis: 0.028769): 0.000000, ((Mauremys_reevesii: 0.005521, NC_015101.1: 0.000003): 0.000003, Mauremys_nigricans: 0.005551): 0.000002): 0.000011, (Mauremys_rivulata: 0.000000, Mauremys_caspica: 0.016471): 0.010999): 0.000000): 0.005506, (Mauremys_mutica: 0.000008, Mauremys_annamensis: 0.005372): 0.000006): 0.000004): 0.005629): 0.000070): 0.066731): 0.011231): 0.011009, (Dermochelys_coriacea: 0.039284, ((Eretmochelys_imbricata: 0.005185, (Caretta_caretta: 0.017094, (Lepidochelys_olivacea: 0.000006, Lepidochelys_kempii: 0.000002): 0.010800): 0.016307): 0.010444, (Chelonia_mydas: 0.010455, Natator_depressa: 0.015827): 0.005071): 0.000027): 0.005606): 0.005522): 0.000000): 0.021098): 0.050183);

**w ratios as labels for TreeView:**

((((((Anolis_carolinensis #0.0201151 , Iguana_iguana #0.0696967 ) #0.905716 , Anguis_fragilis #0.0421138 ) #834.553 , Lacerta_viridis #0.0672257 ) #623.723 , Smaug_warreni #0.00171608 ) #0.0001 , (Rena_humilis #0.0199449 , ((Python_regius #0.0302746 , Boa_constrictor #0.00693239 ) #445.103 , (Protobothrops_flavoviridis #0.0253922 , Dinodon_semicarinatus #0.0404566 ) #123.686 ) #821.696 ) #5.8021 ) #843.159 , ((Rhea_americana #0.0898603 , (Gallus_gallus #0.139711 , (Columba_livia #0.0787817 , (Grus_leucogeranus #0.0400529 , (Falco_peregrinus #0.0552985 , Pelecanus_occidentalis #0.0321091 ) #363.393 ) #0.0001 ) #0.0481091 ) #0.0335028 ) #0.0001 , ((Alligator_mississippiensis #0.0498875 , Caiman_crocodylus #0.144558 ) #819.12 , (Crocodylus_porosus #0.0117627 , Gavialis_gangeticus #0.0269068 ) #417.664 ) #0.787813 ) #0.0494964 , (((Podocnemis_unifilis #0.0530917 , (Pelusios_castaneus #777.791 , Pelomedusa_subrufa #0.110593 ) #0.021155 ) #0.00369244 , ((Chelus_fimbriata #0.0299088 , (Platemys_platycephala #0.0261889 , Mesoclemmys_hogei #0.0328244 ) #0.0001 ) #0.00992905 , (((Elusor_macrurus #0.027247 , (Myuchelys_bellii #0.0001 , Emydura_subglobosa #0.0708084 ) #0.0001 ) #0.0382294 , (Chelodina_parkeri #441.335 , (Chelodina_rugosa #0.0529313 , (Chelodina_pritchardi #0.0725619 , (Chelodina_mccordi #0.225554 , (Chelodina_longicollis #4.7194 , Chelodina_expansa #4.83586 ) #0.0001 ) #0.0001 ) #0.0964329 ) #0.0389148 ) #0.0001 ) #0.0784961 , Pseudemydura_umbrina #0.109447 ) #195.648 ) #166.807 ) #327.389 , ((Carettochelys_insculpta #0.00274703 , ((Lissemys_scutata #0.033603 , Lissemys_punctata #0.0001 ) #0.0159274 , ((Trionyx_triunguis #0.0492563 , (Pelochelys_cantorii #0.0140231 , Chitra_indica #0.0001 ) #0.0476001 ) #300.498 , ((Rafetus_swinhoei #0.0422932 , (Apalone_spinifera #0.0545745 , Apalone_ferox #0.0329803 ) #0.172964 ) #647.043 , ((Amyda_cartilaginea #0.0884057 , Dogania_subplana #0.454894 ) #0.0001 , (Palea_steindachneri #0.0468454 , Pelodiscus_sinensis #0.0977942 ) #272.509 ) #0.177728 ) #0.021435 ) #0.0292673 ) #376.401 ) #0.0292905 , (((Sternotherus_carinatus #0.0853742 , Kinosternon_leucostomum #0.0416856 ) #0.0405185 , (Macrochelys_temminckii #798.425 , Chelydra_serpentina #0.0423842 ) #0.0683682 ) #550.822 , (((Platysternon_megacephalum #0.0850502 , (Chrysemys_picta #0.236324 , (Trachemys_scripta #0.0001 , Malaclemys_terrapin #0.363862 ) #0.0001 ) #0.0110749 ) #0.0001 , (((((Astrochelys_yniphora #0.0415266 , Aldabrachelys_gigantea #0.166198 ) #0.0525772 , Psammobates_pardalis #0.0261475 ) #0.192483 , ((((Indotestudo_forstenii #0.239731 , Indotestudo_elongata #0.0001 ) #0.529115 , Testudo_horsfieldii #0.260552 ) #2.6358 , (Testudo_graeca #0.161539 , (Testudo_marginata #0.0995551 , Testudo_kleinmanni #0.123416 ) #338.68 ) #0.257882 ) #999 , Malacochersus_tornieri #0.462474 ) #0.195257 ) #0.325889 , (Manouria_impressa #0.0785658 , Manouria_emys #0.208796 ) #0.0001 ) #0.0118058 , ((Batagur_trivittata #0.100922 , Geoemyda_spengleri #0.059735 ) #0.000100088 , ((((Notochelys_platynota #0.155342 , (Cyclemys_fusca #32.2555 , ((Cyclemys_tcheponensis #24.8658 , Cyclemys_oldhamii #0.0436246 ) #25.3241 , (Cyclemys_dentata #1.00604 , (Cyclemys_pulchristriata #0.116696 , Cyclemys_atripons #999 ) #0.0001 ) #0.0001 ) #23.2328 ) #0.0110392 ) #0.0001 , (Heosemys_annandalii #0.0230748 , (Heosemys_grandis #0.094902 , Heosemys_depressa #0.104969 ) #272.627 ) #0.0423785 ) #0.0001 , (Sacalia_quadriocellata #999 , Sacalia_bealei #0.0512814 ) #0.0001 ) #0.0001 , (((((Cuora_flavomarginata #0.0324939 , (Cuora_trifasciata #0.0001 , (Cuora_pani #0.14585 , Cuora_aurocapitata #4.6931 ) #0.634495 ) #0.102444 ) #0.0001 , (Cuora_mccordi #0.561229 , (Cuora_galbinifrons #0.0001 , (Cuora_picturata #0.0001 , Cuora_bourreti #0.0001 ) #0.190138 ) #0.0001 ) #0.0001 ) #0.0001 , Cuora_mouhotii #0.259421 ) #0.0001 , Cuora_amboinensis #0.0938568 ) #0.0001 , ((Mauremys_leprosa #0.0724061 , (((Mauremys_japonica #0.123996 , Mauremys_sinensis #0.233096 ) #0.0001 , ((Mauremys_reevesii #999 , NC_015101.1 #0.0001 ) #0.0001 , Mauremys_nigricans #0.127262 ) #0.0001 ) #0.0001 , (Mauremys_rivulata #0.0001 , Mauremys_caspica #999 ) #0.122938 ) #0.0001 ) #900.385 , (Mauremys_mutica #0.0001 , Mauremys_annamensis #0.0497746 ) #0.0001 ) #0.0001 ) #622.03 ) #0.0001 ) #999 ) #0.0376003 ) #450.15 , (Dermochelys_coriacea #0.0386414 , ((Eretmochelys_imbricata #0.0545227 , (Caretta_caretta #0.218215 , (Lepidochelys_olivacea #0.0001 , Lepidochelys_kempii #0.0001 ) #0.0629056 ) #0.150034 ) #0.0639165 , (Chelonia_mydas #0.0831384 , Natator_depressa #0.0429004 ) #541.794 ) #0.0001 ) #416.09 ) #459.741 ) #0.0001 ) #0.0139785 ) #0.000891872 );

**ND4**

**dS tree:**

((((((Anolis_carolinensis: 2.095255, Iguana_iguana: 4.672785): 0.026159, Anguis_fragilis: 2.110016): 0.000029, Lacerta_viridis: 3.492156): 0.000016, Smaug_warreni: 2.721944): 0.000015, (Rena_humilis: 5.419001, ((Python_regius: 4.072033, Boa_constrictor: 15.550769): 0.000061, (Protobothrops_flavoviridis: 2.348768, Dinodon_semicarinatus: 3.650019): 0.000032): 0.000075): 0.457028): 0.000105, ((Rhea_americana: 0.950732, (Gallus_gallus: 1.483697, (Columba_livia: 1.009219, (Grus_leucogeranus: 0.851093, (Falco_peregrinus: 1.322903, Pelecanus_occidentalis: 0.817690): 0.000011): 0.170599): 0.182319): 0.245969): 0.779909, ((Alligator_mississippiensis: 0.796323, Caiman_crocodylus: 1.862429): 0.000069, (Crocodylus_porosus: 1.720389, Gavialis_gangeticus: 1.522074): 1.475815): 1.201484): 0.000052, (((Podocnemis_unifilis: 2.217577, (Pelusios_castaneus: 0.209718, Pelomedusa_subrufa: 0.925064): 1.891784): 1.413490, ((Chelus_fimbriata: 0.868862, (Platemys_platycephala: 0.510334, Mesoclemmys_hogei: 0.469807): 0.345693): 0.632323, (((Elusor_macrurus: 0.248480, (Myuchelys_bellii: 0.083487, Emydura_subglobosa: 0.281472): 0.295962): 0.614578, (Chelodina_parkeri: 0.364801, (Chelodina_rugosa: 0.328566, (Chelodina_pritchardi: 0.057286, (Chelodina_mccordi: 0.039243, (Chelodina_longicollis: 0.000005, Chelodina_expansa: 0.007884): 0.041610): 0.036355): 0.144603): 0.326915): 0.369577): 0.231866, Pseudemydura_umbrina: 0.831932): 0.096121): 0.175329): 0.000070, ((Carettochelys_insculpta: 6.244146, ((Lissemys_scutata: 0.528854, Lissemys_punctata: 0.344796): 2.037746, ((Trionyx_triunguis: 0.566909, (Pelochelys_cantorii: 0.293560, Chitra_indica: 0.342094): 0.048106): 0.315953, ((Rafetus_swinhoei: 0.336680, (Apalone_spinifera: 0.199117, Apalone_ferox: 0.102933): 0.335187): 0.087050, ((Amyda_cartilaginea: 0.481708, Dogania_subplana: 0.568916): 0.126005, (Palea_steindachneri: 0.516369, Pelodiscus_sinensis: 0.396296): 0.008917): 0.081436): 0.000003): 0.445926): 0.000047): 0.143625, (((Sternotherus_carinatus: 0.372399, Kinosternon_leucostomum: 0.152523): 1.002573, (Macrochelys_temminckii: 0.273319, Chelydra_serpentina: 0.422901): 0.286581): 0.075344, (((Platysternon_megacephalum: 0.884615, (Chrysemys_picta: 0.194290, (Trachemys_scripta: 0.108986, Malaclemys_terrapin: 0.054425): 0.087741): 0.415959): 0.117428, (((((Astrochelys_yniphora: 0.190175, Aldabrachelys_gigantea: 0.394023): 0.204377, Psammobates_pardalis: 0.453183): 0.034995, ((((Indotestudo_forstenii: 0.093597, Indotestudo_elongata: 0.079504): 0.128021, Testudo_horsfieldii: 0.234092): 0.022622, (Testudo_graeca: 0.194961, (Testudo_marginata: 0.101960, Testudo_kleinmanni: 0.137952): 0.058339): 0.129984): 0.009070, Malacochersus_tornieri: 0.298162): 0.244356): 0.247255, (Manouria_impressa: 0.258095, Manouria_emys: 0.263344): 0.250526): 0.070536, ((Batagur_trivittata: 0.646613, Geoemyda_spengleri: 0.465186): 0.137545, ((((Notochelys_platynota: 0.394424, (Cyclemys_fusca: 0.248271, ((Cyclemys_tcheponensis: 0.083724, Cyclemys_oldhamii: 0.048359): 0.128937, (Cyclemys_dentata: 0.086593, (Cyclemys_pulchristriata: 0.094993, Cyclemys_atripons: 0.029060): 0.036790): 0.098343): 0.063696): 0.043542): 0.053936, (Heosemys_annandalii: 0.232504, (Heosemys_grandis: 0.185376, Heosemys_depressa: 0.165392): 0.080544): 0.210074): 0.005956, (Sacalia_quadriocellata: 0.167858, Sacalia_bealei: 0.166696): 0.114450): 0.177119, (((((Cuora_flavomarginata: 0.136078, (Cuora_trifasciata: 0.046771, (Cuora_pani: 0.000000, Cuora_aurocapitata: 0.000000): 0.066114): 0.038210): 0.060690, (Cuora_mccordi: 0.076324, (Cuora_galbinifrons: 0.039029, (Cuora_picturata: 0.000000, Cuora_bourreti: 0.000000): 0.089715): 0.058827): 0.019441): 0.000004, Cuora_mouhotii: 0.222824): 0.000002, Cuora_amboinensis: 0.208775): 0.029768, ((Mauremys_leprosa: 0.174512, (((Mauremys_japonica: 0.135371, Mauremys_sinensis: 0.067404): 0.010533, ((Mauremys_reevesii: 0.000005, NC_015101.1: 0.011046): 0.063248, Mauremys_nigricans: 0.072523): 0.014093): 0.067499, (Mauremys_rivulata: 0.000005, Mauremys_caspica: 0.002752): 0.143847): 0.028545): 0.000003, (Mauremys_mutica: 0.099408, Mauremys_annamensis: 0.108502): 0.085562): 0.077627): 0.133893): 0.000013): 0.094328): 0.346161): 0.069302, (Dermochelys_coriacea: 0.782100, ((Eretmochelys_imbricata: 0.205372, (Caretta_caretta: 0.159345, (Lepidochelys_olivacea: 0.010953, Lepidochelys_kempii: 0.020430): 0.102081): 0.082268): 0.156088, (Chelonia_mydas: 0.196656, Natator_depressa: 0.206779): 0.009884): 0.113074): 0.199407): 0.082897): 0.714238): 0.551743): 2.574309);

**dN tree:**

((((((Anolis_carolinensis: 0.133466, Iguana_iguana: 0.063930): 0.036865, Anguis_fragilis: 0.143339): 0.029274, Lacerta_viridis: 0.172661): 0.016252, Smaug_warreni: 0.152859): 0.015461, (Rena_humilis: 0.155440, ((Python_regius: 0.065533, Boa_constrictor: 0.053973): 0.023271, (Protobothrops_flavoviridis: 0.060767, Dinodon_semicarinatus: 0.065518): 0.029268): 0.075194): 0.127195): 0.069583, ((Rhea_americana: 0.038306, (Gallus_gallus: 0.054327, (Columba_livia: 0.039506, (Grus_leucogeranus: 0.018719, (Falco_peregrinus: 0.087969, Pelecanus_occidentalis: 0.014042): 0.002507): 0.005956): 0.001915): 0.021222): 0.114205, ((Alligator_mississippiensis: 0.075454, Caiman_crocodylus: 0.180565): 0.042180, (Crocodylus_porosus: 0.047815, Gavialis_gangeticus: 0.037622): 0.083050): 0.191706): 0.026078, (((Podocnemis_unifilis: 0.074647, (Pelusios_castaneus: 0.020502, Pelomedusa_subrufa: 0.021376): 0.064144): 0.043968, ((Chelus_fimbriata: 0.066448, (Platemys_platycephala: 0.037408, Mesoclemmys_hogei: 0.023045): 0.014171): 0.017351, (((Elusor_macrurus: 0.014004, (Myuchelys_bellii: 0.010129, Emydura_subglobosa: 0.013184): 0.011135): 0.023273, (Chelodina_parkeri: 0.009410, (Chelodina_rugosa: 0.015021, (Chelodina_pritchardi: 0.004731, (Chelodina_mccordi: 0.002034, (Chelodina_longicollis: 0.000000, Chelodina_expansa: 0.000001): 0.002271): 0.005175): 0.011951): 0.007960): 0.048416): 0.015305, Pseudemydura_umbrina: 0.049602): 0.017103): 0.043043): 0.017994, ((Carettochelys_insculpta: 0.117092, ((Lissemys_scutata: 0.031505, Lissemys_punctata: 0.023584): 0.058522, ((Trionyx_triunguis: 0.030184, (Pelochelys_cantorii: 0.024438, Chitra_indica: 0.010758): 0.000005): 0.004265, ((Rafetus_swinhoei: 0.015503, (Apalone_spinifera: 0.010905, Apalone_ferox: 0.010424): 0.011498): 0.000976, ((Amyda_cartilaginea: 0.016270, Dogania_subplana: 0.032243): 0.006152, (Palea_steindachneri: 0.018353, Pelodiscus_sinensis: 0.014216): 0.001798): 0.003152): 0.003106): 0.017060): 0.046514): 0.024756, (((Sternotherus_carinatus: 0.003665, Kinosternon_leucostomum: 0.017529): 0.087053, (Macrochelys_temminckii: 0.013472, Chelydra_serpentina: 0.018272): 0.020181): 0.008947, (((Platysternon_megacephalum: 0.042704, (Chrysemys_picta: 0.018927, (Trachemys_scripta: 0.006292, Malaclemys_terrapin: 0.009916): 0.008506): 0.040849): 0.004820, (((((Astrochelys_yniphora: 0.020515, Aldabrachelys_gigantea: 0.015487): 0.001120, Psammobates_pardalis: 0.020021): 0.008984, ((((Indotestudo_forstenii: 0.009427, Indotestudo_elongata: 0.004509): 0.017490, Testudo_horsfieldii: 0.016926): 0.001597, (Testudo_graeca: 0.015973, (Testudo_marginata: 0.003983, Testudo_kleinmanni: 0.014499): 0.004832): 0.007463): 0.002663, Malacochersus_tornieri: 0.030256): 0.018919): 0.003842, (Manouria_impressa: 0.016706, Manouria_emys: 0.012847): 0.007483): 0.005345, ((Batagur_trivittata: 0.044075, Geoemyda_spengleri: 0.054495): 0.011814, ((((Notochelys_platynota: 0.032056, (Cyclemys_fusca: 0.013428, ((Cyclemys_tcheponensis: 0.038539, Cyclemys_oldhamii: 0.015411): 0.002956, (Cyclemys_dentata: 0.010029, (Cyclemys_pulchristriata: 0.016211, Cyclemys_atripons: 0.009323): 0.002005): 0.008868): 0.005157): 0.003448): 0.001339, (Heosemys_annandalii: 0.025099, (Heosemys_grandis: 0.011981, Heosemys_depressa: 0.013183): 0.002700): 0.007629): 0.005319, (Sacalia_quadriocellata: 0.014126, Sacalia_bealei: 0.029814): 0.001886): 0.005850, (((((Cuora_flavomarginata: 0.010769, (Cuora_trifasciata: 0.002320, (Cuora_pani: 0.000002, Cuora_aurocapitata: 0.000002): 0.006424): 0.001042): 0.003058, (Cuora_mccordi: 0.004896, (Cuora_galbinifrons: 0.004290, (Cuora_picturata: 0.000002, Cuora_bourreti: 0.000002): 0.003243): 0.003663): 0.004124): 0.002646, Cuora_mouhotii: 0.010919): 0.001080, Cuora_amboinensis: 0.014089): 0.002064, ((Mauremys_leprosa: 0.021465, (((Mauremys_japonica: 0.007831, Mauremys_sinensis: 0.007692): 0.000001, ((Mauremys_reevesii: 0.000000, NC_015101.1: 0.000001): 0.005514, Mauremys_nigricans: 0.004329): 0.002278): 0.001848, (Mauremys_rivulata: 0.000000, Mauremys_caspica: 0.000000): 0.002380): 0.000003): 0.002311, (Mauremys_mutica: 0.008824, Mauremys_annamensis: 0.010856): 0.001009): 0.002169): 0.007481): 0.008070): 0.049758): 0.013577): 0.005669, (Dermochelys_coriacea: 0.022568, ((Eretmochelys_imbricata: 0.021548, (Caretta_caretta: 0.003256, (Lepidochelys_olivacea: 0.003027, Lepidochelys_kempii: 0.001142): 0.011779): 0.008208): 0.004615, (Chelonia_mydas: 0.006425, Natator_depressa: 0.013823): 0.000001): 0.010001): 0.007173): 0.006497): 0.015465): 0.017063): 0.054083);

**w ratios as labels for TreeView:**

((((((Anolis_carolinensis #0.0636991 , Iguana_iguana #0.0136813 ) #1.40926 , Anguis_fragilis #0.0679328 ) #999 , Lacerta_viridis #0.0494425 ) #999 , Smaug_warreni #0.0561581 ) #999 , (Rena_humilis #0.0286843 , ((Python_regius #0.0160934 , Boa_constrictor #0.00347075 ) #382.346 , (Protobothrops_flavoviridis #0.0258717 , Dinodon_semicarinatus #0.0179499 ) #906.267 ) #999 ) #0.278309 ) #665.669 , ((Rhea_americana #0.0402908 , (Gallus_gallus #0.036616 , (Columba_livia #0.0391448 , (Grus_leucogeranus #0.021994 , (Falco_peregrinus #0.066497 , Pelecanus_occidentalis #0.0171731 ) #223.697 ) #0.034914 ) #0.010503 ) #0.0862802 ) #0.146433 , ((Alligator_mississippiensis #0.0947528 , Caiman_crocodylus #0.0969513 ) #611.345 , (Crocodylus_porosus #0.0277934 , Gavialis_gangeticus #0.0247174 ) #0.0562738 ) #0.159558 ) #498.256 , (((Podocnemis_unifilis #0.0336613 , (Pelusios_castaneus #0.0977599 , Pelomedusa_subrufa #0.023108 ) #0.0339069 ) #0.0311059 , ((Chelus_fimbriata #0.0764765 , (Platemys_platycephala #0.0733002 , Mesoclemmys_hogei #0.0490531 ) #0.0409942 ) #0.0274406 , (((Elusor_macrurus #0.0563585 , (Myuchelys_bellii #0.121326 , Emydura_subglobosa #0.0468399 ) #0.0376228 ) #0.0378679 , (Chelodina_parkeri #0.0257951 , (Chelodina_rugosa #0.0457178 , (Chelodina_pritchardi #0.0825777 , (Chelodina_mccordi #0.0518414 , (Chelodina_longicollis #0.0001 , Chelodina_expansa #0.0001 ) #0.0545799 ) #0.142344 ) #0.0826449 ) #0.0243499 ) #0.131003 ) #0.0660101 , Pseudemydura_umbrina #0.059623 ) #0.177934 ) #0.245497 ) #257.922 , ((Carettochelys_insculpta #0.0187522 , ((Lissemys_scutata #0.0595728 , Lissemys_punctata #0.0683995 ) #0.0287189 , ((Trionyx_triunguis #0.0532433 , (Pelochelys_cantorii #0.0832486 , Chitra_indica #0.0314477 ) #0.0001 ) #0.0134998 , ((Rafetus_swinhoei #0.0460481 , (Apalone_spinifera #0.0547679 , Apalone_ferox #0.101266 ) #0.0343043 ) #0.0112075 , ((Amyda_cartilaginea #0.0337754 , Dogania_subplana #0.0566744 ) #0.0488204 , (Palea_steindachneri #0.0355415 , Pelodiscus_sinensis #0.0358729 ) #0.20163 ) #0.0387088 ) #999 ) #0.0382584 ) #999 ) #0.172363 , (((Sternotherus_carinatus #0.00984118 , Kinosternon_leucostomum #0.114929 ) #0.0868292 , (Macrochelys_temminckii #0.0492897 , Chelydra_serpentina #0.0432063 ) #0.0704201 ) #0.118751 , (((Platysternon_megacephalum #0.0482739 , (Chrysemys_picta #0.0974145 , (Trachemys_scripta #0.0577353 , Malaclemys_terrapin #0.182199 ) #0.0969422 ) #0.0982036 ) #0.0410425 , (((((Astrochelys_yniphora #0.107875 , Aldabrachelys_gigantea #0.0393057 ) #0.00547879 , Psammobates_pardalis #0.0441795 ) #0.256714 , ((((Indotestudo_forstenii #0.100719 , Indotestudo_elongata #0.0567197 ) #0.136615 , Testudo_horsfieldii #0.0723063 ) #0.0706082 , (Testudo_graeca #0.0819303 , (Testudo_marginata #0.0390604 , Testudo_kleinmanni #0.1051 ) #0.082829 ) #0.0574151 ) #0.293638 , Malacochersus_tornieri #0.101475 ) #0.0774228 ) #0.0155367 , (Manouria_impressa #0.0647263 , Manouria_emys #0.0487823 ) #0.0298685 ) #0.0757843 , ((Batagur_trivittata #0.0681626 , Geoemyda_spengleri #0.117147 ) #0.085892 , ((((Notochelys_platynota #0.0812731 , (Cyclemys_fusca #0.0540851 , ((Cyclemys_tcheponensis #0.460306 , Cyclemys_oldhamii #0.318679 ) #0.0229268 , (Cyclemys_dentata #0.115821 , (Cyclemys_pulchristriata #0.170656 , Cyclemys_atripons #0.320807 ) #0.0544975 ) #0.0901715 ) #0.0809594 ) #0.0791792 ) #0.0248259 , (Heosemys_annandalii #0.107952 , (Heosemys_grandis #0.0646297 , Heosemys_depressa #0.079709 ) #0.0335227 ) #0.0363162 ) #0.893103 , (Sacalia_quadriocellata #0.084154 , Sacalia_bealei #0.178849 ) #0.016479 ) #0.0330309 , (((((Cuora_flavomarginata #0.0791401 , (Cuora_trifasciata #0.0496037 , (Cuora_pani #35.1696 , Cuora_aurocapitata #38.4647 ) #0.0971603 ) #0.0272712 ) #0.0503822 , (Cuora_mccordi #0.0641531 , (Cuora_galbinifrons #0.109919 , (Cuora_picturata #38.3359 , Cuora_bourreti #44.1287 ) #0.0361516 ) #0.0622589 ) #0.212135 ) #642.86 , Cuora_mouhotii #0.0490024 ) #472.138 , Cuora_amboinensis #0.0674847 ) #0.0693394 , ((Mauremys_leprosa #0.122998 , (((Mauremys_japonica #0.0578453 , Mauremys_sinensis #0.114113 ) #0.0001 , ((Mauremys_reevesii #0.0001 , NC_015101.1 #0.0001 ) #0.0871778 , Mauremys_nigricans #0.0596955 ) #0.16162 ) #0.0273839 , (Mauremys_rivulata #0.0001 , Mauremys_caspica #0.0001 ) #0.0165436 ) #0.0001 ) #716.641 , (Mauremys_mutica #0.088769 , Mauremys_annamensis #0.100049 ) #0.0117883 ) #0.0279427 ) #0.0558732 ) #616.402 ) #0.527497 ) #0.0392218 ) #0.0818022 , (Dermochelys_coriacea #0.0288562 , ((Eretmochelys_imbricata #0.10492 , (Caretta_caretta #0.0204352 , (Lepidochelys_olivacea #0.276342 , Lepidochelys_kempii #0.0559176 ) #0.115391 ) #0.099773 ) #0.0295682 , (Chelonia_mydas #0.0326711 , Natator_depressa #0.0668505 ) #0.0001 ) #0.0884496 ) #0.0359734 ) #0.0783711 ) #0.0216519 ) #0.0309261 ) #0.0210089 );

**ND4L**

**dS tree:**

((((((Anolis_carolinensis: 0.199036, Iguana_iguana: 11.983707): 47.093582, Anguis_fragilis: 0.881842): 0.000004, Lacerta_viridis: 0.507106): 0.000004, Smaug_warreni: 1.400777): 0.030597, (Rena_humilis: 46.605843, ((Python_regius: 0.000073, Boa_constrictor: 3.198344): 6.069736, (Protobothrops_flavoviridis: 0.695393, Dinodon_semicarinatus: 2.647506): 0.000004): 0.000348): 0.000237): 46.643975, ((Rhea_americana: 12.118898, (Gallus_gallus: 47.078262, (Columba_livia: 0.803696, (Grus_leucogeranus: 1.859697, (Falco_peregrinus: 46.885759, Pelecanus_occidentalis: 0.000071): 0.000004): 0.000004): 0.000004): 1.582345): 0.000115, ((Alligator_mississippiensis: 12.050058, Caiman_crocodylus: 11.667547): 0.000095, (Crocodylus_porosus: 1.975303, Gavialis_gangeticus: 0.274629): 0.000123): 0.000495): 0.000004, (((Podocnemis_unifilis: 2.859525, (Pelusios_castaneus: 0.364676, Pelomedusa_subrufa: 0.175002): 0.000204): 0.953564, ((Chelus_fimbriata: 0.790580, (Platemys_platycephala: 1.176758, Mesoclemmys_hogei: 0.000004): 0.622891): 1.117765, (((Elusor_macrurus: 0.000004, (Myuchelys_bellii: 0.401461, Emydura_subglobosa: 0.449696): 0.000017): 0.000020, (Chelodina_parkeri: 0.000021, (Chelodina_rugosa: 0.246632, (Chelodina_pritchardi: 0.000004, (Chelodina_mccordi: 0.062440, (Chelodina_longicollis: 0.000004, Chelodina_expansa: 0.000004): 0.008680): 0.087133): 0.132860): 0.665775): 47.101374): 0.000004, Pseudemydura_umbrina: 2.048839): 0.000004): 47.111362): 0.000103, ((Carettochelys_insculpta: 2.611873, ((Lissemys_scutata: 0.163359, Lissemys_punctata: 0.115997): 0.605617, ((Trionyx_triunguis: 0.492014, (Pelochelys_cantorii: 0.254361, Chitra_indica: 0.282617): 0.407563): 0.000018, ((Rafetus_swinhoei: 0.515566, (Apalone_spinifera: 0.000016, Apalone_ferox: 46.455643): 0.256970): 0.000018, ((Amyda_cartilaginea: 0.188338, Dogania_subplana: 0.282114): 0.144424, (Palea_steindachneri: 0.341036, Pelodiscus_sinensis: 0.188776): 0.722794): 0.058966): 0.000004): 0.000012): 0.229982): 0.000010, (((Sternotherus_carinatus: 0.000021, Kinosternon_leucostomum: 0.381837): 2.368103, (Macrochelys_temminckii: 0.000047, Chelydra_serpentina: 0.614945): 0.390174): 0.076993, (((Platysternon_megacephalum: 0.332121, (Chrysemys_picta: 0.254491, (Trachemys_scripta: 0.000011, Malaclemys_terrapin: 0.135592): 0.012170): 1.263620): 0.000004, (((((Astrochelys_yniphora: 0.275800, Aldabrachelys_gigantea: 0.033060): 0.000004, Psammobates_pardalis: 0.536309): 0.000011, ((((Indotestudo_forstenii: 0.143868, Indotestudo_elongata: 0.020729): 0.225958, Testudo_horsfieldii: 0.204954): 0.086899, (Testudo_graeca: 0.269496, (Testudo_marginata: 0.027512, Testudo_kleinmanni: 0.298418): 0.000004): 0.000004): 0.000004, Malacochersus_tornieri: 0.661870): 0.165313): 0.000021, (Manouria_impressa: 0.000014, Manouria_emys: 0.319605): 0.479935): 0.000025, ((Batagur_trivittata: 0.399055, Geoemyda_spengleri: 0.021546): 0.058966, ((((Notochelys_platynota: 0.229674, (Cyclemys_fusca: 0.000000, ((Cyclemys_tcheponensis: 0.000000, Cyclemys_oldhamii: 0.616077): 0.000000, (Cyclemys_dentata: 0.000010, (Cyclemys_pulchristriata: 0.566841, Cyclemys_atripons: 0.026245): 0.129459): 0.265206): 0.000000): 0.521800): 0.000004, (Heosemys_annandalii: 0.273366, (Heosemys_grandis: 0.043167, Heosemys_depressa: 0.112179): 0.056628): 0.160654): 0.096988, (Sacalia_quadriocellata: 0.096021, Sacalia_bealei: 0.358776): 0.050730): 0.000006, (((((Cuora_flavomarginata: 0.178368, (Cuora_trifasciata: 0.067897, (Cuora_pani: 0.000000, Cuora_aurocapitata: 0.000000): 0.063874): 0.046246): 0.000004, (Cuora_mccordi: 0.000004, (Cuora_galbinifrons: 0.042275, (Cuora_picturata: 0.000004, Cuora_bourreti: 0.000004): 0.042104): 0.000011): 0.090484): 0.000004, Cuora_mouhotii: 0.239651): 0.000004, Cuora_amboinensis: 0.020962): 0.029279, ((Mauremys_leprosa: 0.146514, (((Mauremys_japonica: 0.028200, Mauremys_sinensis: 0.074409): 0.000004, ((Mauremys_reevesii: 0.000004, NC_015101.1: 0.000004): 0.066644, Mauremys_nigricans: 0.050411): 0.027582): 0.118268, (Mauremys_rivulata: 0.000000, Mauremys_caspica: 0.000000): 0.101546): 0.022476): 0.000004, (Mauremys_mutica: 0.244973, Mauremys_annamensis: 0.171826): 0.027916): 0.028068): 0.180189): 0.078532): 0.314645): 0.267795): 0.000004, (Dermochelys_coriacea: 0.550224, ((Eretmochelys_imbricata: 0.012508, (Caretta_caretta: 0.329295, (Lepidochelys_olivacea: 0.019099, Lepidochelys_kempii: 0.000004): 0.000010): 0.127087): 0.197271, (Chelonia_mydas: 0.000004, Natator_depressa: 0.274094): 0.000004): 0.226855): 0.037396): 0.259761): 0.000004): 13.272545): 0.000036);

**dN tree:**

((((((Anolis_carolinensis: 0.162450, Iguana_iguana: 0.211174): 0.048567, Anguis_fragilis: 0.172140): 0.000000, Lacerta_viridis: 0.671122): 0.000000, Smaug_warreni: 0.276331): 0.000003, (Rena_humilis: 0.314952, ((Python_regius: 0.044522, Boa_constrictor: 0.155519): 0.062450, (Protobothrops_flavoviridis: 0.129080, Dinodon_semicarinatus: 0.208729): 0.000000): 0.347299): 0.237093): 0.294126, ((Rhea_americana: 0.082886, (Gallus_gallus: 0.056934, (Columba_livia: 0.034279, (Grus_leucogeranus: 0.000186, (Falco_peregrinus: 0.162072, Pelecanus_occidentalis: 0.070507): 0.000000): 0.000000): 0.000000): 0.025391): 0.115088, ((Alligator_mississippiensis: 0.099492, Caiman_crocodylus: 0.190510): 0.073500, (Crocodylus_porosus: 0.118106, Gavialis_gangeticus: 0.024093): 0.122590): 0.494661): 0.000000, (((Podocnemis_unifilis: 0.113478, (Pelusios_castaneus: 0.083978, Pelomedusa_subrufa: 0.082730): 0.203344): 0.164830, ((Chelus_fimbriata: 0.085094, (Platemys_platycephala: 0.031114, Mesoclemmys_hogei: 0.000000): 0.036494): 0.007596, (((Elusor_macrurus: 0.000000, (Myuchelys_bellii: 0.031562, Emydura_subglobosa: 0.000045): 0.010328): 0.020245, (Chelodina_parkeri: 0.020516, (Chelodina_rugosa: 0.010028, (Chelodina_pritchardi: 0.000000, (Chelodina_mccordi: 0.000006, (Chelodina_longicollis: 0.000000, Chelodina_expansa: 0.000000): 0.000001): 0.000009): 0.000013): 0.010688): 0.044311): 0.000000, Pseudemydura_umbrina: 0.021628): 0.000000): 0.038856): 0.102807, ((Carettochelys_insculpta: 0.175942, ((Lissemys_scutata: 0.000016, Lissemys_punctata: 0.009439): 0.046470, ((Trionyx_triunguis: 0.039440, (Pelochelys_cantorii: 0.018686, Chitra_indica: 0.019695): 0.023630): 0.017551, ((Rafetus_swinhoei: 0.000052, (Apalone_spinifera: 0.015742, Apalone_ferox: 0.396987): 0.033942): 0.017845, ((Amyda_cartilaginea: 0.000019, Dogania_subplana: 0.043015): 0.000014, (Palea_steindachneri: 0.021545, Pelodiscus_sinensis: 0.079366): 0.000072): 0.021049): 0.000000): 0.011703): 0.148752): 0.004891, (((Sternotherus_carinatus: 0.014273, Kinosternon_leucostomum: 0.015537): 0.143734, (Macrochelys_temminckii: 0.046642, Chelydra_serpentina: 0.000061): 0.059226): 0.000008, (((Platysternon_megacephalum: 0.052640, (Chrysemys_picta: 0.022484, (Trachemys_scripta: 0.010988, Malaclemys_terrapin: 0.022728): 0.000001): 0.032678): 0.000000, (((((Astrochelys_yniphora: 0.010597, Aldabrachelys_gigantea: 0.021546): 0.000000, Psammobates_pardalis: 0.010496): 0.010683, ((((Indotestudo_forstenii: 0.000014, Indotestudo_elongata: 0.000002): 0.032315, Testudo_horsfieldii: 0.020717): 0.000009, (Testudo_graeca: 0.031527, (Testudo_marginata: 0.000003, Testudo_kleinmanni: 0.000030): 0.000000): 0.000000): 0.000000, Malacochersus_tornieri: 0.052494): 0.043174): 0.021450, (Manouria_impressa: 0.010597, Manouria_emys: 0.010195): 0.000048): 0.025330, ((Batagur_trivittata: 0.114453, Geoemyda_spengleri: 0.070916): 0.003424, ((((Notochelys_platynota: 0.030522, (Cyclemys_fusca: 0.000002, ((Cyclemys_tcheponensis: 0.000002, Cyclemys_oldhamii: 0.102236): 0.000002, (Cyclemys_dentata: 0.009861, (Cyclemys_pulchristriata: 0.067145, Cyclemys_atripons: 0.000003): 0.010613): 0.030558): 0.000002): 0.009974): 0.000000, (Heosemys_annandalii: 0.046813, (Heosemys_grandis: 0.011601, Heosemys_depressa: 0.007252): 0.039122): 0.061239): 0.000010, (Sacalia_quadriocellata: 0.021126, Sacalia_bealei: 0.120556): 0.000005): 0.005805, (((((Cuora_flavomarginata: 0.010486, (Cuora_trifasciata: 0.010648, (Cuora_pani: 0.000002, Cuora_aurocapitata: 0.000002): 0.000006): 0.010407): 0.000000, (Cuora_mccordi: 0.000000, (Cuora_galbinifrons: 0.010458, (Cuora_picturata: 0.000000, Cuora_bourreti: 0.000000): 0.000004): 0.010582): 0.010491): 0.000000, Cuora_mouhotii: 0.032016): 0.000000, Cuora_amboinensis: 0.010292): 0.000003, ((Mauremys_leprosa: 0.010263, (((Mauremys_japonica: 0.021057, Mauremys_sinensis: 0.010615): 0.000000, ((Mauremys_reevesii: 0.000000): 0.021570, Mauremys_nigricans: 0.010663): 0.000003): 0.000012, (Mauremys_rivulata: 0.000002, Mauremys_caspica: 0.000002): 0.010390): 0.000002): 0.000000, (Mauremys_mutica: 0.010641, Mauremys_annamensis: 0.044498): 0.000003): 0.021035): 0.026029): 0.013735): 0.230846): 0.029696): 0.000000, (Dermochelys_coriacea: 0.054094, ((Eretmochelys_imbricata: 0.020392, (Caretta_caretta: 0.000033, (Lepidochelys_olivacea: 0.000002, Lepidochelys_kempii: 0.000000): 0.009877): 0.000013): 0.020663, (Chelonia_mydas: 0.000000, Natator_depressa: 0.000027): 0.000000): 0.010318): 0.000004): 0.000026): 0.000000): 0.030254): 0.036446);

**w ratios as labels for TreeView:**

((((((Anolis_carolinensis #0.816184 , Iguana_iguana #0.0176217 ) #0.00103128 , Anguis_fragilis #0.195206 ) #0.0001 , Lacerta_viridis #1.32344 ) #0.0001 , Smaug_warreni #0.19727 ) #0.0001 , (Rena_humilis #0.00675778 , ((Python_regius #612.077 , Boa_constrictor #0.048625 ) #0.0102888 , (Protobothrops_flavoviridis #0.185621 , Dinodon_semicarinatus #0.0788399 ) #0.0001 ) #999 ) #999 ) #0.00630577 , ((Rhea_americana #0.00683938 , (Gallus_gallus #0.00120935 , (Columba_livia #0.0426521 , (Grus_leucogeranus #0.0001 , (Falco_peregrinus #0.00345674 , Pelecanus_occidentalis #999 ) #0.0001 ) #0.0001 ) #0.0001 ) #0.0160462 ) #999 , ((Alligator_mississippiensis #0.00825653 , Caiman_crocodylus #0.0163282 ) #770.582 , (Crocodylus_porosus #0.0597914 , Gavialis_gangeticus #0.0877304 ) #999 ) #999 ) #0.0001 , (((Podocnemis_unifilis #0.0396842 , (Pelusios_castaneus #0.23028 , Pelomedusa_subrufa #0.472737 ) #999 ) #0.172856 , ((Chelus_fimbriata #0.107635 , (Platemys_platycephala #0.0264402 , Mesoclemmys_hogei #0.0001 ) #0.0585884 ) #0.00679529 , (((Elusor_macrurus #0.0001 , (Myuchelys_bellii #0.0786174 , Emydura_subglobosa #0.0001 ) #601.245 ) #999 , (Chelodina_parkeri #999 , (Chelodina_rugosa #0.0406579 , (Chelodina_pritchardi #0.0001 , (Chelodina_mccordi #0.0001 , (Chelodina_longicollis #0.0001 , Chelodina_expansa #0.0001 ) #0.0001 ) #0.0001 ) #0.0001 ) #0.0160535 ) #0.000940759 ) #0.0001 , Pseudemydura_umbrina #0.0105562 ) #0.0001 ) #0.000824768 ) #999 , ((Carettochelys_insculpta #0.0673626 , ((Lissemys_scutata #0.0001 , Lissemys_punctata #0.0813704 ) #0.0767309 , ((Trionyx_triunguis #0.0801599 , (Pelochelys_cantorii #0.0734624 , Chitra_indica #0.0696884 ) #0.0579781 ) #999 , ((Rafetus_swinhoei #0.0001 , (Apalone_spinifera #999 , Apalone_ferox #0.0085455 ) #0.132086 ) #999 , ((Amyda_cartilaginea #0.0001 , Dogania_subplana #0.152475 ) #0.0001 , (Palea_steindachneri #0.0631739 , Pelodiscus_sinensis #0.420423 ) #0.0001 ) #0.356968 ) #0.0001 ) #999 ) #0.646797 ) #504.039 , (((Sternotherus_carinatus #672.214 , Kinosternon_leucostomum #0.0406891 ) #0.0606959 , (Macrochelys_temminckii #999 , Chelydra_serpentina #0.0001 ) #0.151795 ) #0.0001 , (((Platysternon_megacephalum #0.158497 , (Chrysemys_picta #0.0883484 , (Trachemys_scripta #999 , Malaclemys_terrapin #0.167619 ) #0.0001 ) #0.0258604 ) #0.0001 , (((((Astrochelys_yniphora #0.038422 , Aldabrachelys_gigantea #0.651725 ) #0.0001 , Psammobates_pardalis #0.0195705 ) #999 , ((((Indotestudo_forstenii #0.0001 , Indotestudo_elongata #0.0001 ) #0.143013 , Testudo_horsfieldii #0.101081 ) #0.0001 , (Testudo_graeca #0.116985 , (Testudo_marginata #0.0001 , Testudo_kleinmanni #0.0001 ) #0.0001 ) #0.0001 ) #0.0001 , Malacochersus_tornieri #0.0793119 ) #0.261167 ) #999 , (Manouria_impressa #772.939 , Manouria_emys #0.0318999 ) #0.0001 ) #999 , ((Batagur_trivittata #0.28681 , Geoemyda_spengleri #3.29143 ) #0.0580658 , ((((Notochelys_platynota #0.132895 , (Cyclemys_fusca #53.9193 , ((Cyclemys_tcheponensis #53.9306 , Cyclemys_oldhamii #0.165948 ) #53.0082 , (Cyclemys_dentata #999 , (Cyclemys_pulchristriata #0.118455 , Cyclemys_atripons #0.0001 ) #0.081983 ) #0.115224 ) #52.8225 ) #0.0191154 ) #0.0001 , (Heosemys_annandalii #0.171247 , (Heosemys_grandis #0.268734 , Heosemys_depressa #0.0646469 ) #0.690863 ) #0.381187 ) #0.0001 , (Sacalia_quadriocellata #0.220011 , Sacalia_bealei #0.33602 ) #0.0001 ) #999 , (((((Cuora_flavomarginata #0.0587875 , (Cuora_trifasciata #0.156825 , (Cuora_pani #30.4128 , Cuora_aurocapitata #30.7841 ) #0.0001 ) #0.22503 ) #0.0001 , (Cuora_mccordi #0.0001 , (Cuora_galbinifrons #0.247377 , (Cuora_picturata #0.0001 , Cuora_bourreti #0.0001 ) #0.0001 ) #999 ) #0.115941 ) #0.0001 , Cuora_mouhotii #0.133595 ) #0.0001 , Cuora_amboinensis #0.490973 ) #0.0001 , ((Mauremys_leprosa #0.0700453 , (((Mauremys_japonica #0.746686 , Mauremys_sinensis #0.142659 ) #0.0001 , ((Mauremys_reevesii #0.0001 , NC_015101.1 #0.0001 ) #0.32366 , Mauremys_nigricans #0.211516 ) #0.0001 ) #0.0001 , (Mauremys_rivulata #45.2965 , Mauremys_caspica #70.612 ) #0.102314 ) #0.0001 ) #0.0001 , (Mauremys_mutica #0.0434368 , Mauremys_annamensis #0.258972 ) #0.0001 ) #0.749413 ) #0.144452 ) #0.17489 ) #0.733671 ) #0.110892 ) #0.0001 , (Dermochelys_coriacea #0.0983128 , ((Eretmochelys_imbricata #1.63034 , (Caretta_caretta #0.0001 , (Lepidochelys_olivacea #0.0001 , Lepidochelys_kempii #0.0001 ) #957.26 ) #0.0001 ) #0.104746 , (Chelonia_mydas #0.0001 , Natator_depressa #0.0001 ) #0.0001 ) #0.0454811 ) #0.0001 ) #0.0001 ) #0.0001 ) #0.00227946 ) #999 );

**ND5**

**dS tree:**

((((((Anolis_carolinensis: 11.808194, Iguana_iguana: 6.234916): 0.000051, Anguis_fragilis: 7.978420): 0.000235, Lacerta_viridis: 4.773271): 0.000275, Smaug_warreni: 6.526756): 0.000209, (Rena_humilis: 27.411173, ((Python_regius: 23.005273, Boa_constrictor: 66.427890): 0.000044, (Protobothrops_flavoviridis: 4.727156, Dinodon_semicarinatus: 7.050650): 0.000569): 0.000433): 0.000136): 0.000183, ((Rhea_americana: 1.112669, (Gallus_gallus: 1.637816, (Columba_livia: 0.883287, (Grus_leucogeranus: 0.924284, (Falco_peregrinus: 1.486780, Pelecanus_occidentalis: 0.866889): 0.000005): 0.000013): 0.362332): 0.580709): 2.321086, ((Alligator_mississippiensis: 0.281512, Caiman_crocodylus: 1.288688): 5.441516, (Crocodylus_porosus: 1.113618, Gavialis_gangeticus: 1.830659): 3.281873): 0.000188): 0.109882, (((Podocnemis_unifilis: 14.660843, (Pelusios_castaneus: 1.101284, Pelomedusa_subrufa: 0.100549): 2.749646): 0.001109, ((Chelus_fimbriata: 1.069436, (Platemys_platycephala: 0.328446, Mesoclemmys_hogei: 0.613614): 0.528897): 0.509643, (((Elusor_macrurus: 0.246738, (Myuchelys_bellii: 0.146377, Emydura_subglobosa: 0.254940): 0.174200): 0.327317, (Chelodina_parkeri: 0.325725, (Chelodina_rugosa: 0.217805, (Chelodina_pritchardi: 0.108859, (Chelodina_mccordi: 0.064932, (Chelodina_longicollis: 0.000005, Chelodina_expansa: 0.015226): 0.047933): 0.041644): 0.219441): 0.298034): 0.898966): 0.264786, Pseudemydura_umbrina: 1.074306): 0.359721): 0.470261): 0.000100, ((Carettochelys_insculpta: 2.836321, ((Lissemys_scutata: 4.708076, Lissemys_punctata: 0.000099): 0.000027, ((Trionyx_triunguis: 0.651103, (Pelochelys_cantorii: 0.287429, Chitra_indica: 0.384804): 0.188553): 1.096383, ((Rafetus_swinhoei: 1.437164, (Apalone_spinifera: 0.063705, Apalone_ferox: 0.285468): 0.337132): 0.795029, ((Amyda_cartilaginea: 1.094570, Dogania_subplana: 0.000102): 0.000026, (Palea_steindachneri: 0.507434, Pelodiscus_sinensis: 0.562032): 0.615452): 0.000038): 0.000005): 0.000018): 2.138228): 0.000053, (((Sternotherus_carinatus: 0.271297, Kinosternon_leucostomum: 0.198474): 0.874630, (Macrochelys_temminckii: 0.233229, Chelydra_serpentina: 0.715127): 0.552093): 0.000343, (((Platysternon_megacephalum: 0.935663, (Chrysemys_picta: 0.289135, (Trachemys_scripta: 0.123072, Malaclemys_terrapin: 0.078281): 0.160833): 0.554092): 0.024964, (((((Astrochelys_yniphora: 0.360973, Aldabrachelys_gigantea: 0.244101): 0.125466, Psammobates_pardalis: 0.406815): 0.091110, ((((Indotestudo_forstenii: 0.094055, Indotestudo_elongata: 0.133465): 0.178932, Testudo_horsfieldii: 0.318356): 0.000031, (Testudo_graeca: 0.223479, (Testudo_marginata: 0.123931, Testudo_kleinmanni: 0.165234): 0.108603): 0.151922): 0.039053, Malacochersus_tornieri: 0.284141): 0.183529): 0.109061, (Manouria_impressa: 0.217664, Manouria_emys: 0.230095): 0.127490): 0.130608, ((Batagur_trivittata: 0.522279, Geoemyda_spengleri: 0.489899): 0.081252, ((((Notochelys_platynota: 0.373405, (Cyclemys_fusca: 0.175940, ((Cyclemys_tcheponensis: 0.004922, Cyclemys_oldhamii: 0.021561): 0.164690, (Cyclemys_dentata: 0.048945, (Cyclemys_pulchristriata: 0.039399, Cyclemys_atripons: 0.028219): 0.086919): 0.080299): 0.079666): 0.042217): 0.040280, (Heosemys_annandalii: 0.250848, (Heosemys_grandis: 0.169961, Heosemys_depressa: 0.193821): 0.049374): 0.235147): 0.052067, (Sacalia_quadriocellata: 0.219524, Sacalia_bealei: 0.195781): 0.022867): 0.109177, (((((Cuora_flavomarginata: 0.114677, (Cuora_trifasciata: 0.041127, (Cuora_pani: 0.007019, Cuora_aurocapitata: 0.117185): 0.021591): 0.053433): 0.036905, (Cuora_mccordi: 0.128119, (Cuora_galbinifrons: 0.056786, (Cuora_picturata: 0.000000, Cuora_bourreti: 0.000000): 0.073838): 0.047626): 0.032182): 0.000011, Cuora_mouhotii: 0.175010): 0.033020, Cuora_amboinensis: 0.278443): 0.064798, ((Mauremys_leprosa: 0.223462, (((Mauremys_japonica: 0.113209, Mauremys_sinensis: 0.061199): 0.022085, ((Mauremys_reevesii: 0.005136, NC_015101.1: 0.002327): 0.090369, Mauremys_nigricans: 0.087138): 0.014155): 0.072577, (Mauremys_rivulata: 0.000000, Mauremys_caspica: 0.000000): 0.144148): 0.006123): 0.008879, (Mauremys_mutica: 0.094696, Mauremys_annamensis: 0.111168): 0.070943): 0.088554): 0.084274): 0.110568): 0.103633): 0.196719): 0.125529, (Dermochelys_coriacea: 0.602482, ((Eretmochelys_imbricata: 0.239499, (Caretta_caretta: 0.153756, (Lepidochelys_olivacea: 0.027268, Lepidochelys_kempii: 0.030456): 0.143050): 0.090661): 0.062409, (Chelonia_mydas: 0.234910, Natator_depressa: 0.242758): 0.072394): 0.202527): 0.175665): 0.066268): 0.279495): 1.008321): 6.614473);

**dN tree:**

((((((Anolis_carolinensis: 0.092297, Iguana_iguana: 0.089775): 0.051417, Anguis_fragilis: 0.141908): 0.019475, Lacerta_viridis: 0.154717): 0.024241, Smaug_warreni: 0.139843): 0.020862, (Rena_humilis: 0.213043, ((Python_regius: 0.065322, Boa_constrictor: 0.092420): 0.032242, (Protobothrops_flavoviridis: 0.105740, Dinodon_semicarinatus: 0.091536): 0.063293): 0.087635): 0.136246): 0.041565, ((Rhea_americana: 0.035261, (Gallus_gallus: 0.074811, (Columba_livia: 0.047996, (Grus_leucogeranus: 0.026870, (Falco_peregrinus: 0.064201, Pelecanus_occidentalis: 0.035830): 0.004168): 0.004096): 0.020850): 0.014838): 0.105654, ((Alligator_mississippiensis: 0.072000, Caiman_crocodylus: 0.147585): 0.033417, (Crocodylus_porosus: 0.103604, Gavialis_gangeticus: 0.104474): 0.081114): 0.188175): 0.023360, (((Podocnemis_unifilis: 0.195188, (Pelusios_castaneus: 0.042724, Pelomedusa_subrufa: 0.016684): 0.188237): 0.132645, ((Chelus_fimbriata: 0.060282, (Platemys_platycephala: 0.027592, Mesoclemmys_hogei: 0.032229): 0.020158): 0.027762, (((Elusor_macrurus: 0.009573, (Myuchelys_bellii: 0.011648, Emydura_subglobosa: 0.016436): 0.006483): 0.038437, (Chelodina_parkeri: 0.033129, (Chelodina_rugosa: 0.017854, (Chelodina_pritchardi: 0.008101, (Chelodina_mccordi: 0.006642, (Chelodina_longicollis: 0.000000, Chelodina_expansa: 0.000837): 0.004291): 0.004624): 0.008939): 0.008370): 0.052055): 0.010739, Pseudemydura_umbrina: 0.069324): 0.012319): 0.161899): 0.094912, ((Carettochelys_insculpta: 0.089290, ((Lissemys_scutata: 0.086204, Lissemys_punctata: 0.034863): 0.005086, ((Trionyx_triunguis: 0.029588, (Pelochelys_cantorii: 0.019208, Chitra_indica: 0.031839): 0.010179): 0.015398, ((Rafetus_swinhoei: 0.070777, (Apalone_spinifera: 0.010703, Apalone_ferox: 0.012570): 0.019892): 0.001832, ((Amyda_cartilaginea: 0.024133, Dogania_subplana: 0.036702): 0.004063, (Palea_steindachneri: 0.021911, Pelodiscus_sinensis: 0.030956): 0.003751): 0.007288): 0.000583): 0.002778): 0.052841): 0.015864, (((Sternotherus_carinatus: 0.016667, Kinosternon_leucostomum: 0.019802): 0.080523, (Macrochelys_temminckii: 0.032655, Chelydra_serpentina: 0.035970): 0.039014): 0.045529, (((Platysternon_megacephalum: 0.155992, (Chrysemys_picta: 0.065471, (Trachemys_scripta: 0.028477, Malaclemys_terrapin: 0.017076): 0.064719): 0.126116): 0.037550, (((((Astrochelys_yniphora: 0.014745, Aldabrachelys_gigantea: 0.020014): 0.009745, Psammobates_pardalis: 0.050202): 0.004612, ((((Indotestudo_forstenii: 0.011094, Indotestudo_elongata: 0.007764): 0.020511, Testudo_horsfieldii: 0.013094): 0.004820, (Testudo_graeca: 0.013418, (Testudo_marginata: 0.016961, Testudo_kleinmanni: 0.010833): 0.002539): 0.009001): 0.002724, Malacochersus_tornieri: 0.029565): 0.008349): 0.005769, (Manouria_impressa: 0.012124, Manouria_emys: 0.016240): 0.009806): 0.005518, ((Batagur_trivittata: 0.035419, Geoemyda_spengleri: 0.034974): 0.006928, ((((Notochelys_platynota: 0.048242, (Cyclemys_fusca: 0.037366, ((Cyclemys_tcheponensis: 0.000000, Cyclemys_oldhamii: 0.012727): 0.012770, (Cyclemys_dentata: 0.006054, (Cyclemys_pulchristriata: 0.002433, Cyclemys_atripons: 0.002713): 0.000947): 0.006146): 0.005360): 0.003584): 0.003278, (Heosemys_annandalii: 0.022073, (Heosemys_grandis: 0.015697, Heosemys_depressa: 0.009166): 0.004186): 0.008353): 0.000005, (Sacalia_quadriocellata: 0.013519, Sacalia_bealei: 0.010177): 0.010272): 0.006506, (((((Cuora_flavomarginata: 0.011873, (Cuora_trifasciata: 0.007919, (Cuora_pani: 0.002658, Cuora_aurocapitata: 0.016959): 0.005991): 0.002376): 0.002491, (Cuora_mccordi: 0.008666, (Cuora_galbinifrons: 0.010321, (Cuora_picturata: 0.000002, Cuora_bourreti: 0.000002): 0.005975): 0.007701): 0.006744): 0.000992, Cuora_mouhotii: 0.010455): 0.001686, Cuora_amboinensis: 0.015516): 0.003934, ((Mauremys_leprosa: 0.020802, (((Mauremys_japonica: 0.005938, Mauremys_sinensis: 0.011032): 0.000002, ((Mauremys_reevesii: 0.000001): 0.006802, Mauremys_nigricans: 0.008528): 0.000001): 0.002597, (Mauremys_rivulata: 0.000002, Mauremys_caspica: 0.000002): 0.009401): 0.001468): 0.000001, (Mauremys_mutica: 0.011963, Mauremys_annamensis: 0.013816): 0.002432): 0.006449): 0.007412): 0.005537): 0.016437): 0.013710): 0.004580, (Dermochelys_coriacea: 0.030903, ((Eretmochelys_imbricata: 0.012481, (Caretta_caretta: 0.012039, (Lepidochelys_olivacea: 0.004947, Lepidochelys_kempii: 0.000003): 0.015003): 0.006263): 0.006630, (Chelonia_mydas: 0.011332, Natator_depressa: 0.009319): 0.003230): 0.004441): 0.013993): 0.002380): 0.018156): 0.047040): 0.025030);

**w ratios as labels for TreeView:**

((((((Anolis_carolinensis #0.00781638 , Iguana_iguana #0.0143988 ) #999 , Anguis_fragilis #0.0177865 ) #82.794 , Lacerta_viridis #0.0324132 ) #88.0201 , Smaug_warreni #0.0214261 ) #99.8493 , (Rena_humilis #0.00777211 , ((Python_regius #0.00283946 , Boa_constrictor #0.00139128 ) #741.081 , (Protobothrops_flavoviridis #0.0223686 , Dinodon_semicarinatus #0.0129826 ) #111.333 ) #202.327 ) #999 ) #226.563 , ((Rhea_americana #0.0316909 , (Gallus_gallus #0.0456771 , (Columba_livia #0.0543376 , (Grus_leucogeranus #0.0290709 , (Falco_peregrinus #0.0431811 , Pelecanus_occidentalis #0.0413315 ) #881.943 ) #318.045 ) #0.0575452 ) #0.0255508 ) #0.0455194 , ((Alligator_mississippiensis #0.25576 , Caiman_crocodylus #0.114523 ) #0.00614111 , (Crocodylus_porosus #0.0930334 , Gavialis_gangeticus #0.0570692 ) #0.0247158 ) #999 ) #0.21259 , (((Podocnemis_unifilis #0.0133135 , (Pelusios_castaneus #0.0387943 , Pelomedusa_subrufa #0.165925 ) #0.0684586 ) #119.598 , ((Chelus_fimbriata #0.0563677 , (Platemys_platycephala #0.0840089 , Mesoclemmys_hogei #0.052523 ) #0.0381127 ) #0.0544742 , (((Elusor_macrurus #0.0387962 , (Myuchelys_bellii #0.0795775 , Emydura_subglobosa #0.0644717 ) #0.0372136 ) #0.117429 , (Chelodina_parkeri #0.10171 , (Chelodina_rugosa #0.0819742 , (Chelodina_pritchardi #0.0744185 , (Chelodina_mccordi #0.102296 , (Chelodina_longicollis #0.0001 , Chelodina_expansa #0.0549436 ) #0.0895161 ) #0.111048 ) #0.0407354 ) #0.028085 ) #0.0579049 ) #0.0405586 , Pseudemydura_umbrina #0.064529 ) #0.0342467 ) #0.344275 ) #945.128 , ((Carettochelys_insculpta #0.0314808 , ((Lissemys_scutata #0.0183098 , Lissemys_punctata #352.216 ) #186.95 , ((Trionyx_triunguis #0.0454427 , (Pelochelys_cantorii #0.0668266 , Chitra_indica #0.0827407 ) #0.0539874 ) #0.0140447 , ((Rafetus_swinhoei #0.0492477 , (Apalone_spinifera #0.168006 , Apalone_ferox #0.0440313 ) #0.0590021 ) #0.00230424 , ((Amyda_cartilaginea #0.0220478 , Dogania_subplana #360.727 ) #158.529 , (Palea_steindachneri #0.0431796 , Pelodiscus_sinensis #0.0550783 ) #0.00609538 ) #192.855 ) #110.676 ) #152.811 ) #0.0247126 ) #297.154 , (((Sternotherus_carinatus #0.0614343 , Kinosternon_leucostomum #0.0997726 ) #0.0920648 , (Macrochelys_temminckii #0.140013 , Chelydra_serpentina #0.0502985 ) #0.0706654 ) #132.798 , (((Platysternon_megacephalum #0.166718 , (Chrysemys_picta #0.226437 , (Trachemys_scripta #0.231388 , Malaclemys_terrapin #0.218132 ) #0.402398 ) #0.227609 ) #1.50417 , (((((Astrochelys_yniphora #0.0408491 , Aldabrachelys_gigantea #0.0819926 ) #0.0776685 , Psammobates_pardalis #0.123402 ) #0.0506234 , ((((Indotestudo_forstenii #0.117956 , Indotestudo_elongata #0.0581687 ) #0.114629 , Testudo_horsfieldii #0.041131 ) #157.974 , (Testudo_graeca #0.0600418 , (Testudo_marginata #0.136857 , Testudo_kleinmanni #0.0655628 ) #0.0233758 ) #0.059251 ) #0.0697622 , Malacochersus_tornieri #0.104051 ) #0.0454939 ) #0.052897 , (Manouria_impressa #0.0556999 , Manouria_emys #0.0705774 ) #0.0769136 ) #0.042249 , ((Batagur_trivittata #0.0678153 , Geoemyda_spengleri #0.071391 ) #0.0852684 , ((((Notochelys_platynota #0.129194 , (Cyclemys_fusca #0.212377 , ((Cyclemys_tcheponensis #0.0001 , Cyclemys_oldhamii #0.590279 ) #0.0775393 , (Cyclemys_dentata #0.123683 , (Cyclemys_pulchristriata #0.0617634 , Cyclemys_atripons #0.0961521 ) #0.0108908 ) #0.0765406 ) #0.0672855 ) #0.0848913 ) #0.081377 , (Heosemys_annandalii #0.0879944 , (Heosemys_grandis #0.0923558 , Heosemys_depressa #0.0472887 ) #0.0847807 ) #0.0355218 ) #0.0001 , (Sacalia_quadriocellata #0.0615838 , Sacalia_bealei #0.0519837 ) #0.449205 ) #0.0595907 , (((((Cuora_flavomarginata #0.103538 , (Cuora_trifasciata #0.19254 , (Cuora_pani #0.378676 , Cuora_aurocapitata #0.144718 ) #0.277462 ) #0.044458 ) #0.0675088 , (Cuora_mccordi #0.0676413 , (Cuora_galbinifrons #0.181755 , (Cuora_picturata #6.36588 , Cuora_bourreti #6.11205 ) #0.080918 ) #0.161698 ) #0.209568 ) #86.3282 , Cuora_mouhotii #0.0597379 ) #0.051062 , Cuora_amboinensis #0.0557241 ) #0.0607143 , ((Mauremys_leprosa #0.0930918 , (((Mauremys_japonica #0.0524508 , Mauremys_sinensis #0.180258 ) #0.0001 , ((Mauremys_reevesii #0.0001 , NC_015101.1 #0.0001 ) #0.0752678 , Mauremys_nigricans #0.0978676 ) #0.0001 ) #0.0357851 , (Mauremys_rivulata #59.6904 , Mauremys_caspica #4.76712 ) #0.0652189 ) #0.239806 ) #0.0001 , (Mauremys_mutica #0.126335 , Mauremys_annamensis #0.124277 ) #0.034288 ) #0.0728243 ) #0.0879542 ) #0.0500772 ) #0.158607 ) #0.0696951 ) #0.0364831 , (Dermochelys_coriacea #0.051293 , ((Eretmochelys_imbricata #0.0521138 , (Caretta_caretta #0.0783 , (Lepidochelys_olivacea #0.181426 , Lepidochelys_kempii #0.0001 ) #0.104876 ) #0.0690829 ) #0.106227 , (Chelonia_mydas #0.0482389 , Natator_depressa #0.038389 ) #0.0446186 ) #0.021927 ) #0.0796564 ) #0.0359104 ) #0.0649602 ) #0.0466523 ) #0.00378416 );

**ND6**

**dS tree:**

((((((Anolis_carolinensis: 61.443784, Iguana_iguana: 4.398801): 0.000317, Anguis_fragilis: 37.952290): 0.000314, Lacerta_viridis: 61.458288): 0.000327, Smaug_warreni: 3.269831): 0.000005, (Rena_humilis: 31.281107, ((Python_regius: 6.345595, Boa_constrictor: 8.982085): 61.721002, (Protobothrops_flavoviridis: 21.912176, Dinodon_semicarinatus: 1.861384): 61.758857): 0.002880): 0.000893): 27.929275, ((Rhea_americana: 10.684063, (Gallus_gallus: 3.048545, (Columba_livia: 59.487163, (Grus_leucogeranus: 0.000214, (Falco_peregrinus: 4.391350, Pelecanus_occidentalis: 0.000229): 1.667683): 0.000059): 0.000068): 2.117258): 9.924116, ((Alligator_mississippiensis: 1.611467, Caiman_crocodylus: 0.292365): 0.719821, (Crocodylus_porosus: 2.847490, Gavialis_gangeticus: 1.270376): 1.146133): 10.591319): 0.000001, (((Podocnemis_unifilis: 61.598580, (Pelusios_castaneus: 0.488371, Pelomedusa_subrufa: 0.500005): 0.000551): 4.609294, ((Chelus_fimbriata: 1.216294, (Platemys_platycephala: 0.353251, Mesoclemmys_hogei: 0.663741): 0.000081): 0.798021, (((Elusor_macrurus: 0.236258, (Myuchelys_bellii: 0.082295, Emydura_subglobosa: 0.143936): 0.062483): 0.602371, (Chelodina_parkeri: 0.476490, (Chelodina_rugosa: 0.000036, (Chelodina_pritchardi: 0.049990, (Chelodina_mccordi: 0.073833, (Chelodina_longicollis: 0.000000, Chelodina_expansa: 0.000000): 0.029390): 0.042435): 0.291786): 0.000005): 0.833554): 0.000005, Pseudemydura_umbrina: 0.779357): 0.396866): 0.000177): 0.181505, ((Carettochelys_insculpta: 5.732087, ((Lissemys_scutata: 0.497079, Lissemys_punctata: 0.000021): 1.171548, ((Trionyx_triunguis: 0.134953, (Pelochelys_cantorii: 0.068449, Chitra_indica: 0.262213): 0.171497): 0.069559, ((Rafetus_swinhoei: 0.235456, (Apalone_spinifera: 0.019506, Apalone_ferox: 0.202458): 0.220687): 0.000025, ((Amyda_cartilaginea: 0.512775, Dogania_subplana: 0.269192): 0.076457, (Palea_steindachneri: 0.372934, Pelodiscus_sinensis: 0.370568): 0.016417): 0.302378): 0.000005): 0.126018): 0.690262): 0.000464, (((Sternotherus_carinatus: 0.000100, Kinosternon_leucostomum: 0.438103): 1.340427, (Macrochelys_temminckii: 0.127051, Chelydra_serpentina: 0.353360): 0.000175): 0.000064, (((Platysternon_megacephalum: 1.557909, (Chrysemys_picta: 0.296607, (Trachemys_scripta: 0.129188, Malaclemys_terrapin: 0.139371): 0.196557): 0.406032): 0.268515, (((((Astrochelys_yniphora: 0.099575, Aldabrachelys_gigantea: 0.343768): 0.000046, Psammobates_pardalis: 0.214711): 0.000080, ((((Indotestudo_forstenii: 0.223815, Indotestudo_elongata: 0.000023): 0.210518, Testudo_horsfieldii: 0.128718): 0.000109, (Testudo_graeca: 0.354638, (Testudo_marginata: 0.043253, Testudo_kleinmanni: 0.405644): 0.175798): 0.000005): 0.376776, Malacochersus_tornieri: 0.536743): 0.131006): 0.280645, (Manouria_impressa: 0.289189, Manouria_emys: 0.090514): 0.093864): 0.351027, ((Batagur_trivittata: 0.521299, Geoemyda_spengleri: 0.339808): 0.205157, ((((Notochelys_platynota: 0.551132, (Cyclemys_fusca: 0.383955, ((Cyclemys_tcheponensis: 0.000005, Cyclemys_oldhamii: 0.011921): 0.083948, (Cyclemys_dentata: 0.102944, (Cyclemys_pulchristriata: 0.014126, Cyclemys_atripons: 0.026911): 0.000037): 0.067026): 0.000005): 0.044722): 0.028754, (Heosemys_annandalii: 0.348073, (Heosemys_grandis: 0.094167, Heosemys_depressa: 0.070495): 0.052480): 0.065782): 0.049231, (Sacalia_quadriocellata: 0.127753, Sacalia_bealei: 0.055663): 0.258471): 0.000035, (((((Cuora_flavomarginata: 0.073654, (Cuora_trifasciata: 0.000019, (Cuora_pani: 0.057364, Cuora_aurocapitata: 1.230119): 0.000001): 0.013734): 0.028917, (Cuora_mccordi: 0.098614, (Cuora_galbinifrons: 0.097367, (Cuora_picturata: 0.000005, Cuora_bourreti: 0.000005): 0.079392): 0.000005): 0.053625): 0.000005, Cuora_mouhotii: 0.055853): 0.000005, Cuora_amboinensis: 0.139109): 0.058529, ((Mauremys_leprosa: 0.095662, (((Mauremys_japonica: 0.025067, Mauremys_sinensis: 0.061716): 0.010627, ((Mauremys_reevesii: 0.000001, NC_015101.1: 0.011536): 0.067452, Mauremys_nigricans: 0.038514): 0.023803): 0.068870, (Mauremys_rivulata: 0.000001, Mauremys_caspica: 0.000001): 0.175977): 0.000005): 0.000005, (Mauremys_mutica: 0.164902, Mauremys_annamensis: 0.113063): 0.071065): 0.005913): 0.253578): 0.000040): 0.000390): 1.241508): 0.000005, (Dermochelys_coriacea: 0.921691, ((Eretmochelys_imbricata: 0.219560, (Caretta_caretta: 0.000005, (Lepidochelys_olivacea: 0.004658, Lepidochelys_kempii: 0.035653): 0.094437): 0.067477): 0.249744, (Chelonia_mydas: 0.333692, Natator_depressa: 0.064292): 0.000005): 0.188407): 0.650230): 0.050390): 0.287567): 1.309942): 17.006445);

**dN tree:**

((((((Anolis_carolinensis: 0.122588, Iguana_iguana: 0.050761): 0.032793, Anguis_fragilis: 0.168746): 0.034317, Lacerta_viridis: 0.117229): 0.034403, Smaug_warreni: 0.190016): 0.000000, (Rena_humilis: 0.386050, ((Python_regius: 0.146453, Boa_constrictor: 0.127967): 0.020162, (Protobothrops_flavoviridis: 0.167482, Dinodon_semicarinatus: 0.207839): 0.006176): 0.776003): 0.140585): 0.063626, ((Rhea_americana: 0.068399, (Gallus_gallus: 0.043984, (Columba_livia: 0.033978, (Grus_leucogeranus: 0.041289, (Falco_peregrinus: 0.067525, Pelecanus_occidentalis: 0.017795): 0.011040): 0.005446): 0.005157): 0.000212): 0.103271, ((Alligator_mississippiensis: 0.105819, Caiman_crocodylus: 0.175230): 0.048603, (Crocodylus_porosus: 0.111560, Gavialis_gangeticus: 0.073794): 0.102557): 0.606808): 0.000001, (((Podocnemis_unifilis: 0.065394, (Pelusios_castaneus: 0.119665, Pelomedusa_subrufa: 0.062004): 0.121133): 0.127368, ((Chelus_fimbriata: 0.047081, (Platemys_platycephala: 0.013573, Mesoclemmys_hogei: 0.048899): 0.011366): 0.015412, (((Elusor_macrurus: 0.000024, (Myuchelys_bellii: 0.008225, Emydura_subglobosa: 0.012659): 0.000006): 0.036497, (Chelodina_parkeri: 0.026655, (Chelodina_rugosa: 0.008343, (Chelodina_pritchardi: 0.008503, (Chelodina_mccordi: 0.008677, (Chelodina_longicollis: 0.000002, Chelodina_expansa: 0.000002): 0.000003): 0.004329): 0.000029): 0.000000): 0.011148): 0.000000, Pseudemydura_umbrina: 0.043513): 0.009678): 0.039158): 0.133535, ((Carettochelys_insculpta: 0.225104, ((Lissemys_scutata: 0.035006, Lissemys_punctata: 0.004213): 0.066873, ((Trionyx_triunguis: 0.051232, (Pelochelys_cantorii: 0.017771, Chitra_indica: 0.028468): 0.000017): 0.000007, ((Rafetus_swinhoei: 0.038901, (Apalone_spinifera: 0.000002, Apalone_ferox: 0.020660): 0.012544): 0.006836, ((Amyda_cartilaginea: 0.016901, Dogania_subplana: 0.013001): 0.008877, (Palea_steindachneri: 0.030083, Pelodiscus_sinensis: 0.016561): 0.000002): 0.009156): 0.000000): 0.011780): 0.047006): 0.080574, (((Sternotherus_carinatus: 0.024143, Kinosternon_leucostomum: 0.000044): 0.288248, (Macrochelys_temminckii: 0.022662, Chelydra_serpentina: 0.024822): 0.073045): 0.013634, (((Platysternon_megacephalum: 0.042006, (Chrysemys_picta: 0.006704, (Trachemys_scripta: 0.012122, Malaclemys_terrapin: 0.014903): 0.000020): 0.034144): 0.000027, (((((Astrochelys_yniphora: 0.003639, Aldabrachelys_gigantea: 0.021966): 0.014027, Psammobates_pardalis: 0.017211): 0.006475, ((((Indotestudo_forstenii: 0.029978, Indotestudo_elongata: 0.004864): 0.025726, Testudo_horsfieldii: 0.023212): 0.008400, (Testudo_graeca: 0.018153, (Testudo_marginata: 0.004310, Testudo_kleinmanni: 0.004257): 0.004316): 0.000000): 0.000038, Malacochersus_tornieri: 0.061848): 0.035823): 0.021996, (Manouria_impressa: 0.029048, Manouria_emys: 0.020102): 0.008237): 0.032967, ((Batagur_trivittata: 0.075022, Geoemyda_spengleri: 0.042596): 0.000021, ((((Notochelys_platynota: 0.056413, (Cyclemys_fusca: 0.000038, ((Cyclemys_tcheponensis: 0.000000, Cyclemys_oldhamii: 0.000001): 0.012600, (Cyclemys_dentata: 0.000010, (Cyclemys_pulchristriata: 0.016932, Cyclemys_atripons: 0.000003): 0.004130): 0.000007): 0.000000): 0.004780): 0.000003, (Heosemys_annandalii: 0.121026, (Heosemys_grandis: 0.003617, Heosemys_depressa: 0.021276): 0.005417): 0.008269): 0.000005, (Sacalia_quadriocellata: 0.019322, Sacalia_bealei: 0.023780): 0.023026): 0.007770, (((((Cuora_flavomarginata: 0.008338, (Cuora_trifasciata: 0.008498, (Cuora_pani: 0.008469, Cuora_aurocapitata: 0.468776): 0.000002): 0.004087): 0.000003, (Cuora_mccordi: 0.012418, (Cuora_galbinifrons: 0.004135, (Cuora_picturata: 0.000000, Cuora_bourreti: 0.000000): 0.004121): 0.000000): 0.000005): 0.000000, Cuora_mouhotii: 0.029419): 0.000000, Cuora_amboinensis: 0.021162): 0.004197, ((Mauremys_leprosa: 0.021238, (((Mauremys_japonica: 0.056493, Mauremys_sinensis: 0.029903): 0.000001, ((Mauremys_reevesii: 0.000001, NC_015101.1: 0.000001): 0.008547, Mauremys_nigricans: 0.000004): 0.007400): 0.031709, (Mauremys_rivulata: 0.000002, Mauremys_caspica: 0.000002): 0.008385): 0.000000): 0.000000, (Mauremys_mutica: 0.008443, Mauremys_annamensis: 0.008481): 0.000007): 0.004125): 0.016983): 0.011258): 0.039620): 0.027258): 0.000000, (Dermochelys_coriacea: 0.035440, ((Eretmochelys_imbricata: 0.103569, (Caretta_caretta: 0.000000, (Lepidochelys_olivacea: 0.000000, Lepidochelys_kempii: 0.008690): 0.004374): 0.013147): 0.000025, (Chelonia_mydas: 0.004476, Natator_depressa: 0.004304): 0.000000): 0.040384): 0.039824): 0.009340): 0.041420): 0.000131): 0.064050);

**w ratios as labels for TreeView:**

((((((Anolis_carolinensis #0.00199512 , Iguana_iguana #0.0115396 ) #103.319 , Anguis_fragilis #0.00444627 ) #109.311 , Lacerta_viridis #0.00190746 ) #105.364 , Smaug_warreni #0.0581118 ) #0.0001 , (Rena_humilis #0.0123413 , ((Python_regius #0.0230795 , Boa_constrictor #0.014247 ) #0.000326672 , (Protobothrops_flavoviridis #0.00764332 , Dinodon_semicarinatus #0.111658 ) #0.0001 ) #269.425 ) #157.432 ) #0.00227811 , ((Rhea_americana #0.00640195 , (Gallus_gallus #0.014428 , (Columba_livia #0.000571177 , (Grus_leucogeranus #192.65 , (Falco_peregrinus #0.0153768 , Pelecanus_occidentalis #77.6629 ) #0.00662024 ) #91.8921 ) #75.7058 ) #0.0001 ) #0.0104061 , ((Alligator_mississippiensis #0.0656664 , Caiman_crocodylus #0.599353 ) #0.0675204 , (Crocodylus_porosus #0.0391784 , Gavialis_gangeticus #0.0580884 ) #0.0894811 ) #0.057293 ) #1.37383 , (((Podocnemis_unifilis #0.00106162 , (Pelusios_castaneus #0.245028 , Pelomedusa_subrufa #0.124007 ) #219.938 ) #0.0276329 , ((Chelus_fimbriata #0.0387087 , (Platemys_platycephala #0.0384226 , Mesoclemmys_hogei #0.0736725 ) #140.134 ) #0.0193127 , (((Elusor_macrurus #0.0001 , (Myuchelys_bellii #0.0999477 , Emydura_subglobosa #0.087947 ) #0.0001 ) #0.0605896 , (Chelodina_parkeri #0.0559397 , (Chelodina_rugosa #230.011 , (Chelodina_pritchardi #0.170101 , (Chelodina_mccordi #0.117526 , (Chelodina_longicollis #9.69264 , Chelodina_expansa #7.79852 ) #0.0001 ) #0.102013 ) #0.0001 ) #0.0001 ) #0.0133744 ) #0.0001 , Pseudemydura_umbrina #0.0558324 ) #0.0243866 ) #221.62 ) #0.73571 , ((Carettochelys_insculpta #0.0392709 , ((Lissemys_scutata #0.0704226 , Lissemys_punctata #196.845 ) #0.0570807 , ((Trionyx_triunguis #0.379627 , (Pelochelys_cantorii #0.259618 , Chitra_indica #0.108567 ) #0.0001 ) #0.0001 , ((Rafetus_swinhoei #0.165215 , (Apalone_spinifera #0.0001 , Apalone_ferox #0.102044 ) #0.0568404 ) #273.328 , ((Amyda_cartilaginea #0.0329596 , Dogania_subplana #0.0482977 ) #0.116103 , (Palea_steindachneri #0.0806657 , Pelodiscus_sinensis #0.0446916 ) #0.0001 ) #0.0302788 ) #0.0001 ) #0.093479 ) #0.0680988 ) #173.498 , (((Sternotherus_carinatus #240.581 , Kinosternon_leucostomum #0.0001 ) #0.215042 , (Macrochelys_temminckii #0.178365 , Chelydra_serpentina #0.0702452 ) #418.513 ) #213.139 , (((Platysternon_megacephalum #0.0269632 , (Chrysemys_picta #0.0226014 , (Trachemys_scripta #0.0938302 , Malaclemys_terrapin #0.106932 ) #0.0001 ) #0.0840928 ) #0.0001 , (((((Astrochelys_yniphora #0.036542 , Aldabrachelys_gigantea #0.0638978 ) #306.327 , Psammobates_pardalis #0.0801566 ) #81.4286 , ((((Indotestudo_forstenii #0.133939 , Indotestudo_elongata #209.716 ) #0.122203 , Testudo_horsfieldii #0.180333 ) #77.1169 , (Testudo_graeca #0.051186 , (Testudo_marginata #0.0996342 , Testudo_kleinmanni #0.0104941 ) #0.0245518 ) #0.0001 ) #0.0001 , Malacochersus_tornieri #0.115228 ) #0.273445 ) #0.0783772 , (Manouria_impressa #0.100446 , Manouria_emys #0.222082 ) #0.0877518 ) #0.093915 , ((Batagur_trivittata #0.143914 , Geoemyda_spengleri #0.125352 ) #0.0001 , ((((Notochelys_platynota #0.102358 , (Cyclemys_fusca #0.0001 , ((Cyclemys_tcheponensis #0.0001 , Cyclemys_oldhamii #0.0001 ) #0.150089 , (Cyclemys_dentata #0.0001 , (Cyclemys_pulchristriata #1.19861 , Cyclemys_atripons #0.0001 ) #111.964 ) #0.0001 ) #0.0001 ) #0.106882 ) #0.0001 , (Heosemys_annandalii #0.347705 , (Heosemys_grandis #0.0384079 , Heosemys_depressa #0.301814 ) #0.103229 ) #0.125697 ) #0.0001 , (Sacalia_quadriocellata #0.151248 , Sacalia_bealei #0.427222 ) #0.0890858 ) #220.145 , (((((Cuora_flavomarginata #0.113204 , (Cuora_trifasciata #445.938 , (Cuora_pani #0.147643 , Cuora_aurocapitata #0.381082 ) #1.79606 ) #0.297585 ) #0.0001 , (Cuora_mccordi #0.125923 , (Cuora_galbinifrons #0.0424652 , (Cuora_picturata #0.0001 , Cuora_bourreti #0.0001 ) #0.0519039 ) #0.0001 ) #0.0001 ) #0.0001 , Cuora_mouhotii #0.526722 ) #0.0001 , Cuora_amboinensis #0.152125 ) #0.0717134 , ((Mauremys_leprosa #0.222014 , (((Mauremys_japonica #2.25364 , Mauremys_sinensis #0.484533 ) #0.0001 , ((Mauremys_reevesii #1.06464 , NC_015101.1 #0.0001 ) #0.126716 , Mauremys_nigricans #0.0001 ) #0.310885 ) #0.460415 , (Mauremys_rivulata #2.62202 , Mauremys_caspica #2.20617 ) #0.0476491 ) #0.0001 ) #0.0001 , (Mauremys_mutica #0.0511986 , Mauremys_annamensis #0.0750128 ) #0.0001 ) #0.697609 ) #0.0669734 ) #284.569 ) #101.591 ) #0.0219555 ) #0.0001 , (Dermochelys_coriacea #0.0384514 , ((Eretmochelys_imbricata #0.471711 , (Caretta_caretta #0.0001 , (Lepidochelys_olivacea #0.0001 , Lepidochelys_kempii #0.24375 ) #0.046315 ) #0.19484 ) #0.0001 , (Chelonia_mydas #0.0134129 , Natator_depressa #0.0669487 ) #0.0001 ) #0.214346 ) #0.0612458 ) #0.185361 ) #0.144038 ) #0.0001 ) #0.00376624 );
